# Defective Ultrathin ZnIn_2_S_4_ for Photoreductive Deuteration of Carbonyls Using D_2_O as the Deuterium Source

**DOI:** 10.1002/advs.202103408

**Published:** 2021-11-19

**Authors:** Chuang Han, Guanqun Han, Shukai Yao, Lan Yuan, Xingwu Liu, Zhi Cao, Arun Mannodi‐Kanakkithodi, Yujie Sun

**Affiliations:** ^1^ Department of Chemistry University of Cincinnati Cincinnati OH 45221 USA; ^2^ School of Materials Engineering Purdue University West Lafayette IN 47907 USA; ^3^ School of Chemistry and Chemical Engineering Wuhan University of Science and Technology Wuhan 430081 China; ^4^ Syncat@Beijing Synfuels CHINA Company, Ltd. Beijing 101407 China

**Keywords:** carbonyls, deuteration, heavy water, noble metal‐free, photocatalysis

## Abstract

Deuterium (D) labeling is of great value in organic synthesis, pharmaceutical industry, and materials science. However, the state‐of‐the‐art deuteration methods generally require noble metal catalysts, expensive deuterium sources, or harsh reaction conditions. Herein, noble metal‐free and ultrathin ZnIn_2_S_4_ (ZIS) is reported as an effective photocatalyst for visible light‐driven reductive deuteration of carbonyls to produce deuterated alcohols using heavy water (D_2_O) as the sole deuterium source. Defective two‐dimensional ZIS nanosheets (D‐ZIS) are prepared in a surfactant assisted bottom‐up route exhibited much enhanced performance than the pristine ZIS counterpart. A systematic study is carried out to elucidate the contributing factors and it is found that the in situ surfactant modification enabled D‐ZIS to expose more defect sites for charge carrier separation and active D‐species generation, as well as high specific surface area, all of which are beneficial for the desirable deuteration reaction. This work highlights the great potential in developing low‐cost semiconductor‐based photocatalysts for organic deuteration in D_2_O, circumventing expensive deuterium reagents and harsh conditions.

## Graphical abstract

Surfactant‐assisted synthesis enables to obtain defective ultrathin ZnIn_2_S_4_ nanosheets which can act as noble metal‐free photocatalysts for effective photoreductive deuteration of carbonyls and imines to produce deuterated alcohols and amines using D_2_O as the sole deuterium source under ambient conditions.

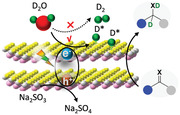

## 1 Introduction

**Scheme 1:**
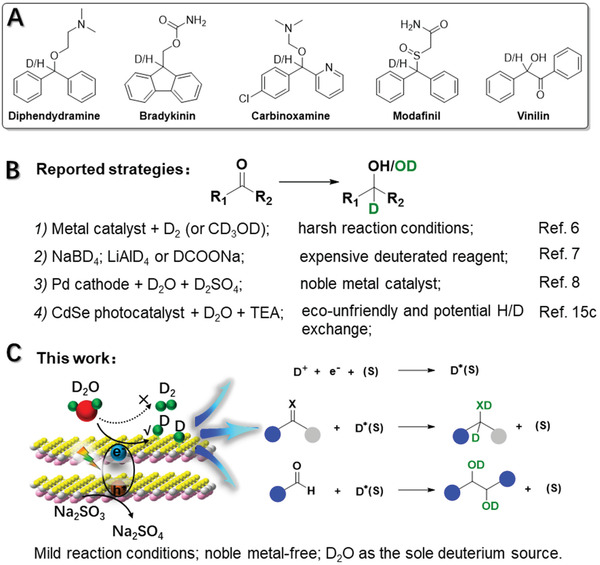
A) Representative examples of pharmaceuticals prepared from alcohols. B) Reported strategies for producing *α*‐deuterated alcohols from carbonyls. C) Our photocatalytic deuteration of carbonyls to produce alcohols using D_2_O as the sole deuterium source. TEA represents the triethylamine. (s) represents the surface sites.

Owing to the kinetic isotope effect, deuterium (D)‐labeled compounds find important applications in investigating reaction mechanisms,^[^ ^1^ ^]^ modifying selectivity in organic synthesis,^[^ ^2^ ^]^ and preparing advanced materials with enhanced performance.^[^ ^3^ ^]^ Deuterated alcohols are key intermediates in the synthesis of D‐labeled pharmaceuticals^[^ ^4^ ^]^ and can also serve as deuterium sources for the production of many other deuterated chemicals with applications in materials science (**Scheme** **1**A).^[^ ^5^ ^]^ Reductive deuteration of carbonyls is one of the most straightforward processes to deliver *α*‐deuterated alcohols. However, previously established strategies usually require either harsh reaction conditions, noble‐metal catalysts, or expensive deuterium sources (Scheme 1B). For example, reductive deuteration of C═O double bonds can be achieved with metal catalysts and D_2_ (or its precursors) at high temperature and elevated pressure.^[^ ^6^ ^]^ Even though alkali metal deuteride salts (e.g., NaBD_4_ and LiAlD_4_) can readily produce *α*‐deuterated alcohols from carbonyls,^[^ ^7^ ^]^ these deuterium donors are less economically attractive because of their high cost. Recently, Berlinguette et al. reported an electrochemical approach for the construction of C—D bonds in the deuterated electrolyte (D_2_O/D_2_SO_4_) under ambient condition.^[^ ^8^ ^]^ Nevertheless, this strategy relies on the employment of noble Pd‐based membrane as cathode and specifically deuterated solvent as electrolyte.

To produce *α*‐deuterated alcohols of large quantity in a cost‐effective manner, heavy water (D_2_O) is the most appealing deuterium source in terms of cost, abundance, and benign nature.^[^ ^5^, ^9^ ^]^ Inspired by the great advances achieved in photocatalytic/electrolytic water splitting, wherein the adsorbed H species (H*) are generated on the catalyst surface,^[^ ^10^ ^]^ it is expected that, when D_2_O is used as the solvent, it is highly feasible to realize photocatalytic/electrolytic deuteration of organics if the in situ formed D* can be used for deuteration instead of D_2_ evolution. Indeed, this aspiration has been explored for electrochemical deuteration of alkynes,^[^ ^8^, ^11^ ^]^ alkenes,^[^ ^8^ ^]^ halides,^[^ ^12^ ^]^ and aldehydes^[^ ^8^, ^13^ ^]^ on Pd, Cu, or Co‐based cathode.^[^ ^11^, ^12^ ^]^ And a few examples of semiconductors decorated with noble metal co‐catalysts have been reported for photoreductive deuteration of alkenes,^[^ ^14^ ^]^ alkynes,^[^ ^14b^ ^]^ nitroaromatic compounds,^[^ ^14a^ ^]^ and aldehyde.^[^ ^14a^ ^]^ However, the requirement of noble metal co‐catalysts not only inevitably leads to the high cost of the resulting photocatalysts but may also cause strong D_2_ evolution because of their high activity for water reduction,^[^ ^15^ ^]^ rendering low atom economy for the desirable deuteration reaction. It remains highly attractive to achieve organic deuteration using D_2_O as the deuterium source and inexpensive semiconductors without any noble metal co‐catalysts. Until recently, Wu and co‐workers described a photocatalytic strategy for deuteration of ketones with bare CdSe quantum dots as the photocatalyst (Scheme 1B).^[^ ^15c^ ^]^ However, this process requires an eco‐unfriendly catalyst and a protic sacrificial reagent (i.e., TEA), which may lower the D incorporation efficiency due to the inevitable H/D exchange between D_2_O and TEA.

With these considerations in mind, we were intrigued by the recent success of defect engineering of semiconductors to enhance their photocatalytic performance in various fields.^[^ ^16^ ^]^ In situ surface modification during semiconductor preparation can reduce the size and/or alter the morphology, and create more defect sites on the surface of the final semiconductors.^[^ ^17^ ^]^ Those defect sites can trap the photogenerated charge carriers and promote the adsorption of active species.^[^ ^18^ ^]^ For example, Lau et al. reported that the photogenerated H* could be confined within the heptazine units in cyanamide‐modified g‐C_3_N_4_ and later released in the dark to yield H_2_.^[^ ^19^ ^]^ Furthermore, density functional theory (DFT) suggests that the surface sites, especially for the vacancy have a great impact on the H* adsorption energy of transition‐metal chalcogenides.^[^ ^20^ ^]^ Given that the efficient charge separation and H*/D* generation are favorable for photocatalytic hydrogenation/deuteration, these results unambiguously prove that engineering semiconductors with surface defects could improve charge separation, expose more active sites, and create long‐lived active species for our target photoreductive deuteration reactions without the dependence on noble metal co‐catalysts.

Herein, we report an inexpensive and noble metal‐free photocatalyst, defective ultrathin ZnIn_2_S_4_ nanosheets (D‐ZIS) with abundant S vacancy, for visible light‐driven reductive deuteration of carbonyls to alcohols using D_2_O as the deuterium source and Na_2_SO_3_ as the aprotic sacrificial reagent (Scheme 1C). The in situ modification with surfactant in the synthesis of ZnIn_2_S_4_ (ZIS) results in ultrathin two‐dimensional (2D) morphology and abundant defect sites in D‐ZIS, which greatly promote interfacial charge transfer and the formation of confined D* for the subsequent deuteration reactions, instead of D_2_ evolution. Different from previous reported defective semiconductors for photocatalytic H_2_ evolution or CO_2_ reduction,^[^ ^21^ ^]^ it is the first work to show how defect engineering can promote photocatalytic hydrogenation/deuteration reactions.

## 2 Results and Discussion

Using benzophenone as a testing substrate, we first screened the performance of three representative semiconductors, ZIS, CdS, and C_3_N_4_ (Figure S1A, Supporting Information). Under visible light (*λ* > 420 nm) irradiation, all the samples exhibited activity towards the H_2_ evolution reaction (HER), a major competing reaction for hydrogenation, in agreement with their suitable band alignment (Figure S1B, Supporting Information). Production of the desirable benzhydrol could only be detected when ZIS and CdS were used as photocatalysts. We noticed that the yield of benzhydrol was inversely related to their HER activity and closely correlated with the adsorption energy of H* on these semiconductors (Figure S1C, Supporting Information). Among the three semiconductors, ZIS possesses the least reduction power and strongest H* adsorption, thus leading to inferior HER performance while better hydrogenation capability.^[^ ^20^, ^22^ ^]^ Hence, the subsequent work will focus on ZIS as the desirable photocatalyst.

**Figure 1:**
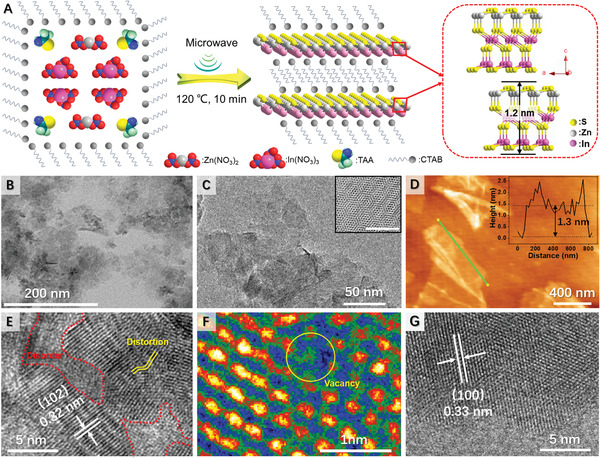
A) Schematic preparation of D‐ZIS. B) TEM, C) TEM, D) AFM, E) HR‐TEM, and F) false‐color HR‐TEM images of D‐ZIS. G) HR‐TEM image of ZIS. Insets of (C) and (D) show the Moiré pattern and height profile of ultrathin D‐ZIS nanosheets, respectively.

Even though ZIS showed excellent selectivity towards benzophenone hydrogenation over HER, the benzyhydrol yield was still very low (˂ 10%). As a unique member of the AB_2_X_4_ family semiconductors with a layered structure, the hexagonal ZIS consists of the packet stacking of S−Zn−S−In−S−In−S layers.^[^ ^21b^ ^]^ Theoretically, the photocatalytic activity of ZIS can be further optimized by adjusting the layer thickness and surface atom configuration. Herein, we sought to engineer ZIS via molecular modification.^[^ ^21^, ^23^ ^]^ Following a facile cetyltrimethylammonium bromide (CTAB)‐assisted microwave synthetic scheme (**Figure** **1**A and see the Supporting Information for details), we were able to obtain ultrathin D‐ZIS nanosheets, which were anticipated to have large specific surface area. In addition, surface atoms could more easily escape from the 2D lattice to create more surface vacancy, which may improve charge carrier transfer, inhibit exciton recombination, and enrich active sites.^[^ ^24^ ^]^

The transmission electron microscopy (TEM) images of as‐prepared D‐ZIS confirm its uniform sheet‐like morphology (Figure 1B,C and Figure S2A,B, Supporting Information). The hexagonal Moiré pattern resulting from interference between the crystalline lattices of the stacked nanosheets in D‐ZIS could also be observed (inset of Figure 1C), which further confirmed its 2D ultrathin structure.^[^ ^25^ ^]^ In fact, the thickness of D‐ZIS was estimated to be ≈1.3 nm (Figure 1D) based on the atomic force microscopy (AFM) analysis. Such a thin thickness was consistent with the size of a half unit cell of ZnIn_2_S_4_ along the [001] axis (model of Figure 1A).^[^ ^21a,b^ ^]^ High‐resolution TEM (HR‐TEM, Figure 1E) analysis of D‐ZIS revealed the co‐existence of “two phases”: well‐defined crystalline regions with distinct lattice fringes (0.32 nm) and highly disordered “nano‐islands” with the distorted crystal lattice (marked with red and yellow lines). The missing of some atoms can be clearly seen from the false‐colour HR‐TEM image (yellow circle in Figure 1F), suggesting the existence of vacancy.^[^ ^21a,b^ ^]^ In contrast, ZIS nanosheets prepared in the absence of CTAB surfactant showed multi‐stacked structure (Figure S2C,D, Supporting Information) and well‐defined lattice fringes with d‐spacings of 0.33 and 0.32 nm (Figure 1G and Figure S2E, Supporting Information), corresponding to the (100) and (102) facets of ZnIn_2_S_4_, respectively.^[^ ^26^ ^]^ AFM analysis (Figure S2F, Supporting Information) of ZIS revealed its thickness of ≈2.1 nm. It is rationalized that CTAB could be adsorbed on the layer surfaces to prevent stacking, hence D‐ZIS is thinner than ZIS. In fact, the infrared (IR) spectrum of D‐ZIS indeed presents the signature feature of the alkyl chain of CTAB (Figure S3, Supporting Information).^[^ ^27^ ^]^

**Figure 2:**
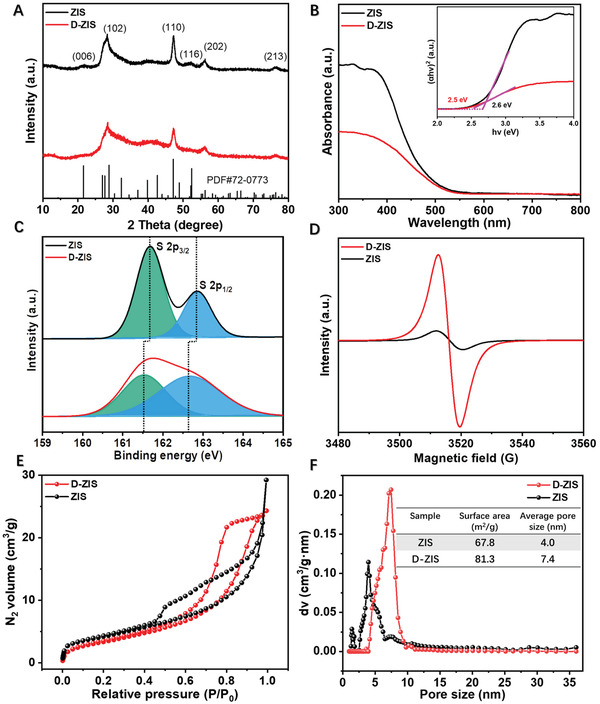
A) XRD, B) UV–vis DRS, C) S 2p high‐resolution XPS spectra, D) EPR spectra, E) N_2_ adsorption‐desorption isotherms, and F) pore size distribution of ZIS and D‐ZIS. Insets of (B) and (F) respectively show the plot based on the Kubelka‐Munk function and the summary of BET analysis.

X‐ray diffraction (XRD) was further performed to evaluate the structural and crystallinity changes of ZIS prepared in the absence and presence of CTAB. As shown in **Figure** **2**A, the bare ZIS shows diffraction peaks at 21.7, 28.0, 47.3, 52.7, 55.6°, and 76.4°, which can be attributed to the (006), (102), (110), (116), (202), and (213) lattice planes of the hexagonal ZIS (JCPDS No. 72–0773),^[^ ^28^ ^]^ respectively. D‐ZIS possesses a similar hexagonal structure but lower crystallinity, consistent with its thinner thickness and the observation of “two‐phases” in HR‐TEM images (Figure 1E). There is no huge difference in the light absorption threshold between D‐ZIS and bare ZIS. As suggested in the ultraviolet‐visible diffuse reflectance spectroscopy (UV–vis DRS) plots in Figure 2B, only a slight red shift was observed in the absorption onset for D‐ZIS (2.5 eV) relative to ZIS (2.6 eV).^[^ ^18a^ ^]^

Furthermore, X‐ray photoelectron spectroscopy (XPS, Figure 2C) of D‐ZIS demonstrates that its S 2p feature is broader and the S 2p_3/2_ (161.5 eV) and 2p_1/2_ (162.5 eV) peaks are negatively shifted by 0.2 and 0.3 eV, respectively, relative to those of ZIS (161.7 and 162.8 eV). While the In 3d and Zn 2p spectra (Figure S4, Supporting Information) of D‐ZIS are slightly positively shifted compared to those of ZIS. These variations could be attributed to the presence of S vacancy, which leads to the changes of electron density around S, In, and Zn.^[^ ^21a,b^ ^]^ The element contents in ZIS and D‐ZIS were quantified by XPS and inductively coupled plasma (ICP) methods.^[^ ^21^, ^29^ ^]^ The atomic ratio of S in D‐ZIS is decreased compared to that in ZIS (Table S1, Supporting Information), implying a higher S vacancy density in D‐ZIS. The existence of S vacancy in D‐ZIS was also confirmed by the electron spin resonance (ESR) spectra shown in Figure 2D, wherein D‐ZIS shows a stronger ESR signal at a g‐value of 2.003 due to the presence of electron trapped at S vacancy.^[^ ^21^, ^30^ ^]^ Consistent with their ultrathin 2D morphology, D‐ZIS nanosheets exhibit a high specific surface area based on the nitrogen adsorption‐desorption isotherm measurements (Figure 2E,F).^[^ ^31^ ^]^ Compared with ZIS, D‐ZIS shows more ordered pore distribution and larger surface area and pore size due to the pore‐blocking/percolation induced by CTAB.^[^ ^32^ ^]^ Based on the above results, it is reasonable that the CTAB molecule can adsorb on the surface of the primary nanocrystal during the process of catalyst preparation, which can partially hinder the growth of crystal and lead to the formation of the ultrathin structure with vacancy.

The impact of CTAB in situ modification on the photocatalytic activity of the resulting D‐ZIS was apparent and it was found that the optimal weight ratio between CTAB and ZIS was 3:1 (Figure S5, Supporting Information). Systematic optimization of the reaction condition, including the amount of Na_2_SO_3_ and the solvent ratio between acetonitrile (MeCN) and H_2_O (or D_2_O), was performed (Table S2, Supporting Information). Under the optimal condition, a 92% yield of benzhydrol could be achieved in MeCN/H_2_O (v/v = 1/9), and a slightly decreased yield of 88% with a high deuteration incorporation ratio (> 98%) was realized when D_2_O was used to replace H_2_O. Simply mixing CTAB and ZIS in the reaction system only resulted in a much lower yield (12%). After washing D‐ZIS several times with dichloromethane, most adsorbed CTAB was removed as confirmed by the Infrared spectrum (Figure S6, Supporting Information), and the resulting D‐ZIS still showed a high yield (82%). These results imply that the beneficial effect of CTAB is for the formation of ultrathin nanosheet structure of D‐ZIS with abundant defects, not directly involved in the subsequent photocatalysis process.

**Figure 3:**
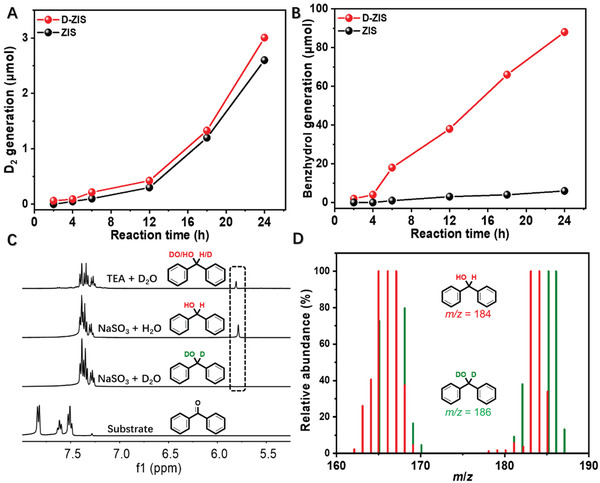
A) D_2_ and B) benzhydrol production on ZIS and D‐ZIS under visible light (*λ* > 420 nm) irradiation over time. C) ^1^H NMR spectra of benzophenone and post‐photolysis samples collected under different reaction conditions. D) MS spectra of the isolated products obtained in MeCN/H_2_O or MeCN/D_2_O.

We also noticed that the production rates of both D_2_ and benzhydrol were very slow during the first 4 h of irradiation (**Figure** **3**A,B) on D‐ZIS. Prolonging the reaction time led to a steady increase of D_2_ and benzyhydrol yields (Figure S7, Supporting Information). Even though the bare ZIS was able to produce D_2_ in a similar fashion as D‐ZIS, its yield of benzyhydrol (8%) remained extremely low even after 24 h irradiation. Overall, D‐ZIS showed a much higher total electron utilization efficiency and electron consumption percentage for target deuteration (97%) than bare ZIS (69%, Figure S8, Supporting Information).

It should be noted that the utilization of Na_2_SO_3_ is crucial for the successful deuteration of benzophenone. If TEA, a popular sacrificial reagent in photocatalysis,^[^ ^33^ ^]^ was used instead, a much lower deuteration incorporation ratio (40%) was observed (Figure 3C). These results indicate that apart from severing as an electron donator, TEA could also act as a proton source. Similarly, it was well expected that no deuterated product would be detected if the photocatalysis was performed in MeCN/H_2_O. Mass spectroscopy (MS) analysis demonstrated that the *m/z* values of 167 and 184 could be assigned to the fragmentation ion [Ph–CH–Ph]^+^ and the molecular ion of benzhydrol, respectively (Figure 3D), which shifted to 168 and 186 when the reaction was carried out in MeCN/D_2_O, further confirming D_2_O as the sole deuteration source.

**Figure 4:**
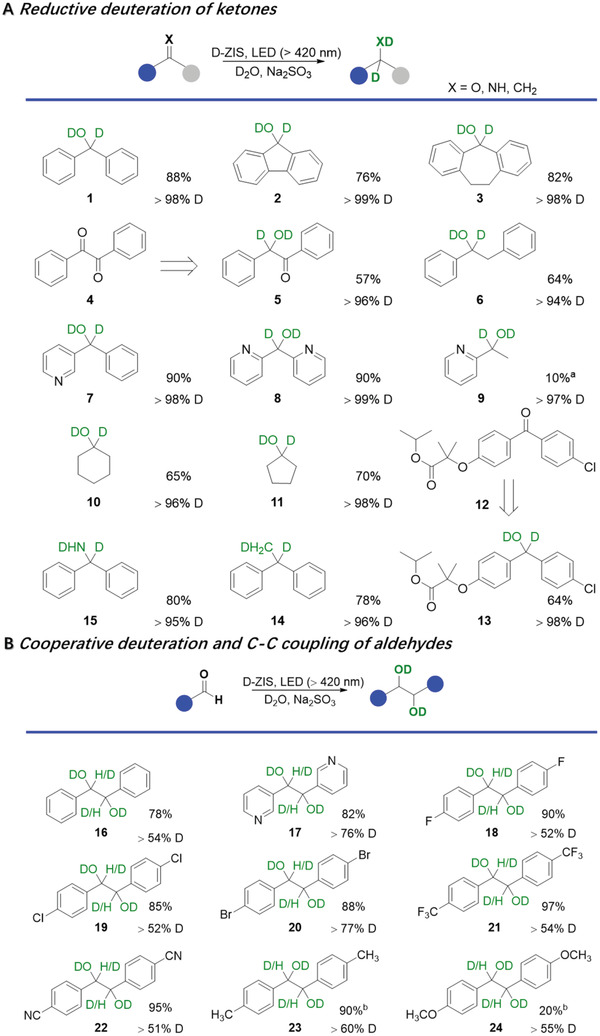
Reaction scopes of A) photocatalytic reductive deuteration of ketones and B) cooperative deuteration and C–C coupling of aldehydes. Reaction condition: 0.1 mmol substrate, 5 mg D‐ZIS, 0.2 mL MeCN, 1.8 mL D_2_O, 2 mmol Na_2_SO_3_, irradiation with visible light (*λ* > 420 nm). Unless stated otherwise, the reaction times for (A) and (B) were 24 and 8 h, respectively. Note: ^a^Substrate conversion was 100% and C–C coupling product was detected; ^b^The irradiation time was 24 h and monoalcohols were produced.

With the optimal reaction condition in hand, we next expanded the substrate scope for the photocatalytic reductive deuteration of carbonyls. As shown in **Figure** **4**A, close analogues of benzophenone could yield desirable deuterated alcohols with decent yields from 76% (2) to 82% (3). In the case of benzil, only one deuterated alcohol group was formed while the other carbonyl remained intact (4). Asymmetric aromatic ketones and pyridine‐containing substrates could also be successfully transformed to the corresponding deuterated alcohols (6–8) with good to excellent yields (64–90%). However, only 10% of the deuterated alcohol (9) was obtained from acetylpyridine, while pinacol and other unknown products were also produced. Even though aliphatic ketones are more inert than their aromatic counterparts, our D‐ZIS was able to yield cyclohexanol (10) and cyclopentanol (11) with 65% and 70% yields, respectively. When Fenofibrate (12), a prescription medicine for the treatment of high cholesterol and triglycerides, was employed, its deuterated alcohol product 13 was obtained in a yield of 64%, highlighting the potential of our photocatalyst in pharmaceutical applications. Furthermore, the reductive deuteration capability of D‐ZIS could also be extended to other C═X bonds, including alkenes and imines. For instance, 1,1‐diphenylethene and 1,1‐diphenylmethanimine were successfully converted to deuterated 1,1‐diphenylethane (14, 78%) and 1,1‐diphenylmethanamine (15, 80%), respectively. In other related works, the photoreductive deuteration of imines was less studied.^[^ ^14^, ^15^, ^34^ ^]^ The achievement in the production of 15 encouraged us to explore the universality of the present strategy towards deuteration of other imine substrates. To our delight, the deuterated amine can be produced from benzophenone ketimine with a moderate yield (60%, Figure S9, Supporting Information). For diaryl imine (Figure S10, Supporting Information) and monoaryl imine (Figure S11, Supporting Information), both substrates underwent homocoupling to produce deuterated ethylene diamine products with decent yields and deuteration incorporation ratios.

In terms of aromatic aldehydes, reductive deuteration followed by C–C coupling of the in situ formed ketyl radicals takes place on D‐ZIS. The resulting deuterated pinacols were produced with a variety of substituents at the *para* position of the phenyl ring. As expected, electron‐withdrawing groups could facilitate the reduction of the aldehyde groups (18–22) while electron‐donating groups suppress its reduction (23–24).^[^ ^35^ ^]^ Apart from C–C coupling products, monoalcohols as byproducts were produced when the substituent was an electron‐donating group (Figure S12, Supporting Information). It's also noted that the deuterium was more inclined to bond with O instead of C in the final pinacol products (Figure S13, Supporting Information). Although the O‐H/O‐D exchange process by hydrogen isotope exchange (HIE) strategy can also produce deuterated pinacol, it may suffer from limited scope (starting from pinacol), poor functional group tolerance and/or nonselective multiposition labeling. In contrast, this photocatalytic deuteration system can be employed to reductively deuterate ketones and aldehydes, as well as alkenes and imines, thus our approach is complementary to the HIE method and allows for isotope labeling at the starting material stage.

Furthermore, D‐ZIS exhibited no apparent activity decrease for five consecutive photocatalysis cycles of benzophenone deuteration with a total irradiation time of 120 h (Figure S14A, Supporting Information). Post‐photocatalysis characterization also suggested that the used sample maintained similar 2D ultrathin nanosheet morphology (TEM and AFM images in Figure S14B–E, Supporting Information) and crystallinity (XRD in Figure S14F, Supporting Information) as the pristine D‐ZIS. The slight aggregation of the nanosheets led to the marginally decreased BET surface area (Table S3, Supporting Information), but the density of S vacancy (Table S3, Supporting Information) was slightly increased, which might result from the lattice sulfide ion oxidation induced by the photoexcited holes. These results corroborate the strong robustness of our D‐ZIS for long‐term application.

**Figure 5:**
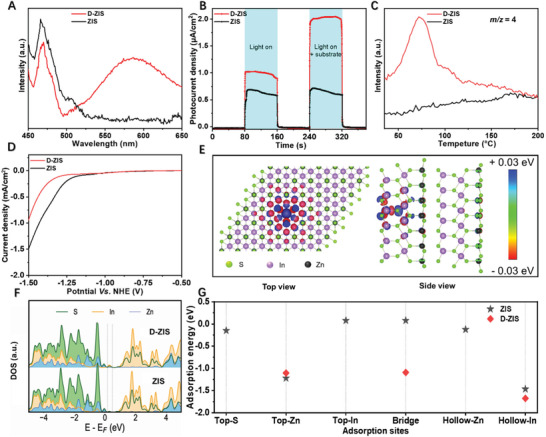
A) PL emission spectra with an excitation wavelength of 340 nm over ZIS and D‐ZIS. B) Photocurrent density versus time (*I‐t*) curves under visible light (*λ* > 420 nm) irradiation and C) MS signals at *m/z* = 4 during the D_2_‐TPD analyses over ZIS and D‐ZIS. D) LSV curves of ZIS and D‐ZIS electrodes collected in the dark. E) The top view and side view show the charge density difference around the S vacancy center in D‐ZIS. F) The density of states for ZIS and D‐ZIS. G) Calculated H* adsorption energies for different sites in ZIS and D‐ZIS slab structures.

Next, we sought to elucidate the factors contributing to the superior photocatalytic performance of D‐ZIS compared to the bare ZIS. **Figure** **5**A presents their photoluminescence (PL) spectra, wherein the emission feature at 470 nm could be assigned to the rapid recombination of electron‐hole pairs generated from bandgap transition^[^ ^18b^ ^]^ while the 500 nm peak was due to the defect site‐related transitions.^[^ ^36^ ^]^ The reduced bandgap transition while much enhanced defect site‐based transition in D‐ZIS versus ZIS could be rationalized by the presence of more disordered sites in the former.^[^ ^37^ ^]^ Significant PL quenching of both bandgap transition and defect‐related emission were observed for D‐ZIS upon the addition of benzophenone (Figure S15, Supporting Information). However, only a slight emission decrease was detected for ZIS, further implying that the excited electrons on the surface of D‐ZIS nanosheet could be effectively utilized for the reduction of benzophenone.^[^ ^14^, ^18^, ^21^ ^]^ As a complementary study, we prepared two electrodes coated with D‐ZIS and ZIS, respectively. As shown in Figure 5B, D‐ZIS showed a higher photocurrent response than ZIS, suggesting more efficient excited electron‐hole separation and interfacial electron transfer.^[^ ^38^ ^]^ Upon the addition of benzophenone, a much higher photocurrent was observed for D‐ZIS while no apparent change could be detected on the ZIS electrode, further indicating the former was more effective in reducing benzophenone.

Secondly, the high specific surface area and abundant defect sites on the surface of D‐ZIS may also provide more sites for the generation and adsorption of active D/H‐species.^[^ ^19^, ^39^ ^]^ As shown in Figure 5C, the temperature‐programmed desorption (TPD) curve of D‐ZIS after irradiation in D_2_O disclosed a distinct D_2_ desorption peak (*m/z* = 4) within 45–120 °C. In contrast, no apparent feature could be detected for ZIS. These D_2_‐TPD results suggest that more adsorbed D* species could be stored on D‐ZIS for later chemical reaction, instead of direct and undesirable D_2_ evolution. The inferior activity of D‐ZIS towards H_2_ evolution could be further suggested from its linear sweep voltammogram (LSV, Figure 5D). A more negative onset potential was observed for H_2_ evolution on the electrode coated with D‐ZIS (−1.3 V vs NHE) relative to ZIS (−1.2 V vs NHE).

Since the reduction potentials of benzophenone and benzaldehyde were measured at −1.2 and −1.3 V versus NHE (Figure S16, Supporting Information), respectively, which are more negative than the conduction band energy of ZIS,^[^ ^40^ ^]^ it is impossible for direct one‐electron reduction (inset of Figure S16, Supporting Information) to take place for these organics on our photocatalyst. Instead, a well‐known proton‐coupled electron transfer process is anticipated to occur.^[^ ^41^ ^]^ More precisely, the in situ formed protons/deuterons on D‐ZIS interact with carbonyls to form ketyl radicals. Due to the steric effect, diphenyl ketyl radicals will accept another H*/D* to give alcohols, however monoaromatic ketyl radicals will go through the C−C coupling path to produce pinacols. To support the above hypothesis, we carried out another control experiment outlined in Figure S17 (Supporting Information). D‐ZIS was first irradiated for 24 h in D_2_O in the absence of any organic substrate. Subsequently, benzophenone was added and the reaction vial was kept in the dark for another 24 h. Eventually, deuterated benzhydrol could be detected with a yield of 15% (Figure S18, Supporting Information). This result unambiguously proved that the in situ formed D* could be accumulated on D‐ZIS for a later chemical reaction. In fact, this delayed deuteration process was also accompanied by the color change of the photocatalyst. D‐ZIS changed from yellow to gray and turned back to yellow after reaction with benzophenone. According to previous reports,^[^ ^21^, ^39^ ^]^ this is due to the fact that the defect sites in semiconductors act as electron traps, which further produce the D‐species adsorbed on the surface of photocatalysts.

In order to unravel the formation and influence of S vacancy on the electronic structure and consequently the photocatalytic properties of D‐ZIS, we performed DFT computations. The fully optimized crystal structure was used to determine the electronic density of states and charge density distributions around atoms, as well as for building defect structures (Figure S19A, Supporting Information). Firstly, the defect formation energy was calculated based on the total DFT energy of each ZIS and D‐ZIS structures, as well as the chemical potentials of the species contributing to the defect. Figure S19B (Supporting Information) shows the calculated formation energies for different defects. The single S vacancy shows formation energy ranging from −0.35 to 2.46 eV under different chemical potential conditions (µ). The moderate formation energy suggests that S vacancy is readily created and well‐maintained in In‐bridge (V_s2_) sites of D‐ZIS.

To examine the electronic properties of ZIS with S vacancy, we calculated the charge density distributions around different atoms and determined the electronic density of states (DOS) of atoms in the vicinity of the defect. Figure 5E shows the top view and side view of the electron density distribution in D‐ZIS with S vacancy in In‐bridge sites (see Figure S20, Supporting Information, for other defect sites). It suggests that a net positive charge distribution (colored blue) is created around the defect center, where the S atom has been removed. Thus, the S vacancy may act as an electron vortex to trap photoexcited electrons for charge balance.^[^ ^21^, ^36^, ^42^ ^]^ Charge distributions are also clearly changed around the nearby In and Zn atoms, as can be seen from the blue and red colors appearing and vanishing, as we move farther away from the defect center. We then picked several In and S atoms around the S vacancy center and plotted their DOS as a function of energy in Figure 5F. Comparing the DOS from pure ZIS to D‐ZIS, it can be seen that the presence of a S vacancy creates mid‐gap energy states attributed to both S(p) state and In(p) state. This shows that S vacancy will modify the ZIS electronic structure drastically by creating shallow trap states around the Fermi energy, which could promote interfacial electron transfer.^[^ ^21^, ^42^ ^]^

Finally, we studied how the H* adsorption energy change with the presence of S vacancy. H atom was simulated at six different sites on the ZIS (110) surface, including top‐S, Zn, and In sites, bridge and hollow sites around Zn and In atoms, as shown in Figure S21 (Supporting Information). The computed adsorption energies are plotted in Figure 5G. More negative adsorption energy value implies more favorable H* adsorption. The relatively stable adsorption sites are found to be the hollow‐In site and hollow‐Zn site, with the H* adsorption energy of −1.47 and −1.22 eV, respectively. The H* adsorption energies at other sites are very weak and in the range of −0.15 to +0.08 eV. Based on these results, we selected three typical sites (i.e., top‐Zn, bridge, and hollow‐In sites) and performed adsorption calculations again for the D‐ZIS structure. The lowest and highest H* adsorption energies for D‐ZIS are found to be −1.68 and −1.09 eV in the hollow‐In and bridge sites, respectively. These values are lower than that on blank ZIS. It is expected that moderate adsorption energy of H* is favored to promote the hydrogenation reaction.^[^ ^14^, ^20^, ^23^ ^]^ Strong H* adsorption may inhibit the adsorption and formation of other reaction intermediates, whereas weak H* adsorption leads to serious HER.^[^ ^19^, ^33^, ^39^ ^]^ In this respect, the adsorption of H/D* on top‐Zn and bridge sites in D‐ZIS seems to be beneficial for the target hydrogenation/deuteration reactions. The above DFT calculations suggest that the presence of S vacancy is able to improve the photoactivity of D‐ZIS through optimizing its electronic properties and adsorption energies of H/D*.

## 3 Conclusion

In summary, we reported a noble metal‐free D‐ZIS photocatalyst for effective photocatalytic deuteration of carbonyls using D_2_O as the sole deuterium source. Different from the parent ZIS, D‐ZIS was rationally designed and fabricated from a surfactant‐assisted bottom‐up route. Such a molecular modification strategy enables D‐ZIS to exhibit superior photocatalytic activity for the deuteration of carbonyl groups in aromatic (and aliphatic) ketones and aldehydes, as well as alkenes and imines. A systematic investigation was carried out to shed light on the enhanced performance of D‐ZIS versus ZIS, wherein more defect sites on the catalyst surface, faster excited electron‐hole separation and interfacial charge transfer, and higher specific surface area all play important roles in the photocatalytic process. We believe that this light‐driven deuteration strategy in D_2_O can be applied to the synthesis of many other chemicals and pharmaceuticals, while D‐ZIS will be regarded as a promising photocatalyst for organic deuteration reactions.

## Conflict of Interest

The authors declare no conflict of interest.

## Supporting information

Supporting Information

## Data Availability

Research data are not shared.
