# Supplementary material for: Defective Ultrathin ZnIn2S4 for Photoreductive Deuteration of Carbonyls Using D2O as the Deuterium Source
Source: Adv Sci (Weinh). 2021 Nov 19;9(3):2103408. doi: 10.1002/advs.202103408 (PMC8787392; doi:10.1002/advs.202103408)
Supplement: Supplementary file 1 — Supporting Information [file ADVS-9-2103408-s001.pdf]

WILEY-VCH

ADVANCED SCIENCE
Open Access

Supporting Information

for *Adv. Sci.*, DOI: 10.1002/advs.202103408

Defective ultrathin $ZnIn_2S_4$ for photoreductive deuteration of carbonyls using D_2O as the deuterium source

Chuang Han, Guanqun Han, Shukai Yao, Lan Yuan, Xingwu Liu, Zhi Cao, Arun Mannodi-Kanakkithodi*, and Yujie Sun**

Supporting Information

Defective ultrathin $ZnIn_2S_4$ for photoreductive deuteration of carbonyls using D_2O as the deuterium source

Chuang Han, Guanqun Han, Shukai Yao, Lan Yuan, Xingwu Liu, Zhi Cao, Arun Mannodi-Kanakkithodi*, and Yujie Sun**

Table of Contents

Experimental section
Figure S1. (A) Benzhydrol and H_2 production under visible light ($\lambda > 420$ nm) irradiation of 24 h over ZIS, CdS and C_3N_4 semiconductors. (B) Band alignment of ZIS, CdS and C_3N_4 and redox potential of involved redox half-reactions in this work. (C) Free energy change (ΔG) for HER over ZIS, CdS and C_3N_4. Data in (B) and (C) are cited from ref.[1] and ref.[2], respectively.
Figure S2. TEM images of (A, B) D-ZIS and (C, D) blank ZIS nanosheets. (E) HR-TEM and (F) AFM images of blank ZIS nanosheets. Inset of (F) shows the height profile.
Figure S3. IR spectra of CTAB and D-ZIS.
Figure S4. XPS spectra of (A) In 3d and (B) Zn 2p in D-ZIS and ZIS.
Figure S5. Yields of benzhydrol over D-ZIS samples with different weight ratios of CTAB vs. ZIS in catalyst preparation. Reaction condition: 0.1 mmol substrate, 5 mg catalyst, 0.2 mL MeCN, 1.8 mL D_2O, 2 mmol Na_2SO_3, irradiation with visible light ($\lambda > 420$ nm) of 24 h.
Figure S6. IR spectra of CTAB, D-ZIS and D-ZIS after washing.
Figure S7. (A) ^1H NMR spectra and (B) GC signal of D_2 showing the evolution of the product during the deuteration reaction with different times.
Figure S8. Total electron generation and the electron consumption percentage (i.e., selectivity) toward benzhydrol production on ZIS and D-ZIS.
Figure S9. ^1H NMR spectra showing the products during the photocatalytic deuteration of benzophenone phenylimine.
Figure S10. ^1H NMR spectra showing the products during the photocatalytic deuteration of diphenylimine.
Figure S11. ^1H NMR spectra showing the products during the photocatalytic deuteration of N-benzylidenebenzylamine.
Figure S12. ^1H NMR spectra showing the products during the photocatalytic deuteration of (A) 4-methyl benzaldehyde and (B) 4-methoxybenzaldehyde.
Figure S13. ^1H NMR spectra showing the product of hydrobenzoin during the photocatalytic deuteration of benzaldehyde.
Figure S14. (A) Recycling test of D-ZIS. Reaction condition: 0.1 mmol benzophenone, 5 mg D-ZIS, 0.2 mL MeCN, 1.8 mL D_2O, 2 mmol Na_2SO_3, irradiation of 24 h with visible light ($\lambda > 420$ nm) in each round. (B and C) TEM images, (D) AFM image, (E) corresponding height profile and (F) XRD of D-ZIS after five consecutive cycles of benzophenone deuteration. For comparison, the XRD of D-ZIS before the reaction is also included in (F).
Figure S15. Photoluminescence spectra of different samples with or without benzophenone upon excitation at 340 nm.
Figure S16. Cyclic voltammograms of 10 mM benzophenone and 10 mM benzaldehyde.

Figure S17. Schematic and photographs showing the stepwise deuteration of benzophenone with D_2O under controlled conditions.
Figure S18. ^1H NMR spectrum of the crude reaction mixture after reaction of 24 h under dark. For comparison, the ^1H NMR spectrum of benzophenone before the reaction is also included.
Figure S19. (A) The optimized structure of hexagonal $ZnIn_2S_4$ with different S vacancies employed in DFT computations. (B) Defect formation energies corresponding to various vacancies in panel (A).
Figure S20. The electron density distribution around D-ZIS with different defect states corresponding to **Figure S19A**.
Figure S21. The models of H* adsorption on different sites of ZIS (110) surface.
Table S1. Elemental analyses of ZIS and D-ZIS.
Table S2. Yields of benzhydrol obtained under different reaction conditions.
Table S3. BET surface areas and elemental analyses of fresh D-ZIS and used D-ZIS after recycling test.
Supplemented NMR spectra
References

Experimental section

1. Materials. All reagents were obtained from commercial sources and used as received without further purification. Deionized water used in the synthesis process was obtained from local sources. Organic solvents were dried using molecular sieves.

2. Preparation of D-ZIS. D-ZIS was synthesized following a microwave-assisted hydrothermal method. Briefly, 0.0595 g $Zn(NO_3)_2 \cdot 6H_2O$, 0.1203 g $In(NO_3)_3 \cdot xH_2O$, 0.2404 g thioacetamide (TAA) and different amounts of CTAB were dissolved in 20 mL water. The solution was stirred for 1 h at room temperature and later subjected to microwave treatment at 120 °C for 10 min. Subsequently, the prepared D-ZIS was washed with water and ethanol thoroughly via centrifugation and dried at 60 °C. The control sample ZIS was prepared similarly without CTAB.

3. Preparation of CdS. 0.0913 g $CdCl_2 \cdot 2.5H_2O$ and 0.2404 g TAA were suspended in 20 mL water. After stirring for 1 h at room temperature, the solution was subjected to microwave treatment at 120 °C for 10 min. After cooling to room temperature naturally, the obtained products were collected by centrifugation and washed with water and ethanol for several times, and finally dried at 60 °C.

4. Preparation of C_3N_4. In a typical process, melamine was heated in Ar at 550 °C for 4 h with a ramp rate of 2 °C/min. The resultant yellow agglomerates were milled into C_3N_4 powder in a mortar and then washed with water, collected by centrifugation, and finally dried at 60 °C.

5. Characterizations. The morphology of samples was determined by scanning electron microscopy (SEM) on a Hitachi New Generation cold field emission SEM SU-8010 spectrophotometer. Transmission electron microscopy (TEM) images were obtained using a JEOL model JEM 2010 EX instrument at an accelerating voltage of 200 kV. A Nanoscope IIIA system was used to measure the atomic force microscopy (AFM) spectra. Samples were dispersed on a mica plate for the AFM measurement. The X-ray diffraction (XRD) patterns of the samples were collected on a Bruker D8 Advance X-ray diffractometer with Ni-filtered Cu Kα radiation at 40 kV and 40 mA in the 2θ ranging from 10° to 80° with a scan rate of 0.02° per second. The optical properties of the samples were analysed by ultraviolet-visible (UV-vis) diffuse reflectance spectroscopy (DRS) using a UV-vis spectrophotometer (Thermo Scientific Evolution 200 Series), in which $BaSO_4$ was employed as the internal reflectance standard. X-ray photoelectron spectroscopy (XPS) was performed on a Thermo Scientific ESCA Lab 250 spectrometer. In XPS analysis, all of the binding energies were calibrated by the C 1s peak at 284.6 eV. Electron paramagnetic resonance (EPR) spectroscopic measurements were performed at room temperature using a Bruker A300 EPR spectrometer. Elemental analysis was measured by inductively coupled plasma method (ICP, PerkinElmer Optima 2000DV). N_2 adsorption-desorption isotherms and the Brunauer-Emmett-Teller (BET) surface areas were measured at 77 K on an autosorb iQ apparatus. The photoluminescence spectra were collected on a Carry 50 spectrophotometer with excitation at 340 nm. A chemisorption analyser (Micromeritics Autochem 2920) was used to conduct D_2-temperature-programmed desorption (TPD) measurements. The sample after reaction was dried with N_2, and then analysed at 50 – 500 °C. The signal of the thermal conductivity detector (TCD) and mass signals of m/z = 4 was recorded. To ensure the comparability of the photoluminescence spectra, the experimental parameters, including the excitation wavelength, slit width, and the amount of the sample/substrate, were identical. The photoelectrochemical and electrochemical

analyses were carried out in a conventional three-electrode cell using a Pt plate and a Ag/AgCl electrode as the counter electrode and reference electrode, respectively. The experiments were conducted on a CHI760E workstation.The electrolyte was 25 mL MeCN/H_2O (v/v = 1:9) solution containing 1.0 M Na_2SO_3 for photocurrent and linear sweep voltammogram (LSV) test. For cyclic voltammograms (CV) text, 0.1 M tetrabutylammonium hexafluorophosphate/MeCN was employed as electrolyte. The working electrode was prepared on indium-tin-oxide (ITO) glass that was cleaned by sonication in ethanol for 30 min and dried at 80 °C. The boundary of ITO glass was protected using Scotch tape. 5 mg of the sample was dispersed in 0.5 mL DMF by sonication to get a slurry. The slurry (20 μL) was spread onto pre-treated ITO glass. After air drying, the working electrode was further dried at 100 °C for 2 h to improve adhesion. Then, the Scotch tape was removed and the uncoated part of the electrode was isolated with epoxy resin. The exposed area of the working electrode was 0.25 cm^2. The cathodic polarization curves were obtained using the linear sweep voltammetry technique with a scan rate of 0.1 mV s^{-1}. Before measurements, the electrolyte was pre-saturated with Ar by bubbling Ar into the electrolyte for 30 min. During measurements, Ar was continuously bubbled into the electrolyte at a flow rate of 10 mL min^{-1}.

6. Photoactivity testing. In a typical experiment, 5 mg photocatalysts were added to 2 mL solvent containing 0.1 mmol organic substrate and 2 mmol Na_2SO_3. The reaction suspension was sonicated for 2 min at room temperature and purged with Ar for 10 min. A white LED light ($\lambda > 420$ nm) was used as the irradiation source, which was placed at a distance of approximately 5 cm from the reactor to be the irradiation source to trigger the photocatalytic reaction. The energy output is 0.8 W cm^{-2}. During photocatalysis, the suspension was continuously stirred to ensure uniform irradiation. The evolved gases were analysed using a gas chromatograph (GC, SRI 8610C) equipped with a thermal conductivity detector (TCD). Products in solution were analyzed using an Agilent Gas Chromatograph (GC-7820) with a flame-ionization detector (FID) and identified by gas chromatography-mass spectrometry (GC-MS, Agilent Technologies, GC6890N, MS 5973). The ^{1}H NMR spectra were measured on a Bruker AVANCE NEO 400 MHz NMR. The yield and selectivity for the products were calculated using the following **Eqn. (1)** and **(2)**:

$$\text{Yield} = \frac{n_p}{n_0} \times 100\% \quad (1)$$

$$\text{Selectivity} = \frac{n_p}{n_p + n_{H_2}} \times 100\% \quad (2)$$

where n_0 is the initial amount of substrate; n_p and n_{H_2} stand for the amount of deuterated product and evolved H_2 at a certain time after the catalytic reaction, respectively. For the recycling test, the reaction mixture was centrifuged and the catalyst was washed with MeCN and reused for the next photocatalysis experiment. The recycling test was conducted under identical conditions to those of the photocatalytic reaction as mentioned above.

^{1}H NMR was used to quantify deuterium incorporation of products. The deuterium incorporation ratios (%) were calculated using **Eqn. (3)**:[3]

$$\% \text{ Deuteration} = 100 - \left[\left(\frac{\text{Residual integral}}{\text{Number of deuterated product}}\right) \times 100 \right] \quad (3)$$

7. DFT calculation details and methods: All DFT computations were performed using the Vienna Ab initio Simulation Package (VASP),[4] applying the generalized gradient approximation (GGA) parametrized by Perdew, Burke and Ernzerhof (PBE)[5] and using the projector-augmented wave (PAW) pseudopotentials.[6] The +U correction was further added

on top of GGA to take into account the effect of on-site Coulomb interactions arising from Zn and In atoms.[7] Based on benchmarking done in the past, U values of 4.5 eV and 5 eV respectively were used for Zn and In.[8] The plane wave energy cut-off was set at 500 eV and all atomic structures were fully relaxed until forces on all atoms were less than 0.05 eV/Å. Brillouin zone integration was performed using Gamma-point only on a 6x6x1 supercell of ZIS. Point defects were simulated by removing atom(s) from the supercell to create vacancies and adding atoms in the supercell to create interstitials. For every defect site, the lowest energy ones are reported here. The defect formation energy (E^{form}) is calculated as:

$$E^{form} = E(D\text{-}ZIS) - E(ZIS) + \mu$$

Here, the first two terms are the total DFT energies of the defect containing and bulk supercells, respectively, and μ is the chemical potential of the species involved in creating the defect. Constraints were placed on all chemical potentials, namely μ_S, μ_{Zn} and μ_{In}, so as to ensure thermodynamic equilibrium conditions for $ZnIn_2S_4$ and avoid the likelihood of decomposition to ZnS and In_2S_3. These conditions can be written as:

$$\Delta\mu_{Zn} + 2*\Delta\mu_{In} + 4*\Delta\mu_S = \Delta H(ZnIn_2S_4)$$

$$\Delta\mu_{Zn} + \Delta\mu_S < \Delta H(ZnS)$$

$$2*\Delta\mu_{In} + 3*\Delta\mu_S < \Delta H(In_2S_3)$$

Here, $\Delta H(ZnIn_2S_4) = E(ZnIn_2S_4) - E(Zn) - 2*E(In) - 4*E(S)$, $\Delta H(ZnS) = E(ZnS) - E(Zn) - E(S)$, and $\Delta H(In_2S_3) = E(In_2S_3) - 2*E(In) - 3*E(S)$. E(system) refers to the total DFT energy of the corresponding system. The chemical potentials of Zn, In and S are referenced to their respective elemental standard states, such that $\mu_{Zn} = \Delta\mu_{Zn} + E(Zn)$, $\mu_{In} = \Delta\mu_{In} + E(In)$, and $\mu_S = \Delta\mu_S + E(S)$. Based on the above constraints, we selected three chemical potential conditions (μ_1, μ_2 and μ_3) with decreasing (increasing) values of μ_{Zn} (μ_S), and reported the defect formation energies for each.

The surface slab calculations for H* adsorption were performed using a 1x2 supercell with an exposed (110) surface. The smaller system size and a lower kinetic energy cutoff of 400 eV were preferred to reduce computational costs, and all other DFT parameters were kept the same. The H adsorption energy is calculated as:

$$E_{ads} = E(H{:}ZIS) - E(ZIS) - ½*E(H_2)$$

Here, E(ZIS) is the DFT energy of the bulk or defect containing ZIS slab structure, E(H:ZIS) is the DFT energy of the same slab structure with H- adsorption, and $E(H_2)$ is the DFT energy of an H_2 molecule. More negative E_{ads} values imply more favorable H adsorption.

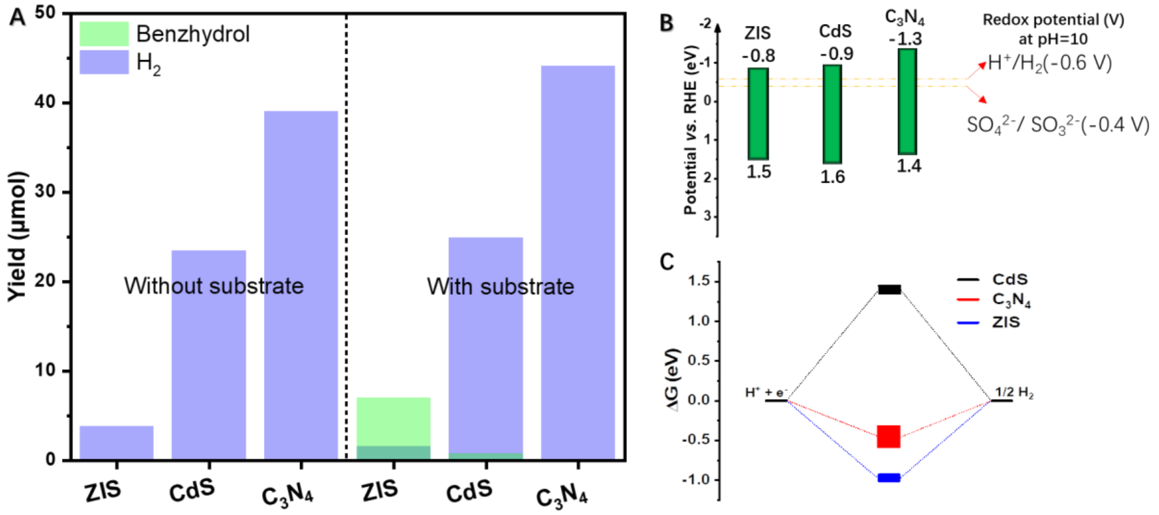

Figure S1. (A) Benzhydrol and H_2 production under visible light (λ > 420 nm) irradiation of 24 h over ZIS, CdS and C_3N_4 semiconductors. (B) Band alignment of ZIS, CdS and C_3N_4 and redox potential of involved redox half-reactions in this work. (C) Free energy change (ΔG) for HER over ZIS, CdS and C_3N_4. Data in (B) and (C) are cited from ref.[1] and ref.[2], respectively.

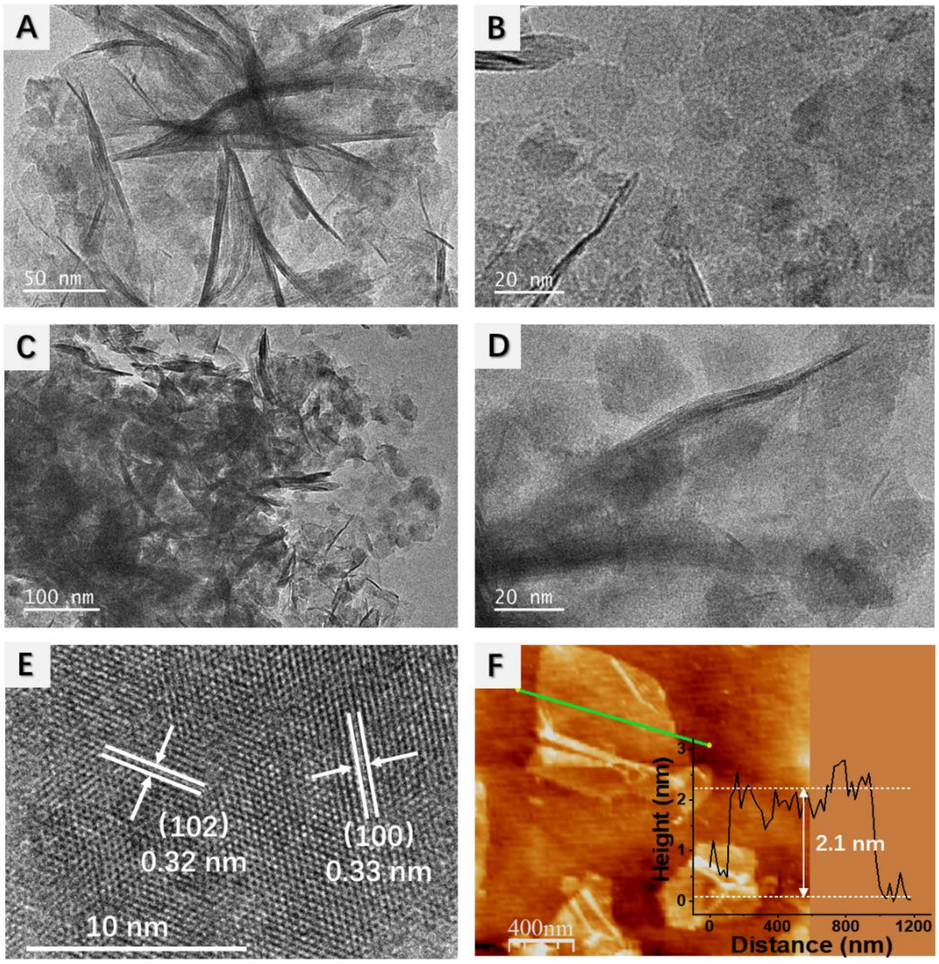

Figure S2. TEM images of (A, B) D-ZIS and (C,D) blank ZIS nanosheets. (E) HR-TEM and (F) AFM images of blank ZIS nanosheets. Inset of (F) shows the height profile.

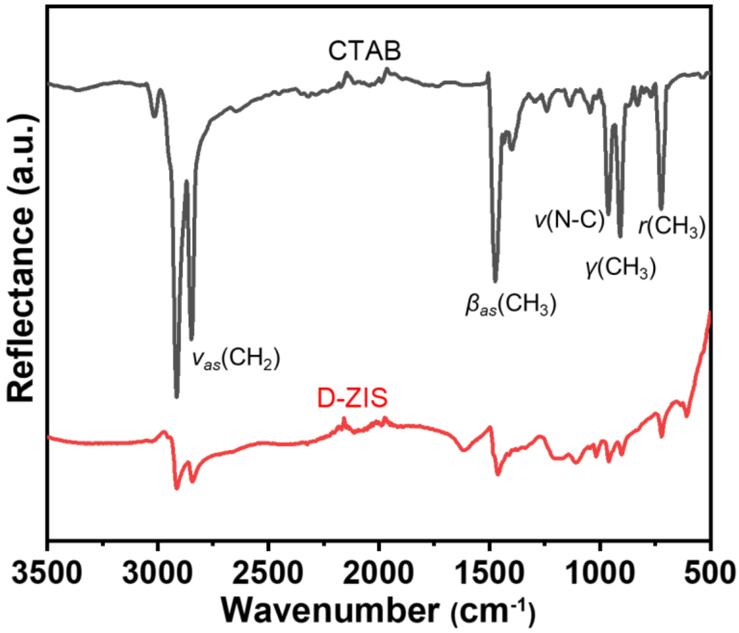

Figure S3. IR spectrum of CTAB and D-ZIS.

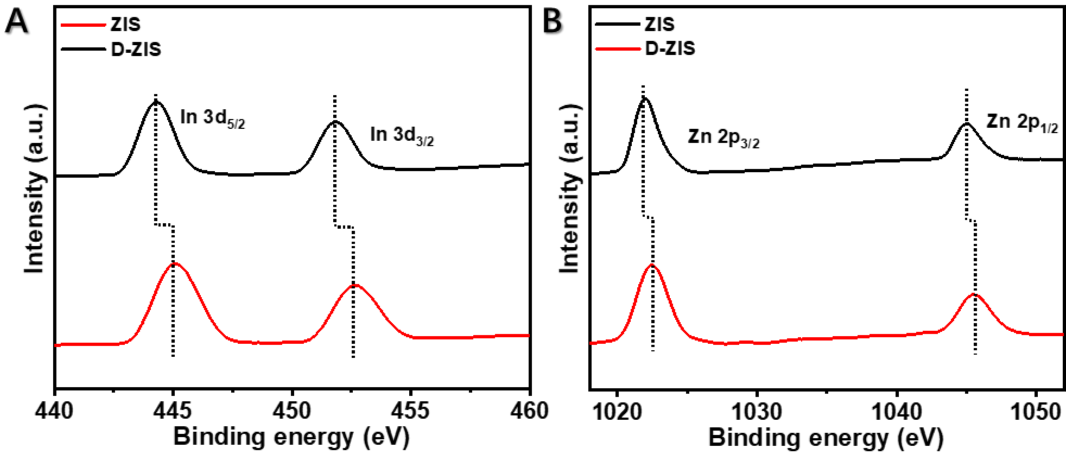

Figure S4. XPS spectra of (A) In 3d and (B) Zn 2p in D-ZIS and ZIS.

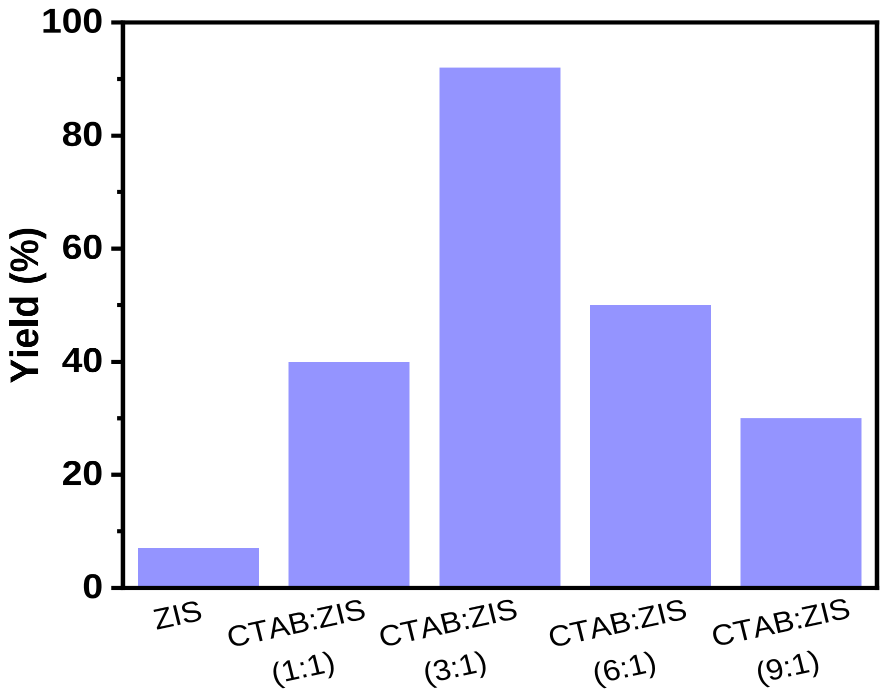

Figure S5. Yields of benzhydrol over D-ZIS samples with different weight ratios of CTAB vs. ZIS in catalyst preparation. Reaction condition: 0.1 mmol substrate, 5 mg catalyst, 0.2 mL MeCN, 1.8 mL D_2O, 2 mmol Na_2SO_3, irradiation with visible light ($\lambda > 420$ nm) of 24 h.

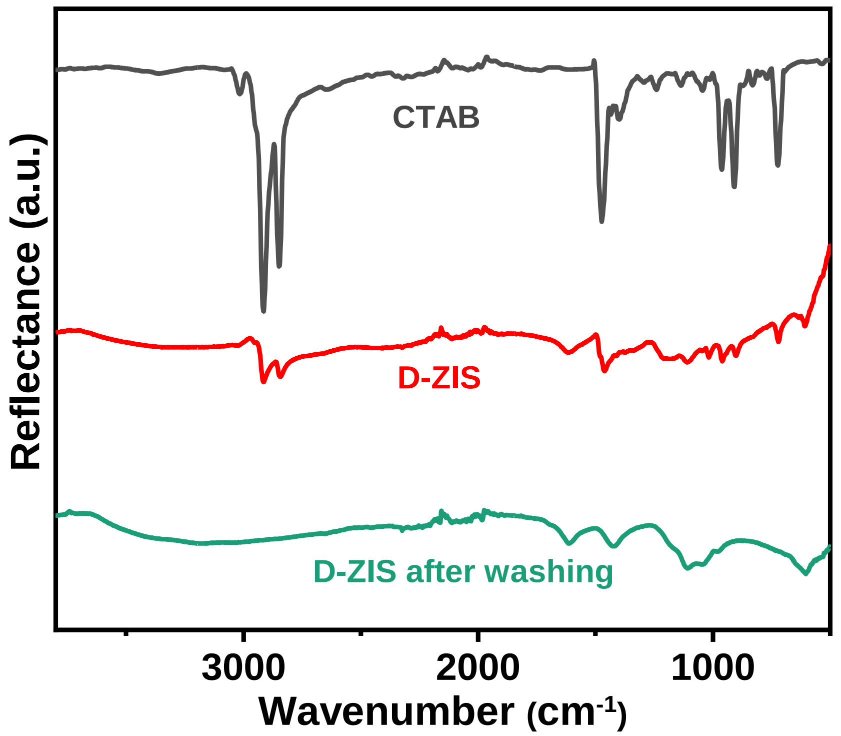

Figure S6. IR spectra of CTAB, D-ZIS and D-ZIS after washing.

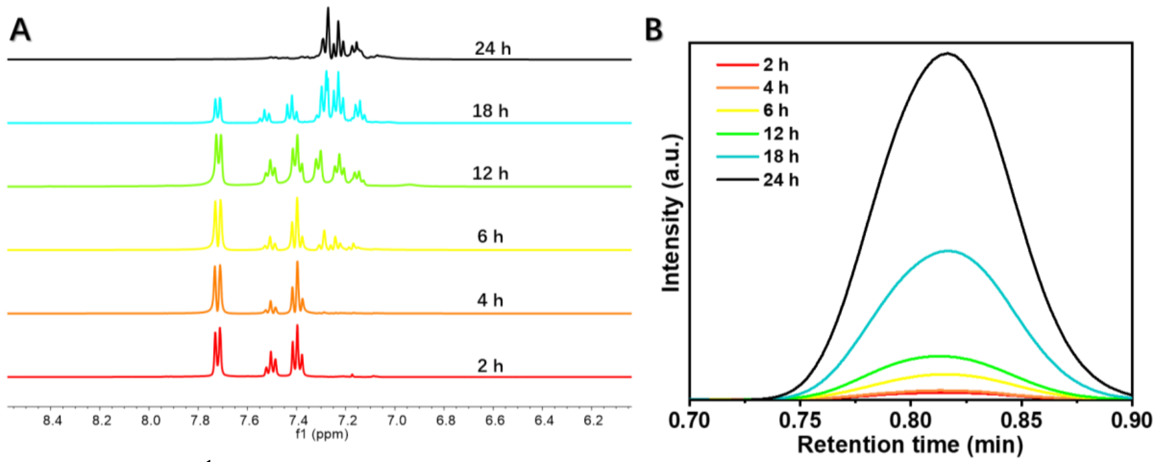

Figure S7. (A) ^{1}H NMR spectra and (B) GC signal of D_2 showing the products evolution during the deuteration reaction with different times.

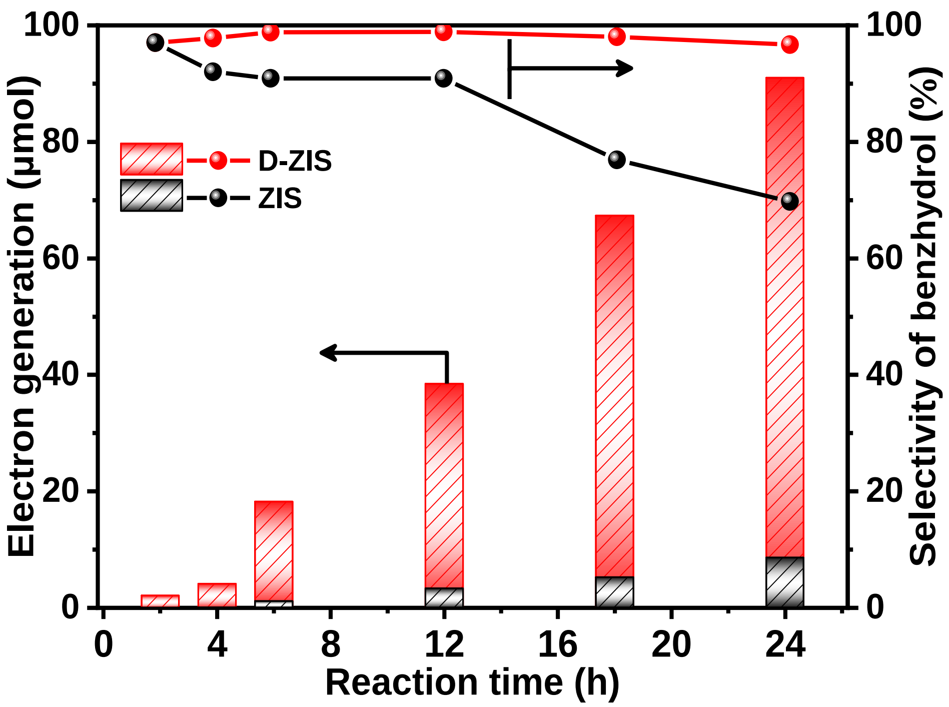

Figure S8. Total electron generation and the electron consumption percentage (i.e., selectivity) toward benzhydrol production on ZIS and D-ZIS.

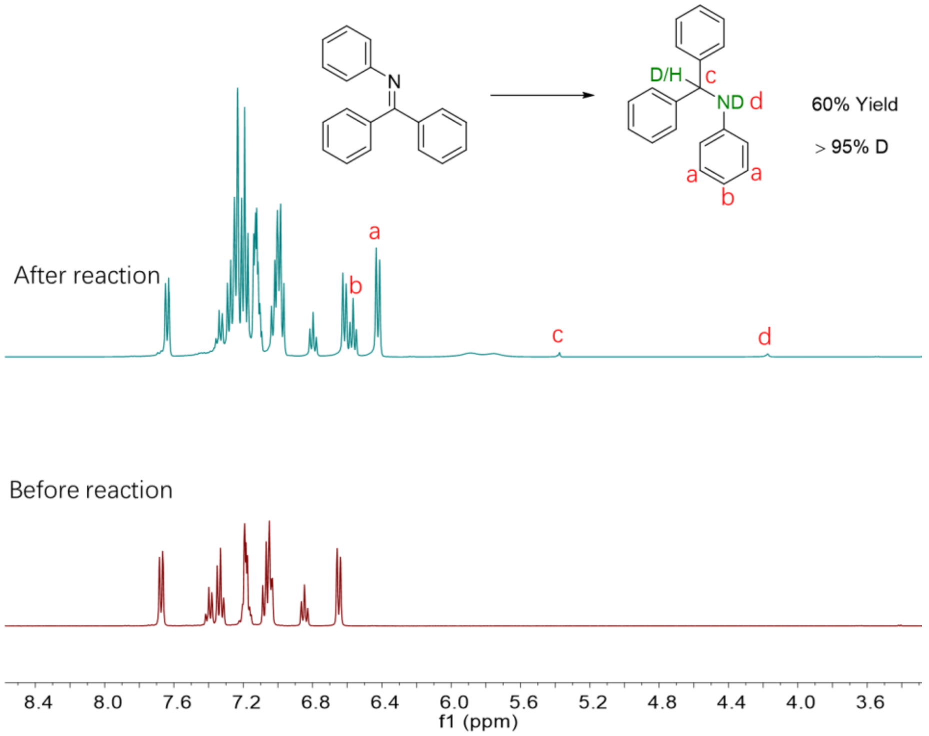

Figure S9. ^{1}H NMR spectra showing the products during the photocatalytic deuteration of benzophenone phenylimine.

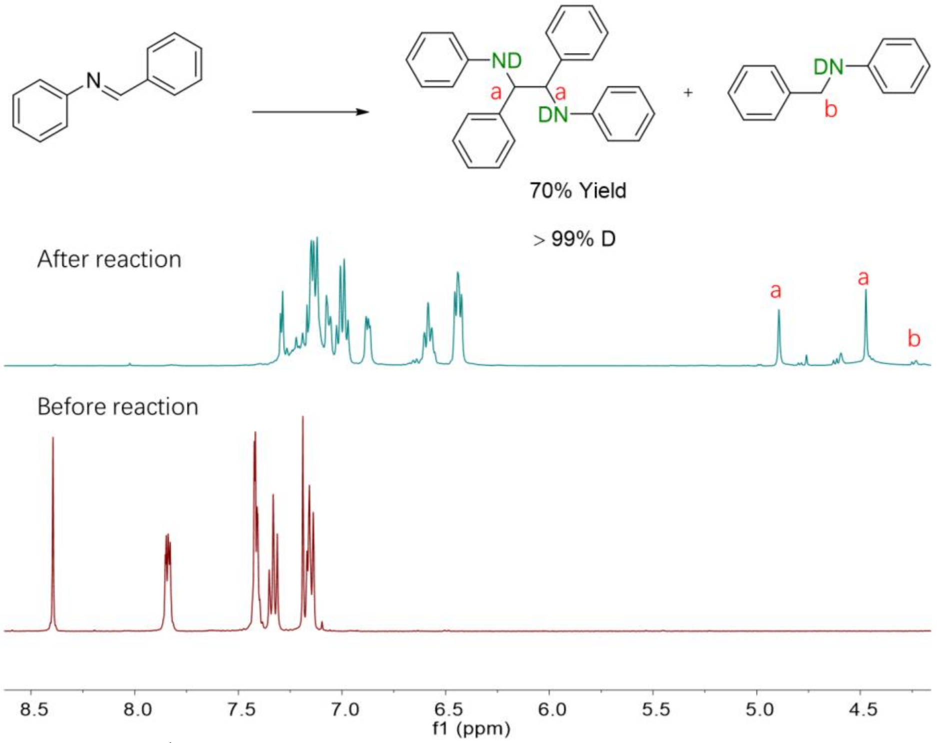

Figure S10. ^{1}H NMR spectra showing the products during the photocatalytic deuteration of diphenylimine.

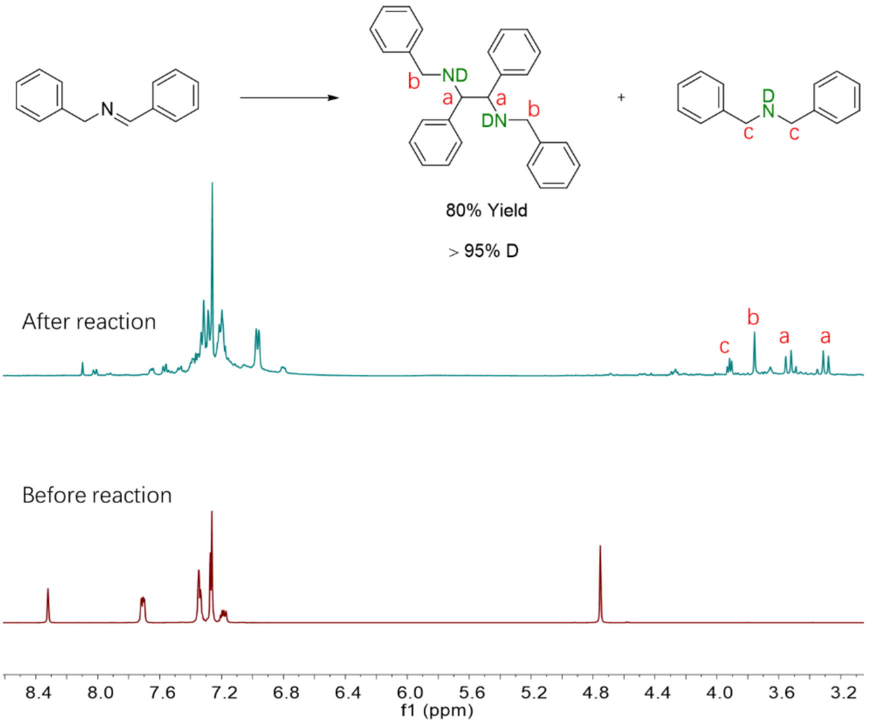

Figure S11. ^{1}H NMR spectra showing the products during the photocatalytic deuteration of N-benzylidenebenzylamine.

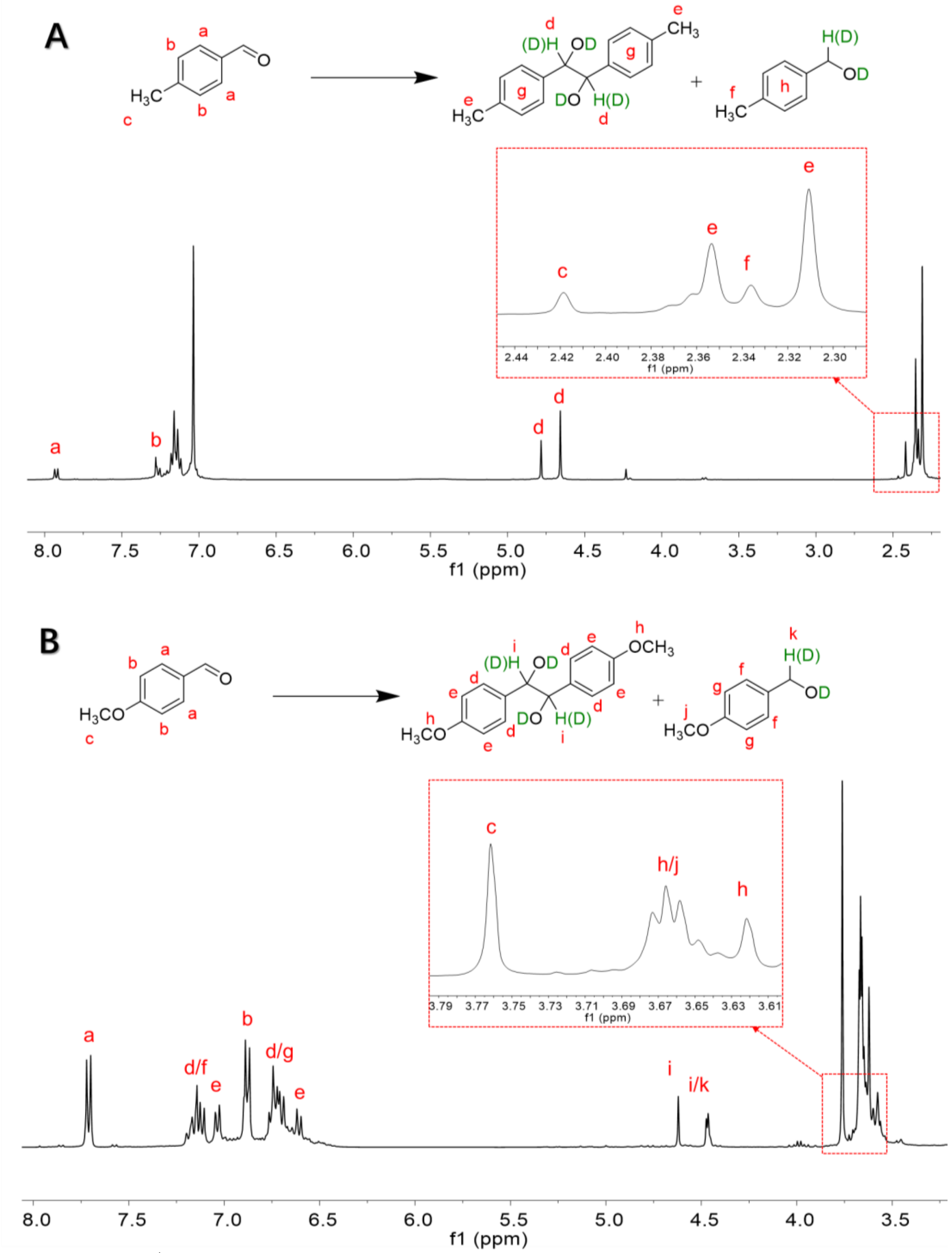

Figure S12. ^{1}H NMR spectra showing the products during the photocatalytic deuteration of (A) 4-methyl benzaldehyde and (B) 4-methoxybenzaldehyde.

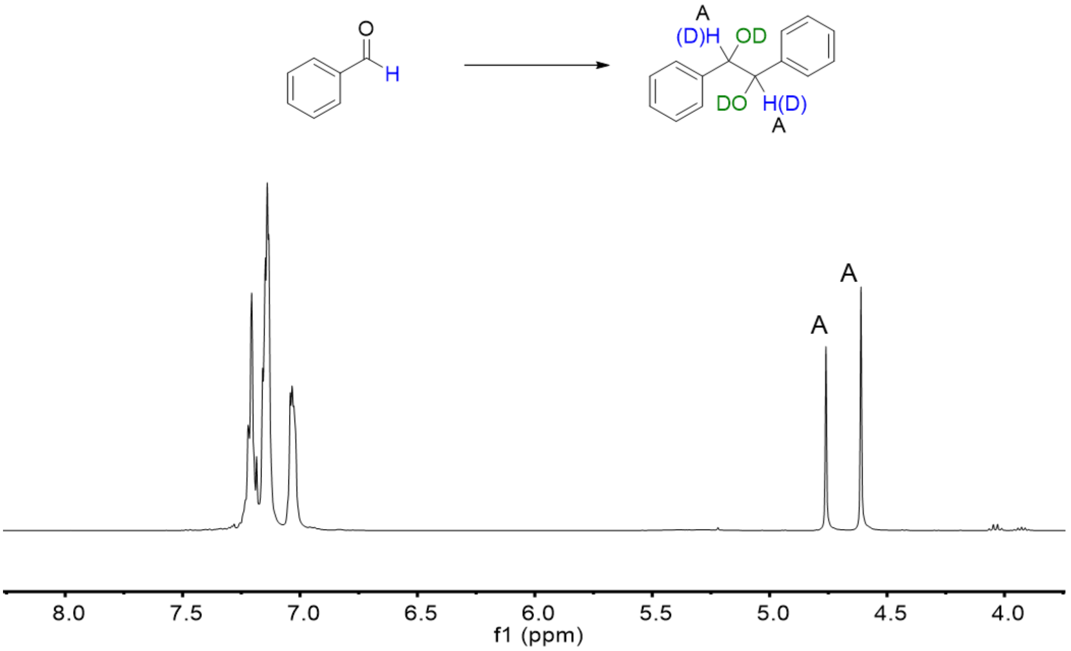

Figure S13. ^{1}H NMR spectra showing the product of hydrobenzoin during the photocatalytic deuteration of benzaldehyde.

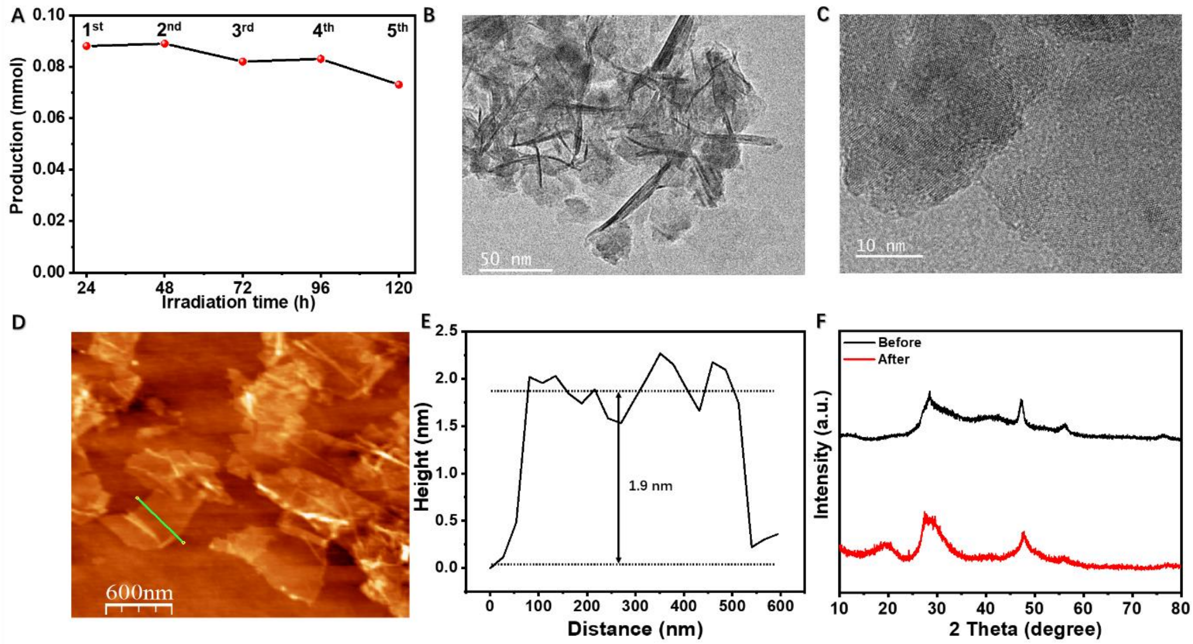

Figure S14. (A) Recycling test of D-ZIS. Reaction condition: 0.1 mmol benzophenone, 5 mg D-ZIS, 0.2 mL MeCN, 1.8 mL D_2O, 2 mmol Na_2SO_3, irradiation of 24 h with visible light ($\lambda >$ 420 nm) in each round. (B and C) TEM images, (D) AFM image, (E) corresponding height profile and (F) XRD of D-ZIS after five consecutive cycles of benzophenone deuteration. For comparison, the XRD of D-ZIS before the reaction is also included in (F).

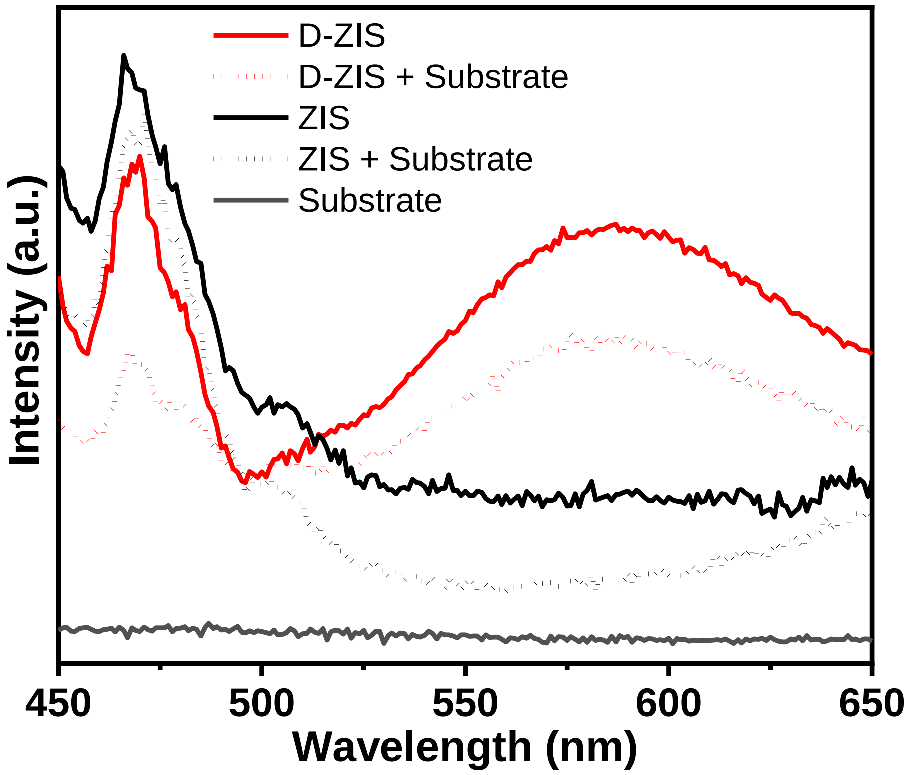

Figure S15. Photoluminescence spectra of different samples with or without benzophenone upon excitation at 340 nm.

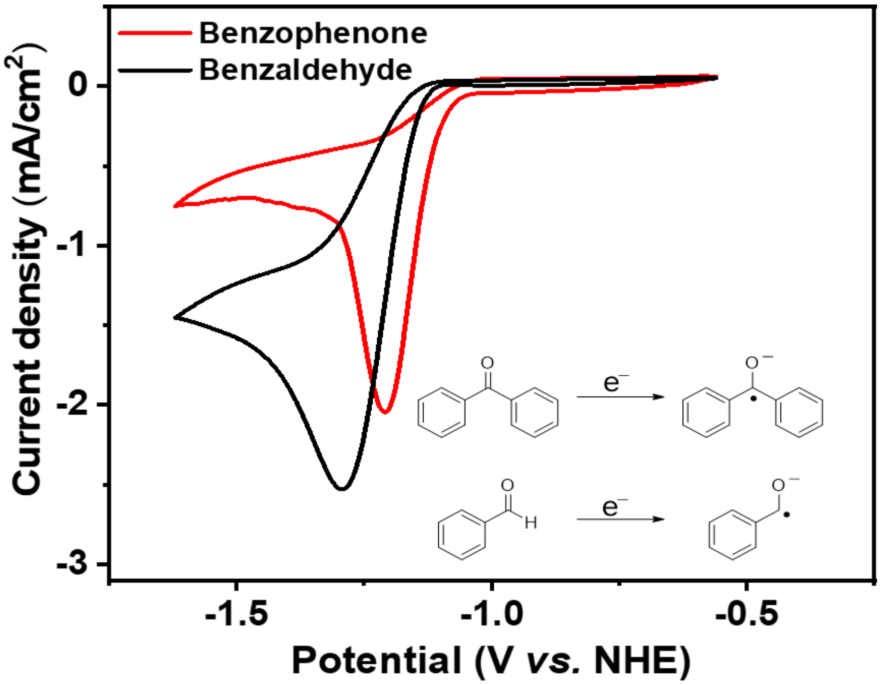

Figure S16. Cyclic voltammograms of 10 mM benzophenone and 10 mM benzaldehyde.

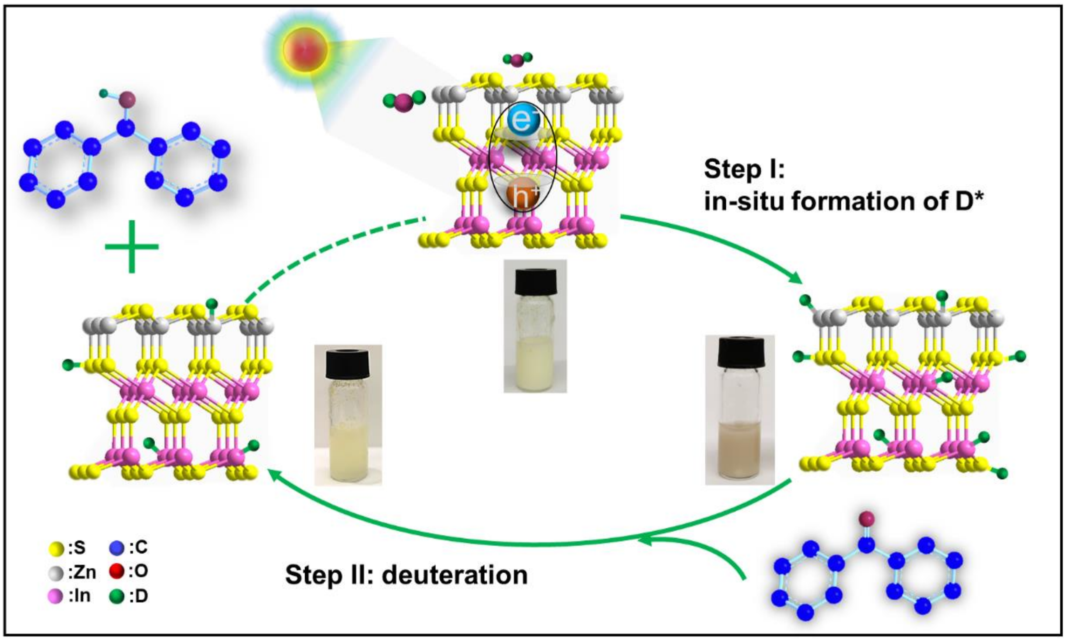

Figure S17. Schematic and photographs showing the stepwise deuteration of benzophenone with D_2O under controlled conditions.

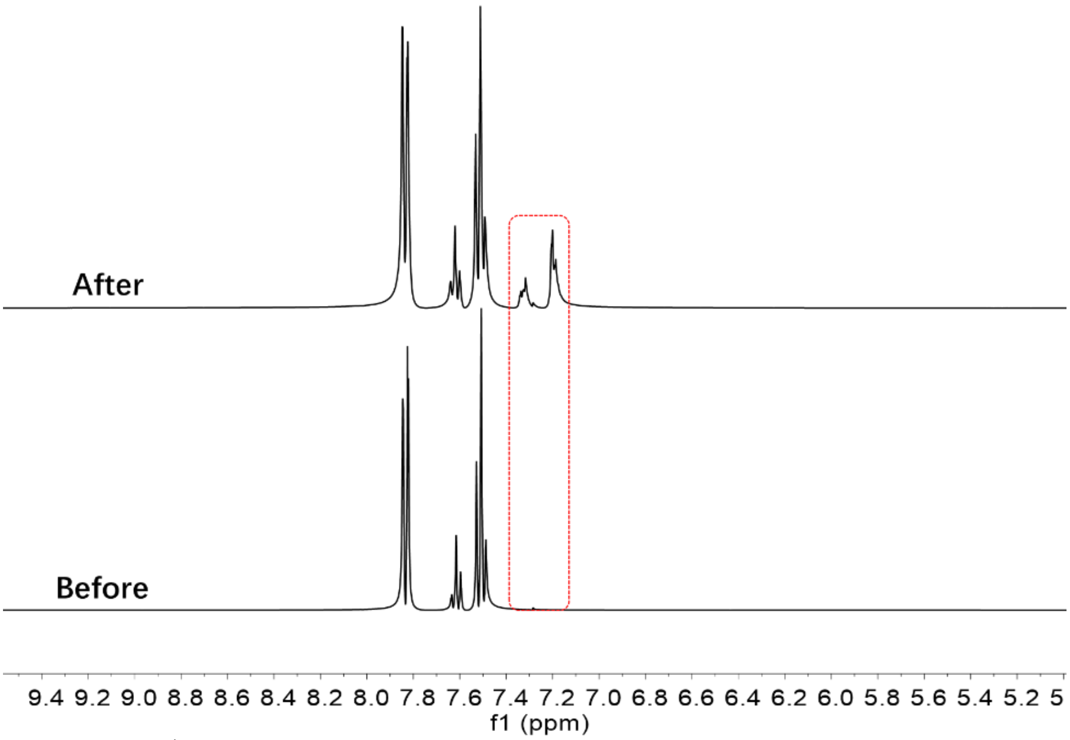

Figure S18. ^{1}H NMR spectrum of the crude reaction mixture after reaction of 24 h under dark. For comparison, the 1H NMR spectrum of benzophenone before the reaction is also included.

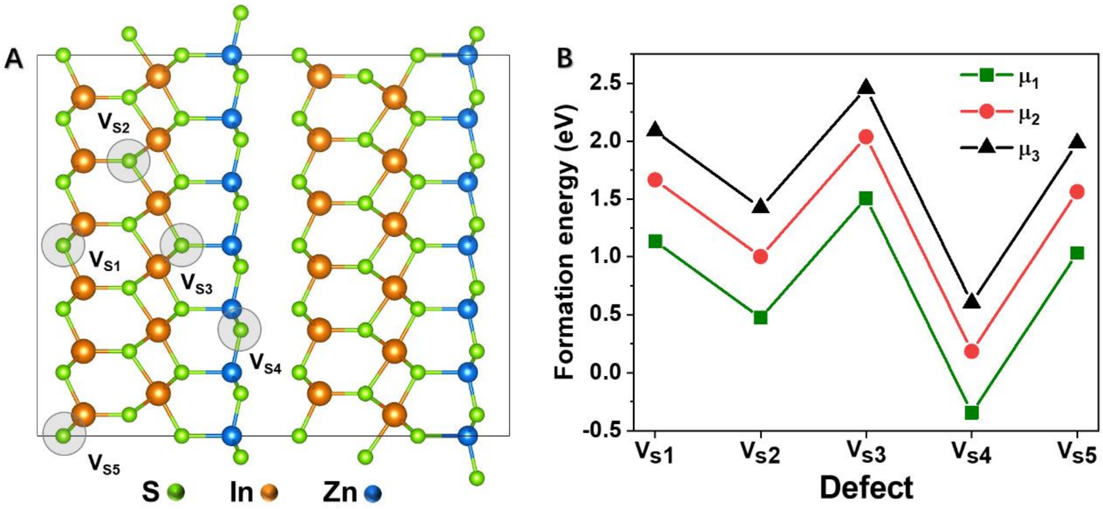

Figure S19. (A) The optimized structure of hexagonal $ZnIn_2S_4$ with different S vacancies employed in DFT computations. (B) Defect formation energies corresponding to various vacancies in panel (A).

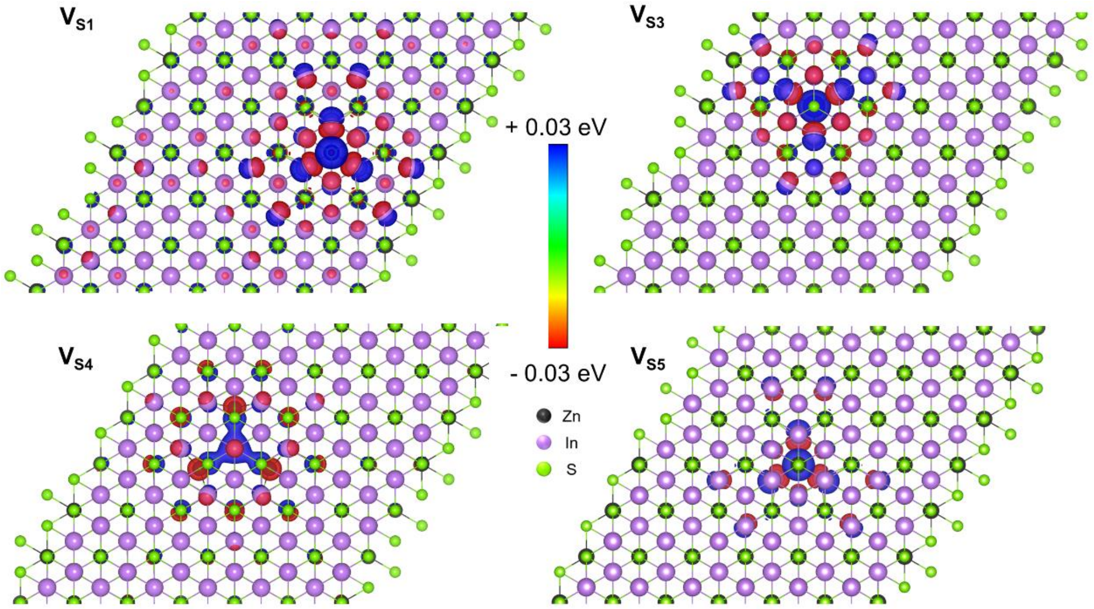

Figure S20. The electron density distribution around D-ZIS with different defect states corresponding to **Figure S19A**.

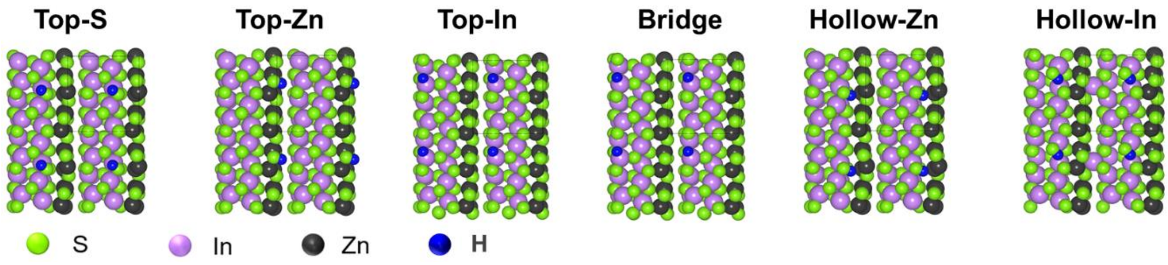

Figure S21. The models of H* adsorption on different sites of ZIS (110) surface.

Table S1. Elemental analyses of ZIS and D-ZIS.

Sample	Atomic ratio of Zn/In/S by XPS (ICP)	S vacancy density by XPS (ICP)[a)]
ZIS	1/2/3.8 (1/2.1/3.8)	5% (5%)
D-ZIS	1/2.1/3.1 (1/2.1/3)	23% (25%)

[a)]Determined by the percentage ratio between missing S atoms in D-ZIS and theoretically total S atoms in ZIS.

Table S2. Yields of benzhydrol obtained under different reaction conditions.

Entry	Change from standard condition[a)]	Yield %
1	None	92
2	0.5 mmol Na_2SO_3	42
3	1 mmol Na_2SO_3	71
4	With 2 mL 3/1 (v/v) of MeCN/H_2O	41
5	With 2 mL 1/1 (v/v) of MeCN/H_2O	51
6	With 2 mL H_2O	28
7	With 2 mL 1/9 (v/v) of MeCN/D_2O	88
8	With CTAB+ZIS instead of D-ZIS	12
9	With D-ZIS after removing CTAB	82
10	Without light or D-ZIS	0

[a)]Standard condition: 0.1 mmol substrate, 5 mg catalyst, 0.2 mL MeCN, 1.8 mL H_2O, 2 mmol Na_2SO_3, irradiation with visible light ($\lambda > 420$ nm) for 24 h.

Table S3. BET surface areas and elemental analyses of fresh D-ZIS and used D-ZIS after recycling test.

Sample	BET surface areas (m^2/g)	S vacancy density by ICP
Fresh D-ZIS	81.3	25%
Used D-ZIS	70.2	32%

Supplemented NMR spectra

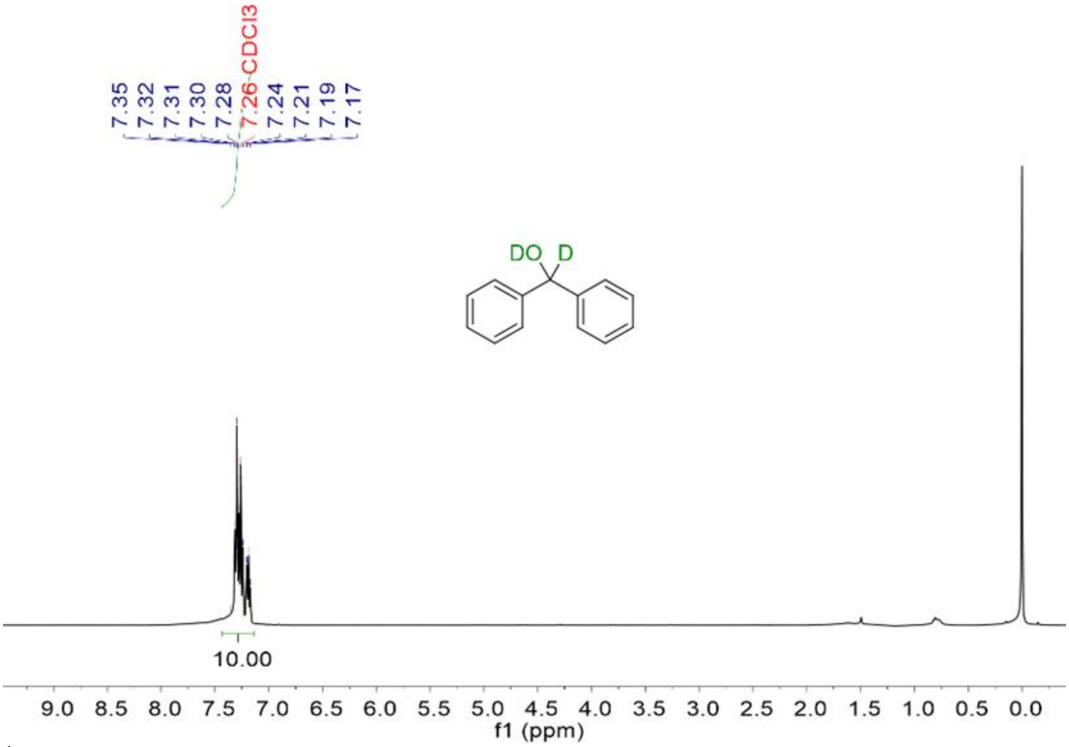

^{1}H NMR spectrum of **1** (400 MHz, $CDCl_3$).

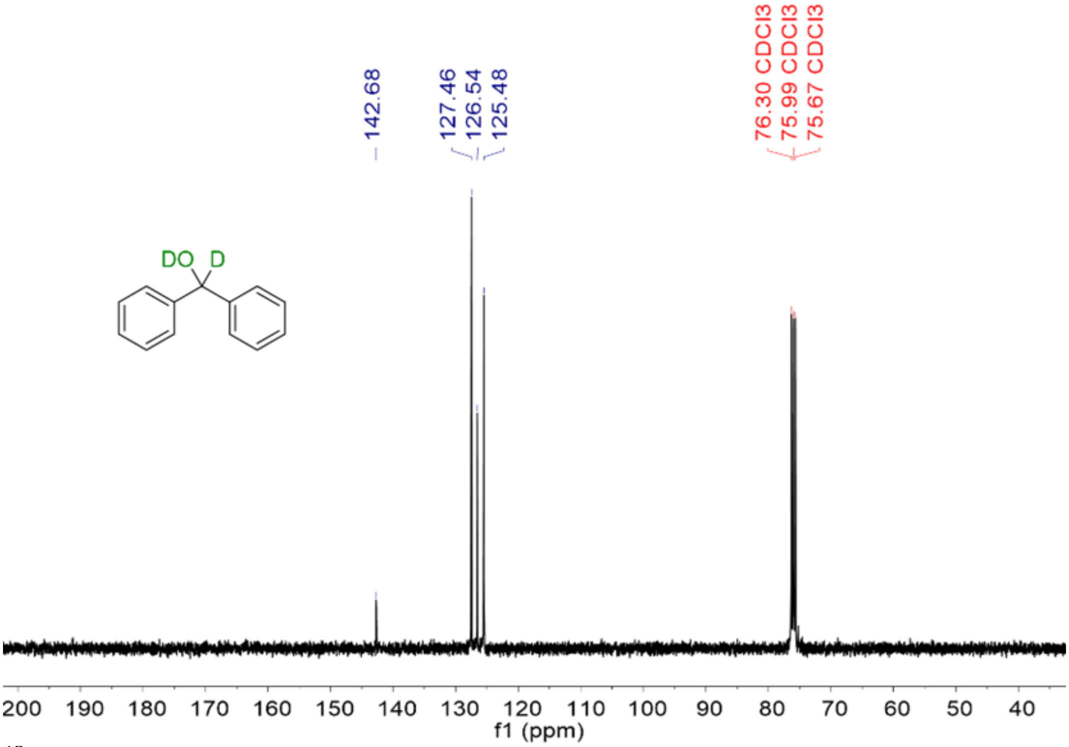

^{13}C NMR spectrum of **1** (100 MHz, $CDCl_3$).

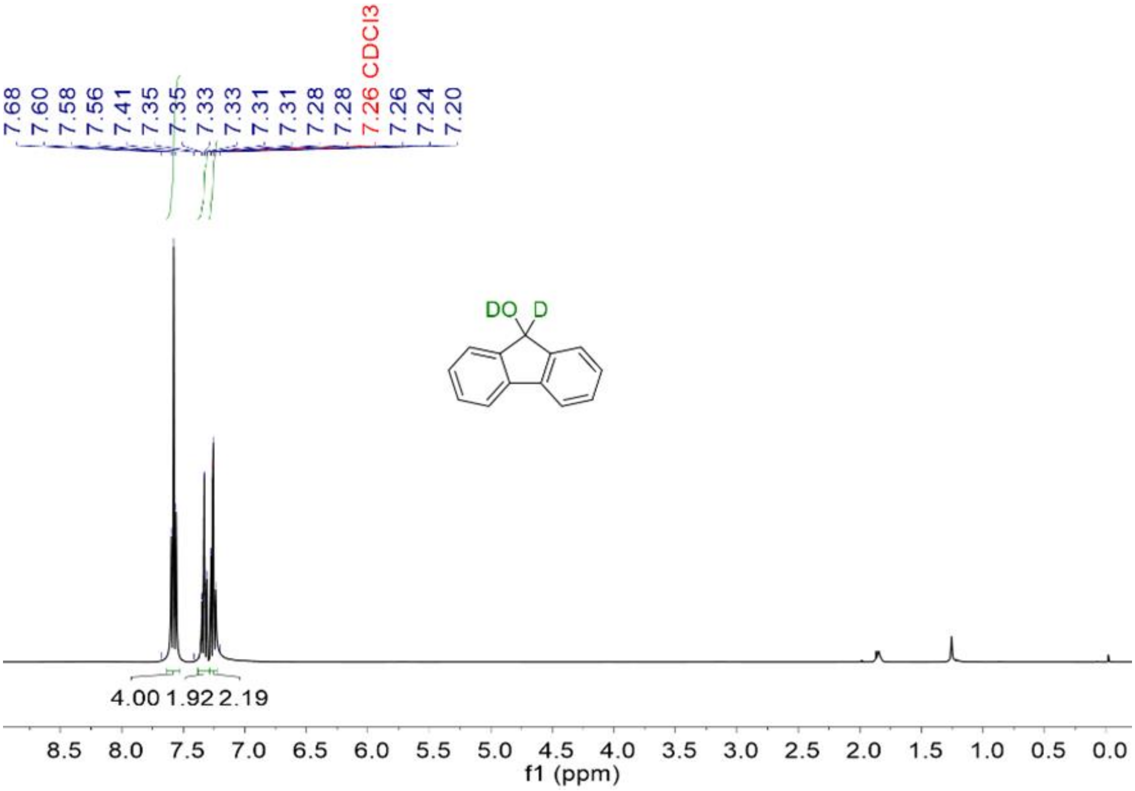

^{1}H NMR spectrum of **2** (400 MHz, $CDCl_3$).

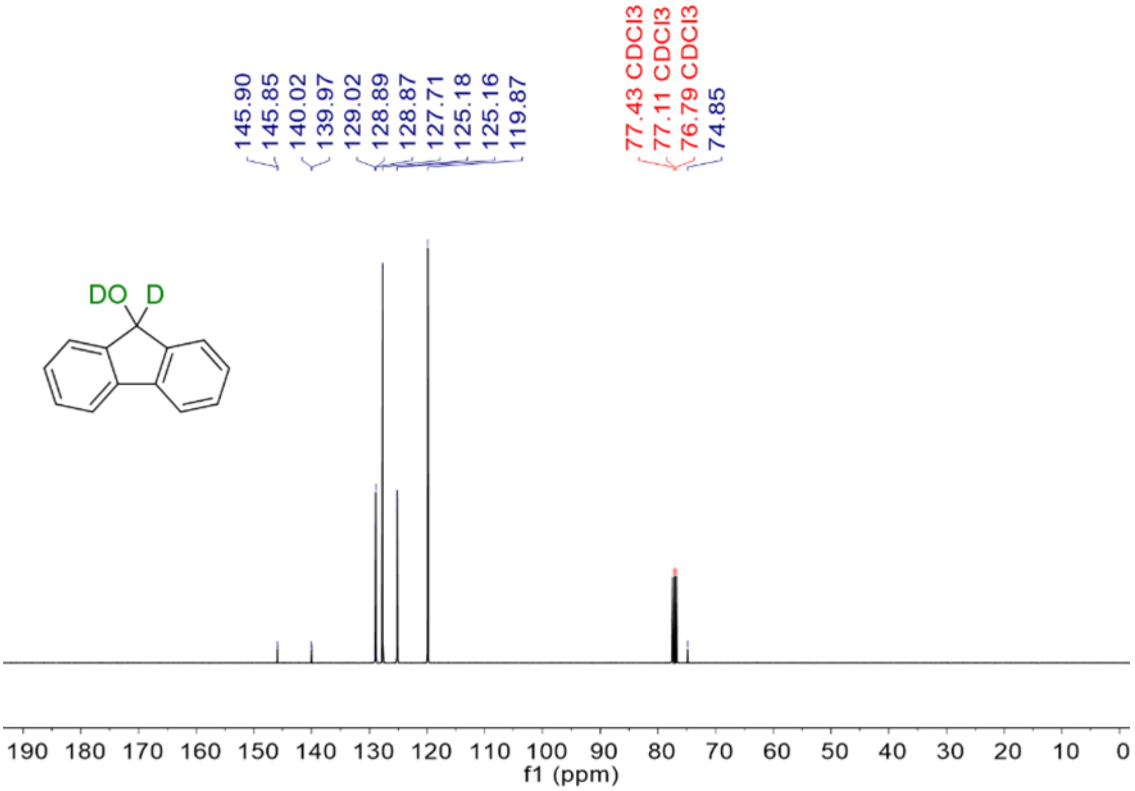

^{13}C NMR spectrum of **2** (100 MHz, $CDCl_3$).

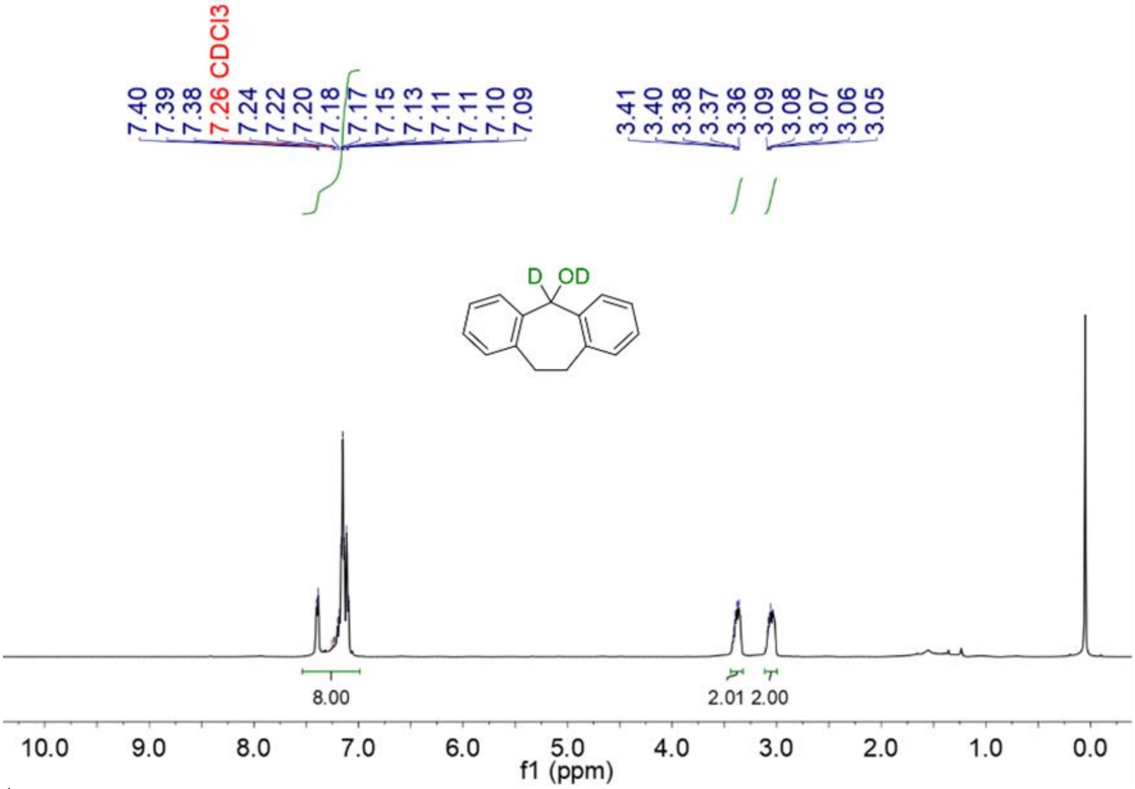

^{1}H NMR spectrum of **3** (400 MHz, $CDCl_3$).

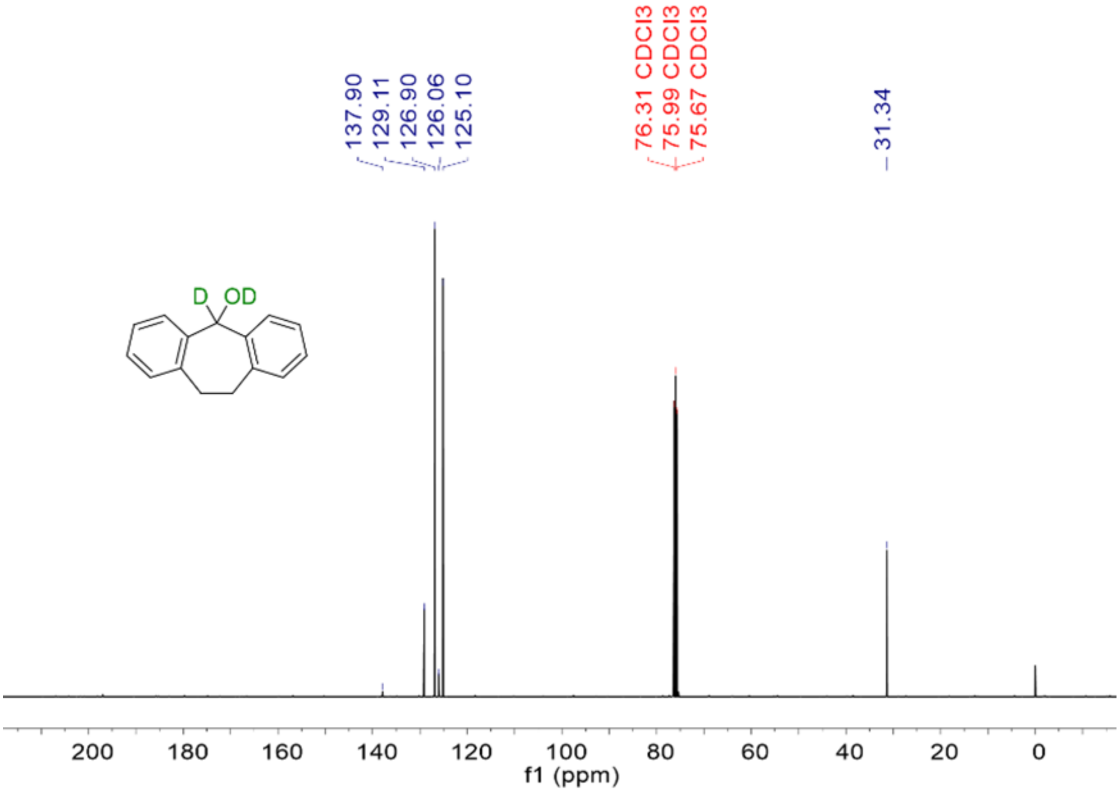

^{13}C NMR spectrum of **3** (100 MHz, $CDCl_3$).

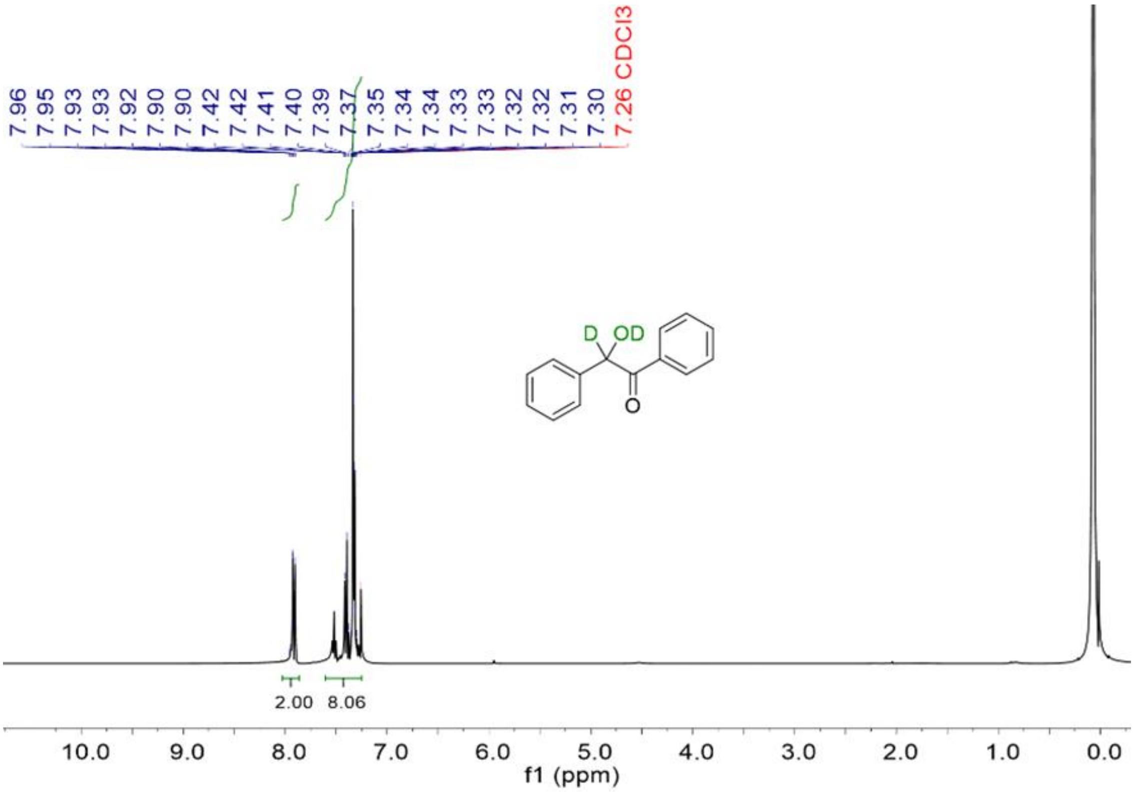

^{1}H NMR spectrum of **5** (400 MHz, $CDCl_3$).

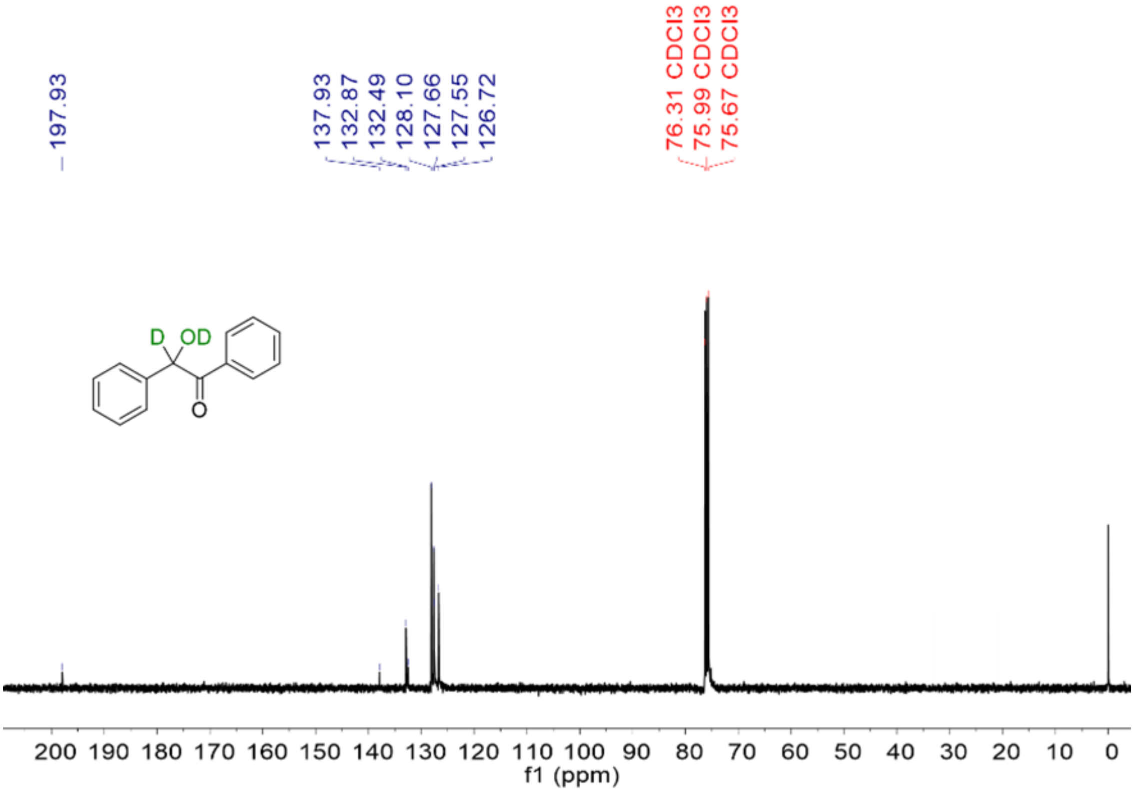

^{13}C NMR spectrum of **5** (100 MHz, $CDCl_3$).

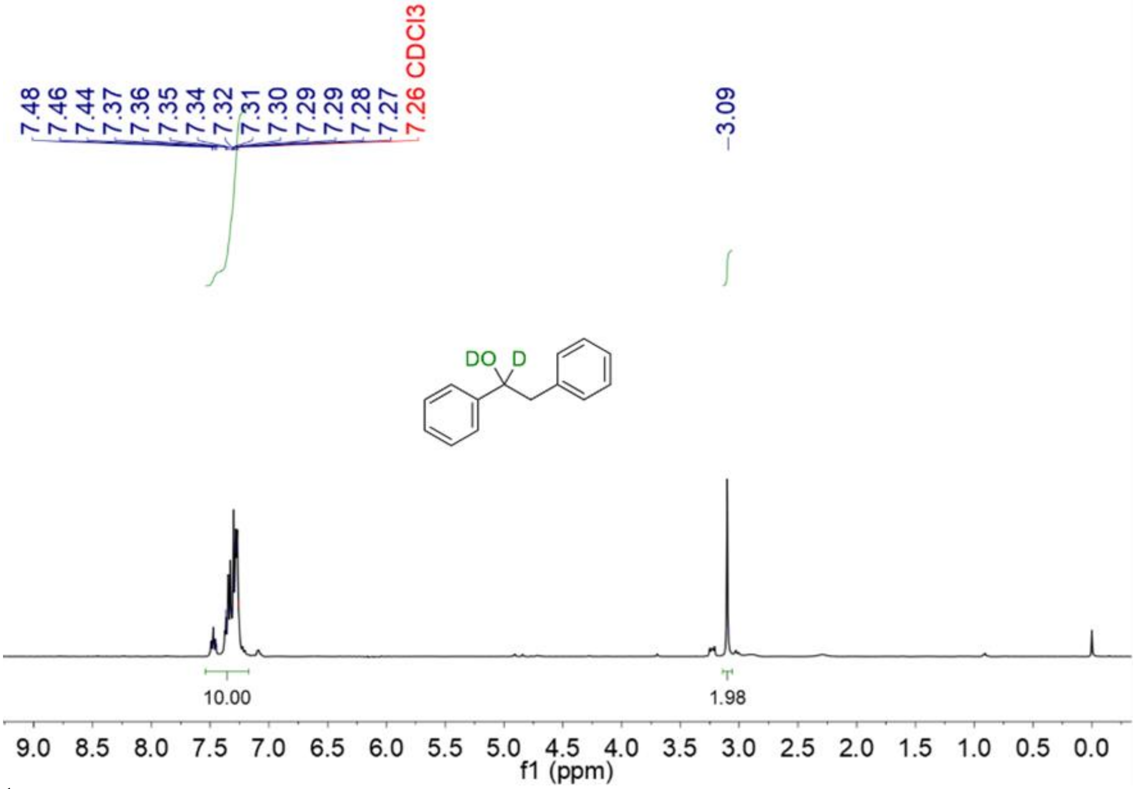

^{1}H NMR spectrum of **6** (400 MHz, $CDCl_3$).

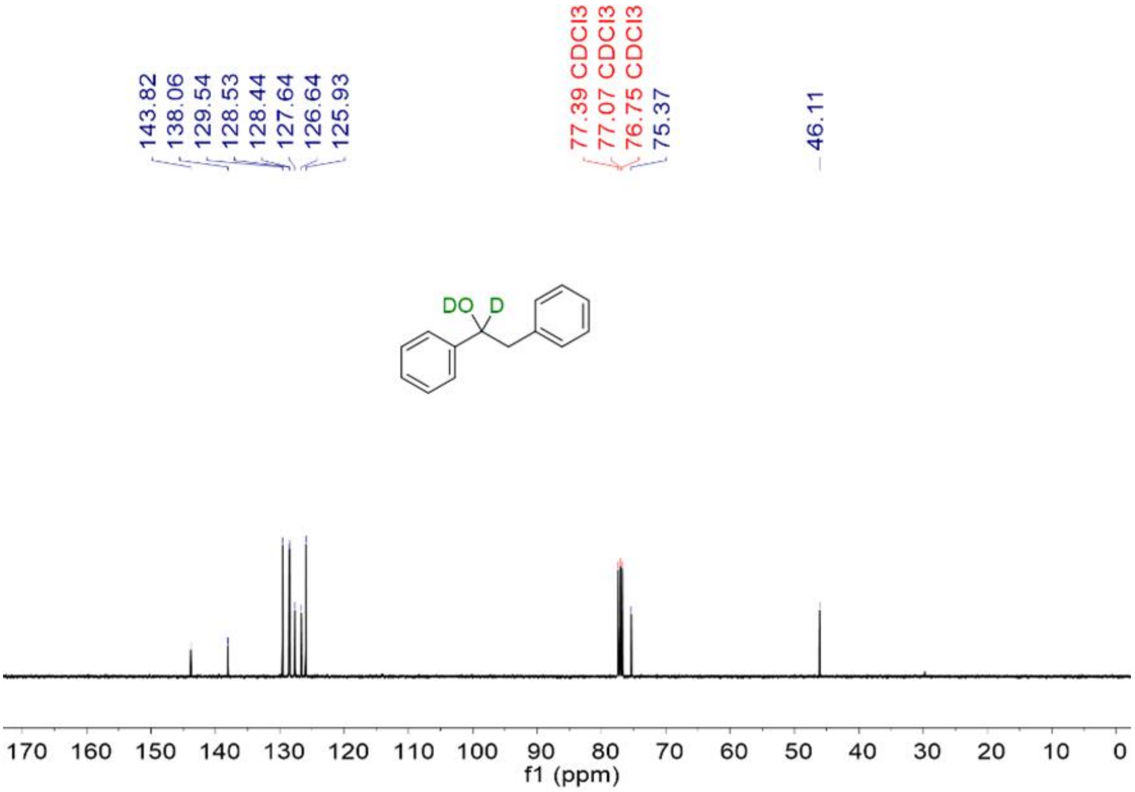

^{13}C NMR spectrum of **6** (100 MHz, $CDCl_3$).

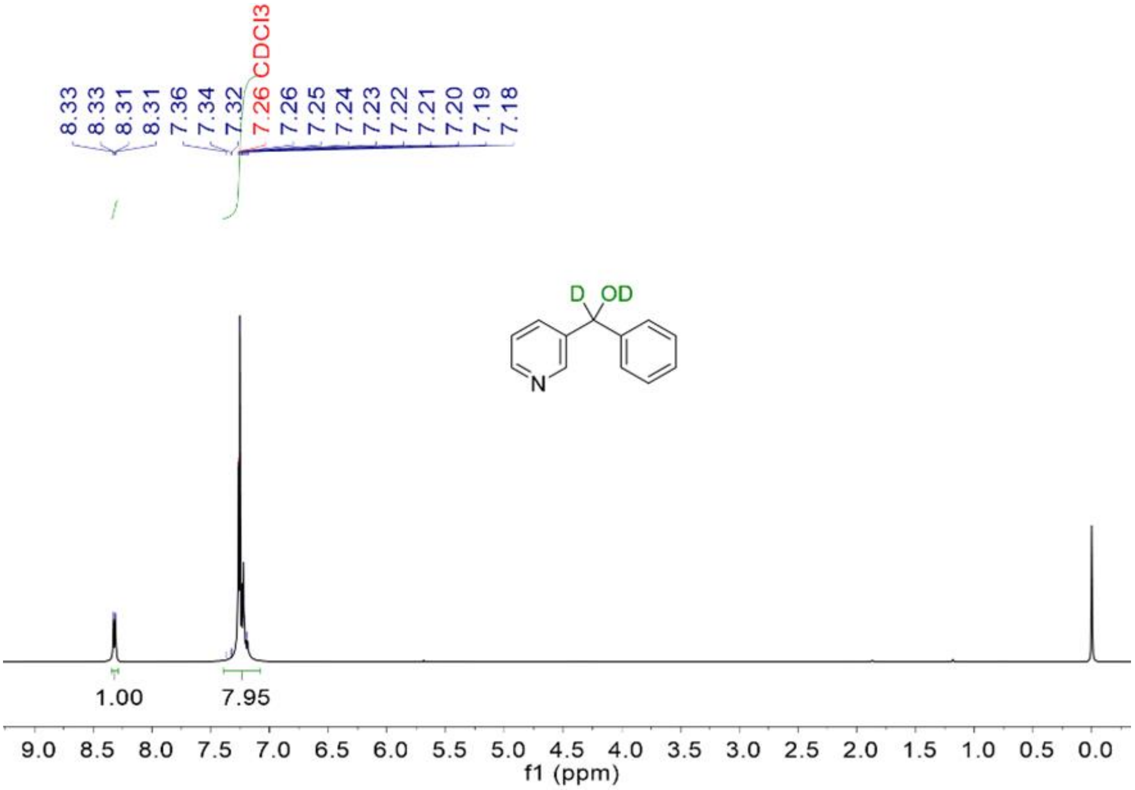

^{1}H NMR spectrum of **7** (400 MHz, $CDCl_3$).

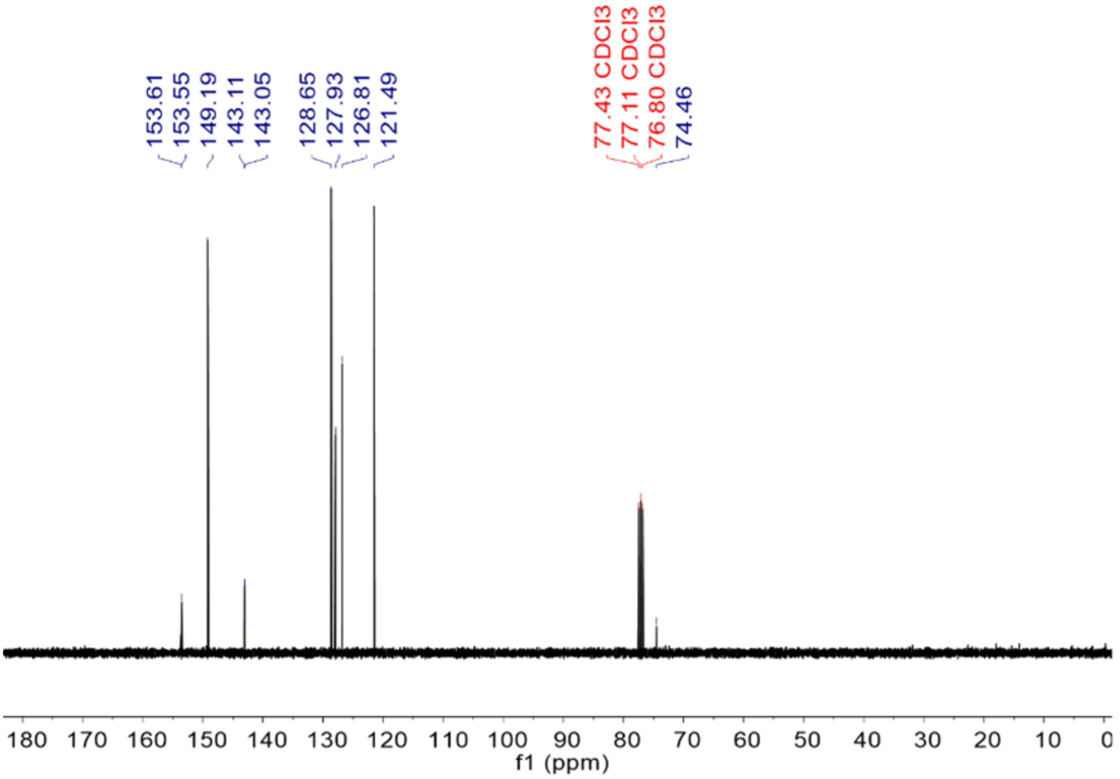

^{13}C NMR spectrum of **7** (400 MHz, $1DCl_3$).

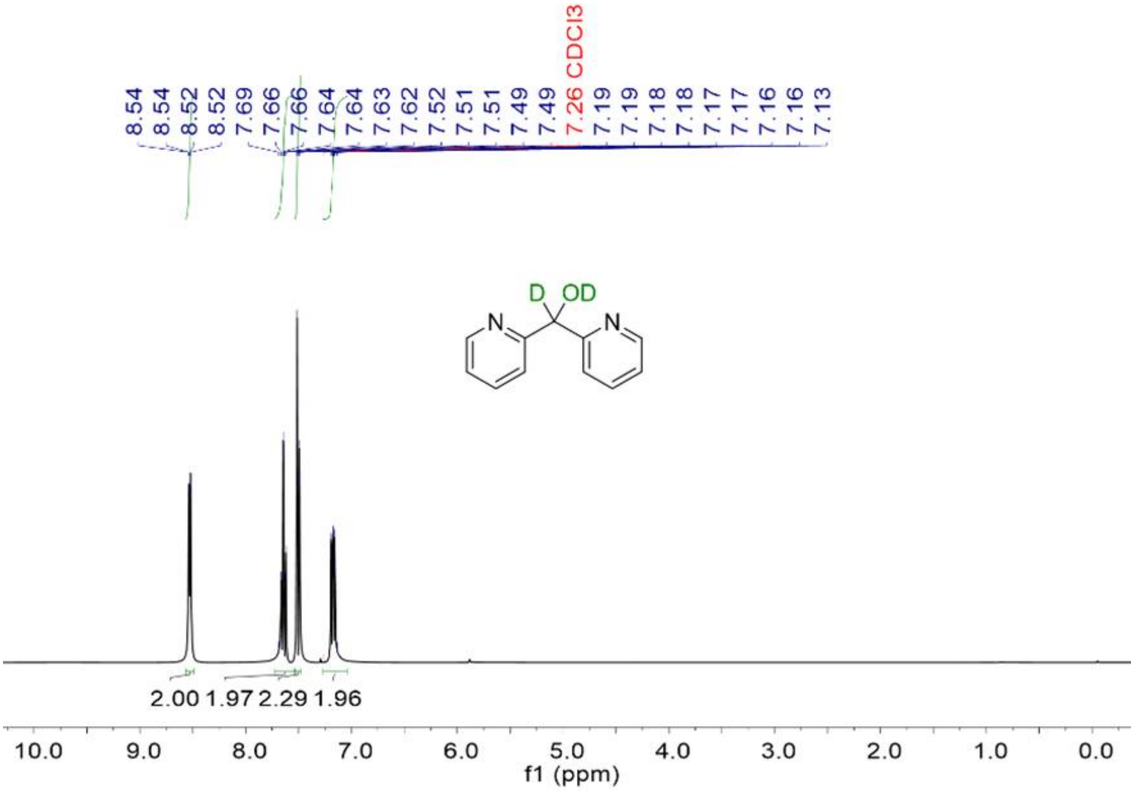

^{1}H NMR spectrum of **8** (400 MHz, $CDCl_3$).

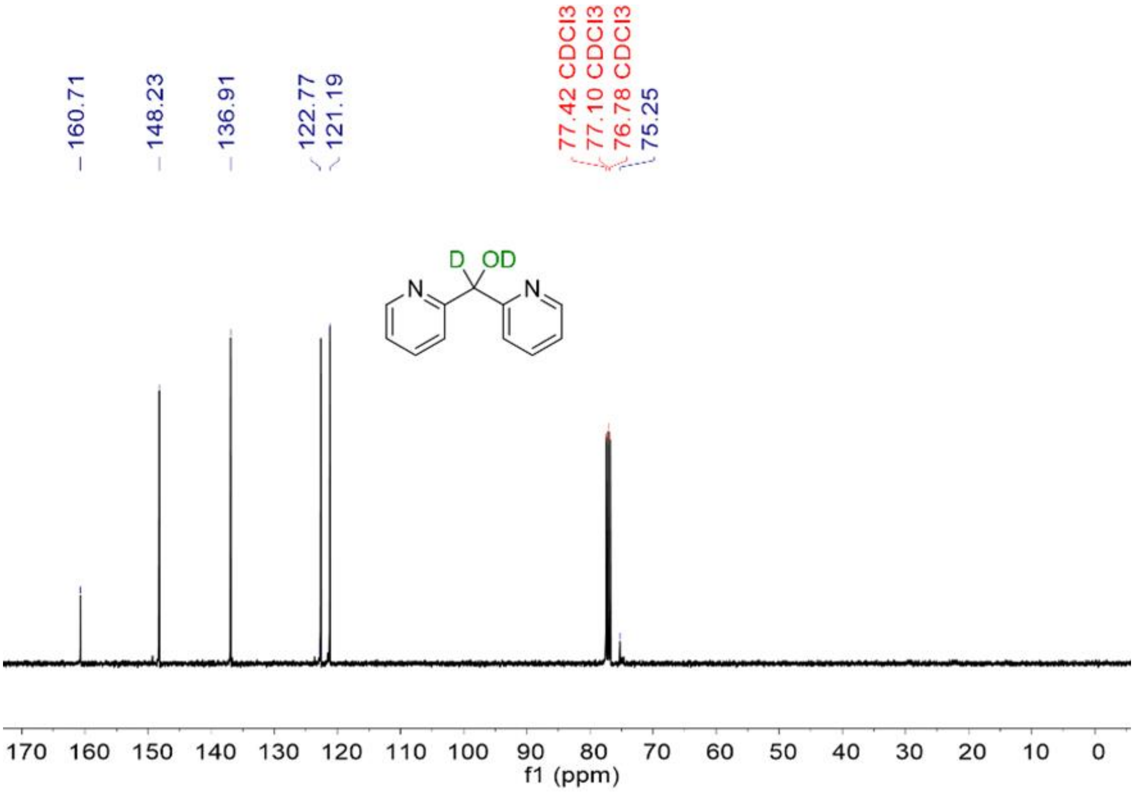

^{13}C NMR spectrum of **8** (100 MHz, $CDCl_3$).

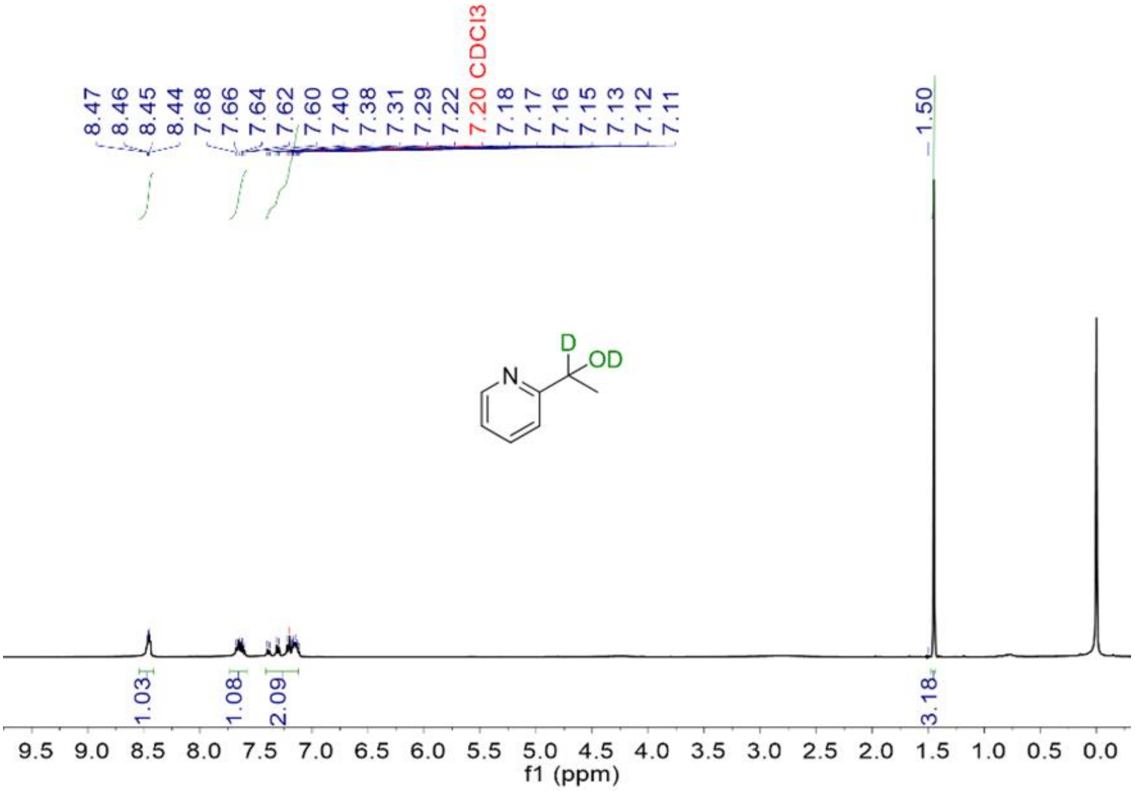

^{1}H NMR spectrum of **9** (400 MHz, $CDCl_3$).

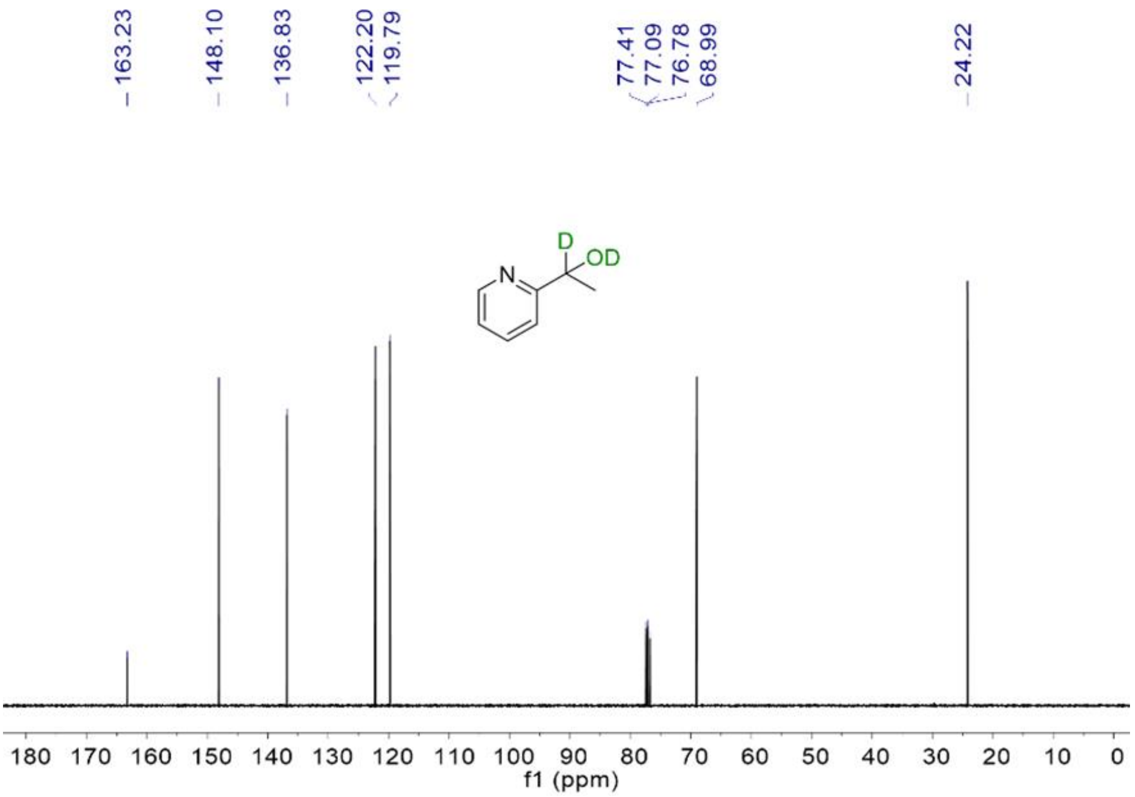

^{13}C NMR spectrum of **9** (100 MHz, $CDCl_3$).

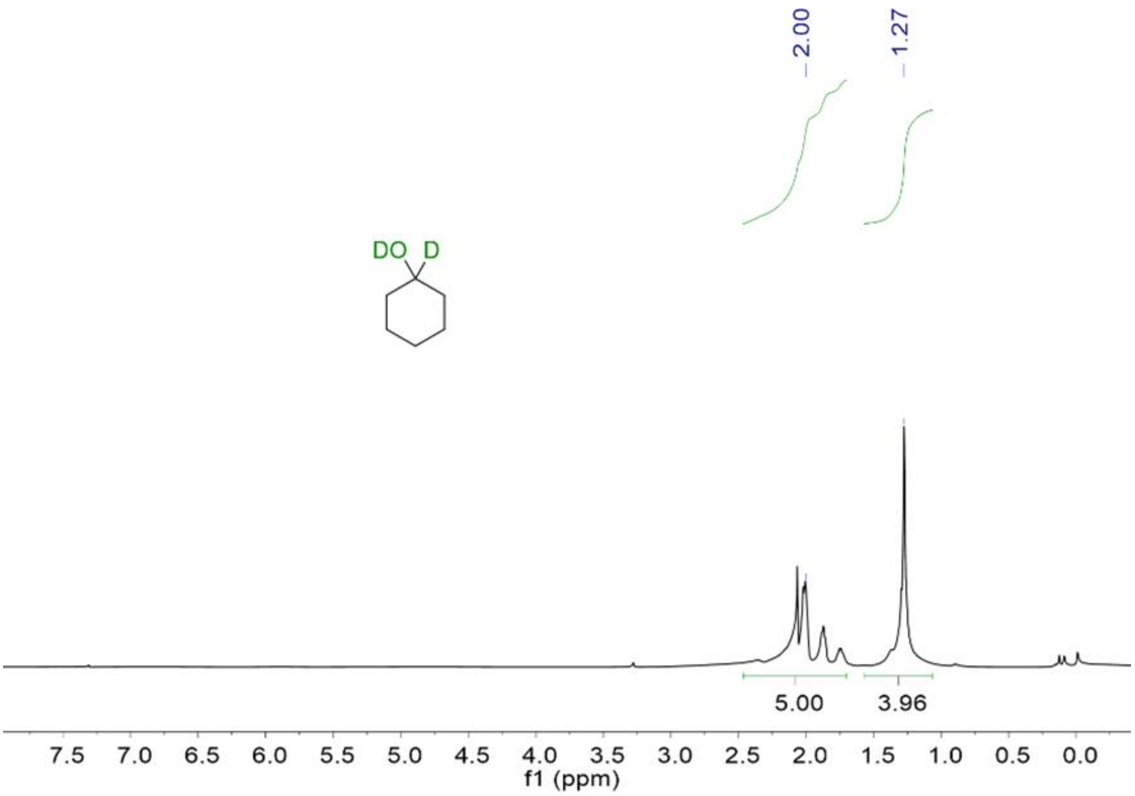

^{1}H NMR spectrum of **10** (400 MHz, $CDCl_3$).

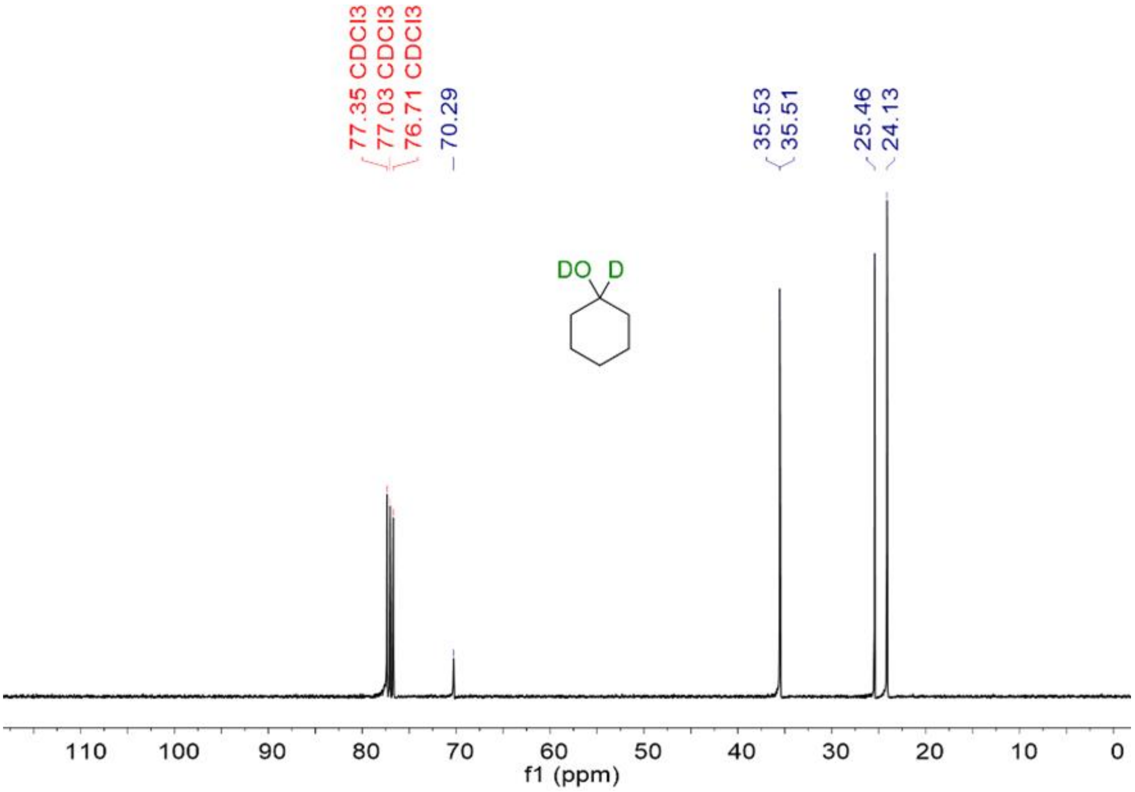

^{13}C NMR spectrum of **10** (100 MHz, $CDCl_3$).

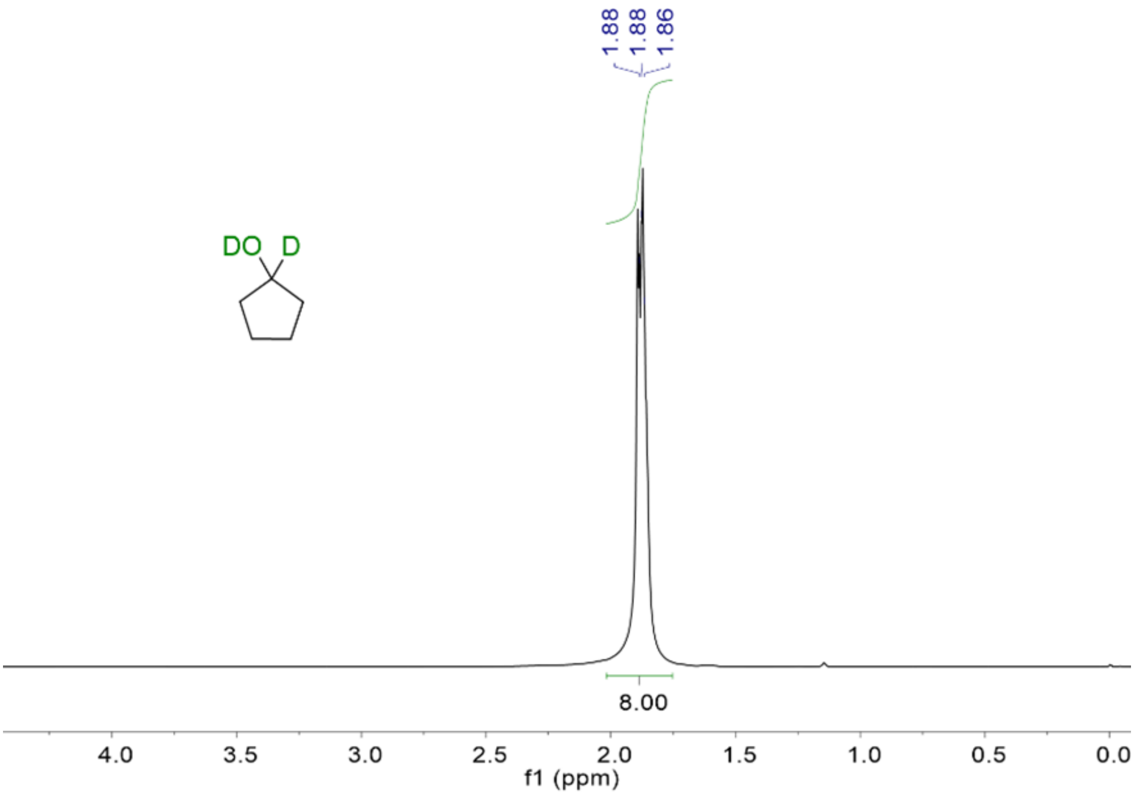

^{1}H NMR spectrum of **11** (400 MHz, $CDCl_3$).

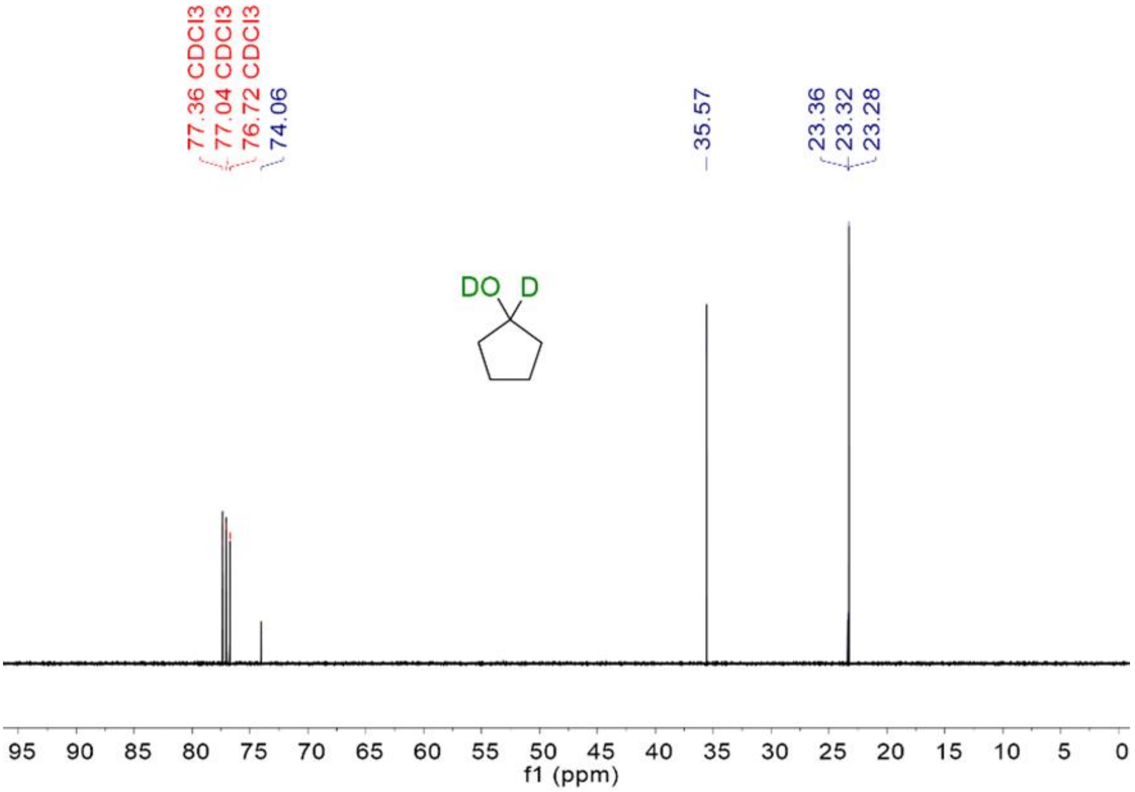

^{13}C NMR spectrum of **11** (100 MHz, $CDCl_3$).

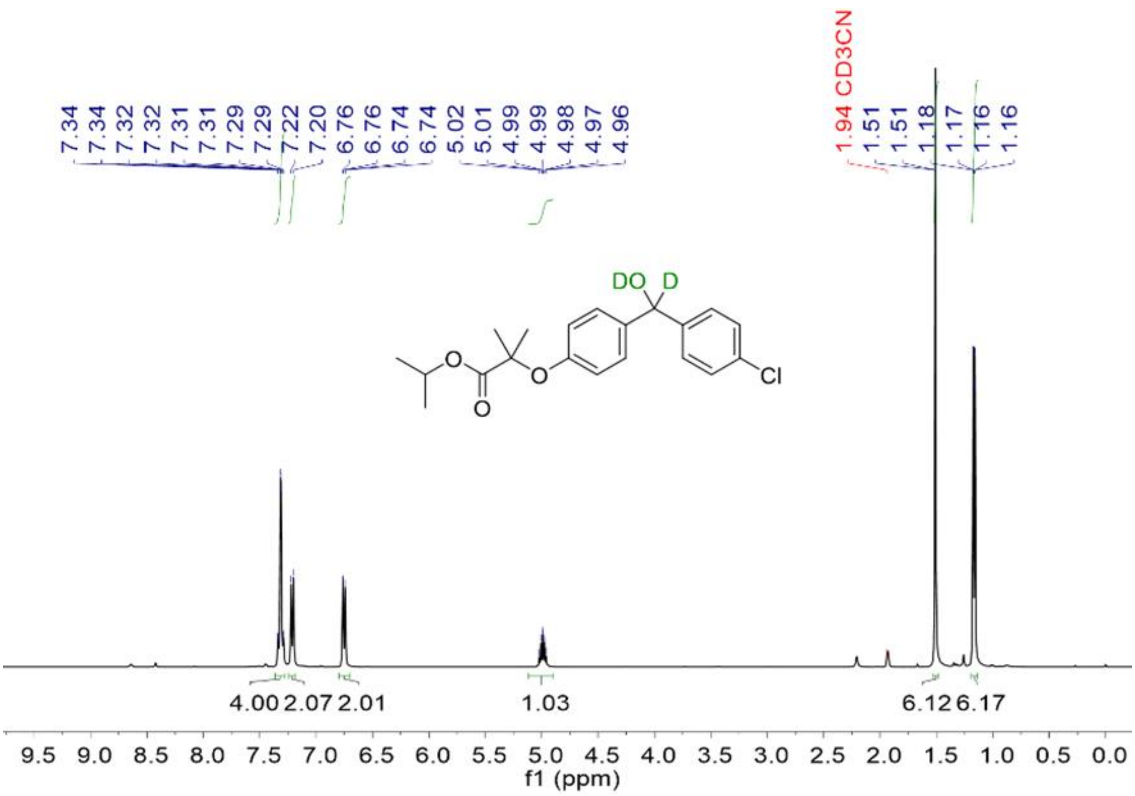

^{1}H NMR spectrum of **12** (400 MHz, CD_3CN).

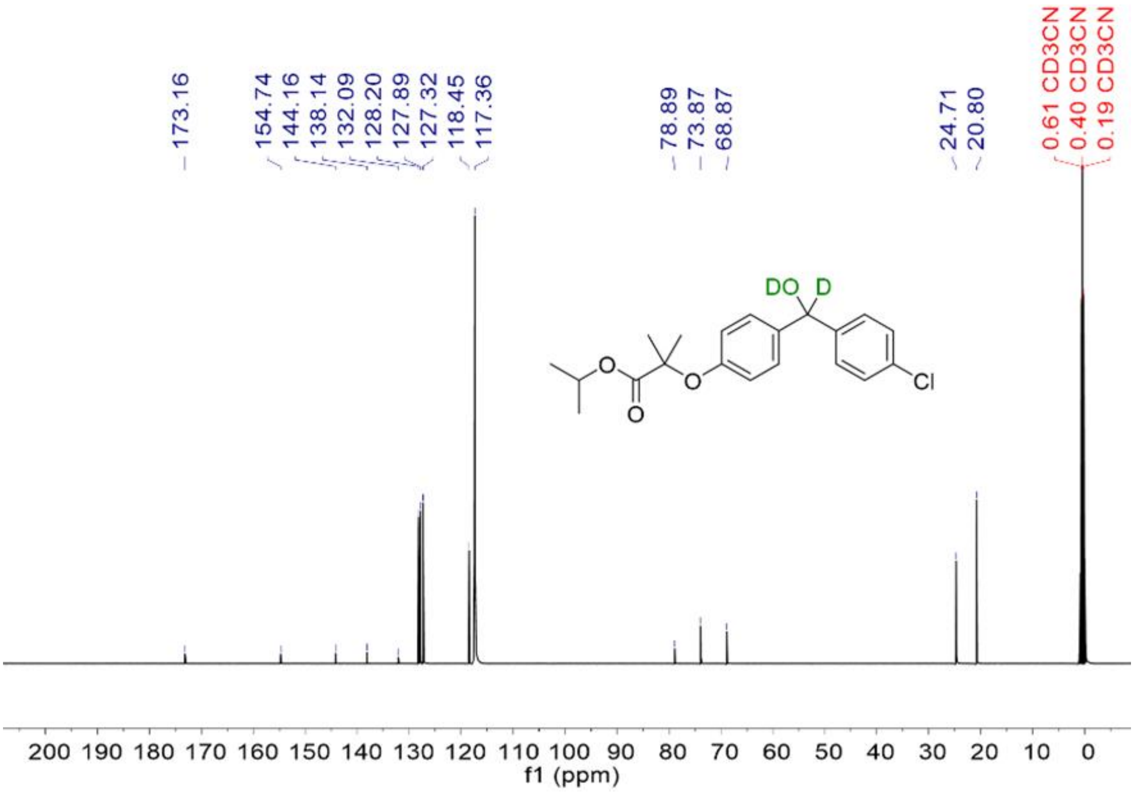

^{13}C NMR spectrum of **12** (100 MHz, CD_3CN).

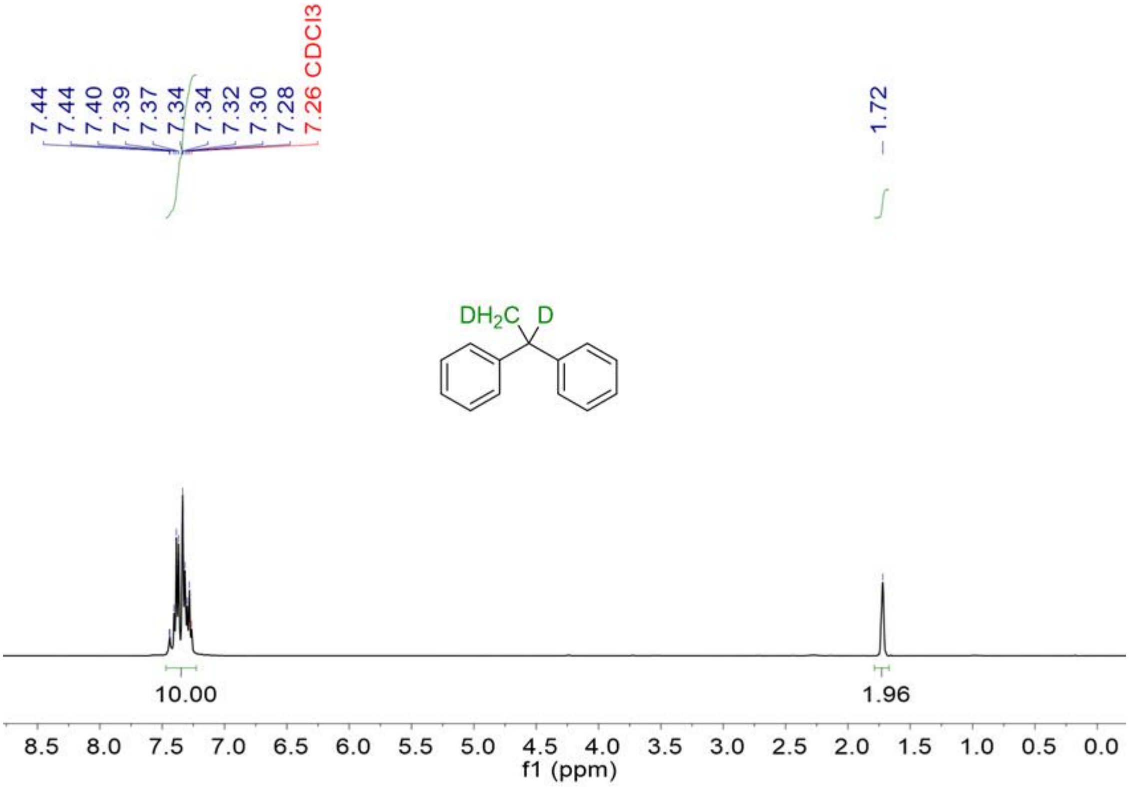

^{1}H NMR spectrum of **14** (400 MHz, $CDCl_3$).

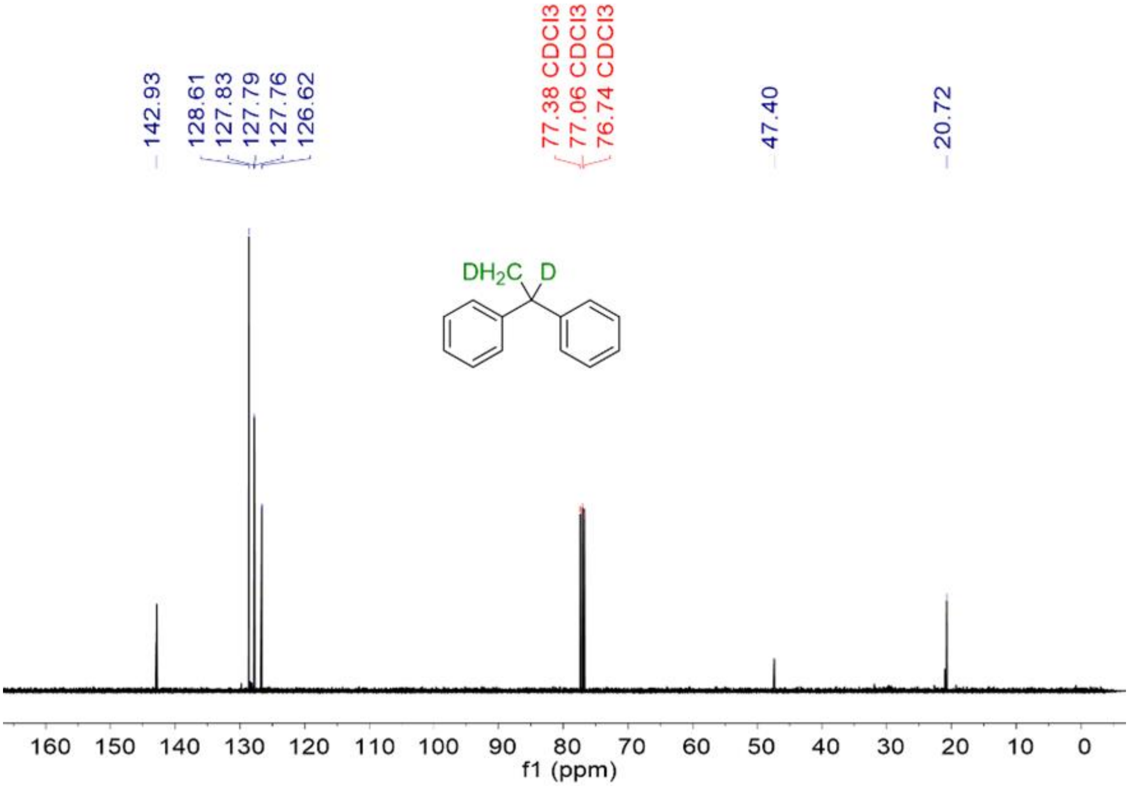

^{13}C NMR spectrum of **14** (100 MHz, $CDCl_3$).

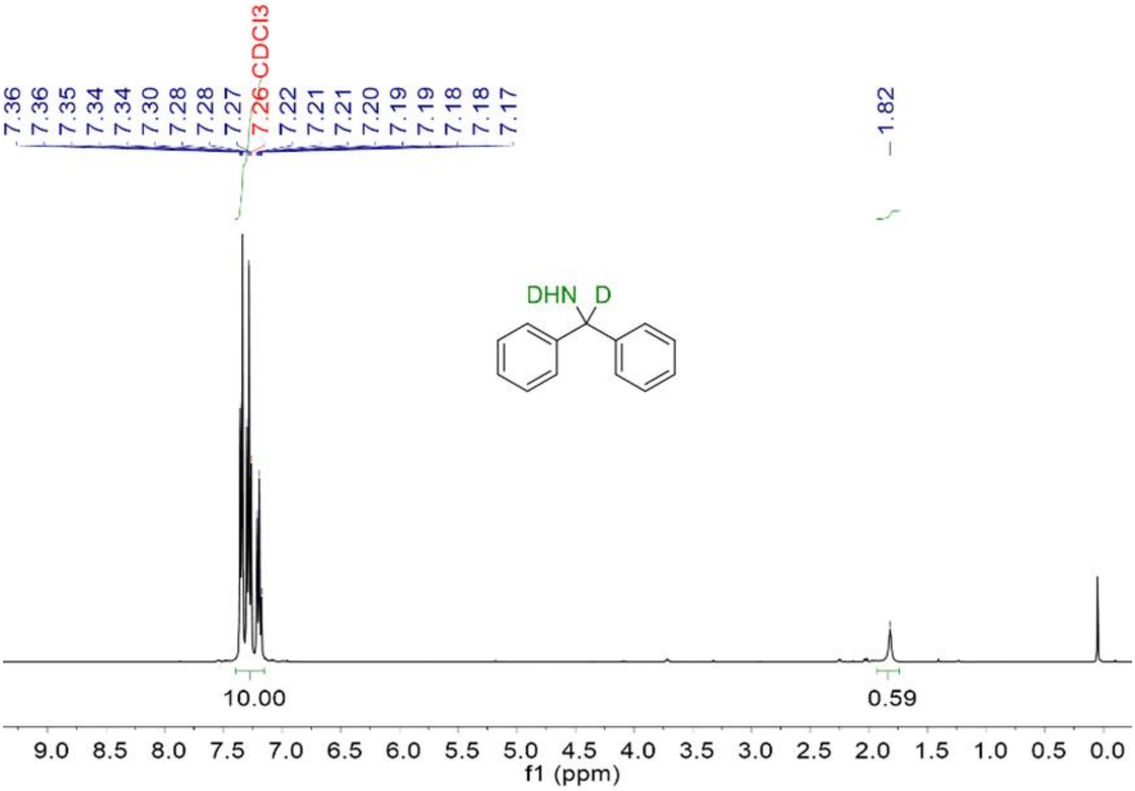

^{1}H NMR spectrum of **15** (400 MHz, $CDCl_3$).

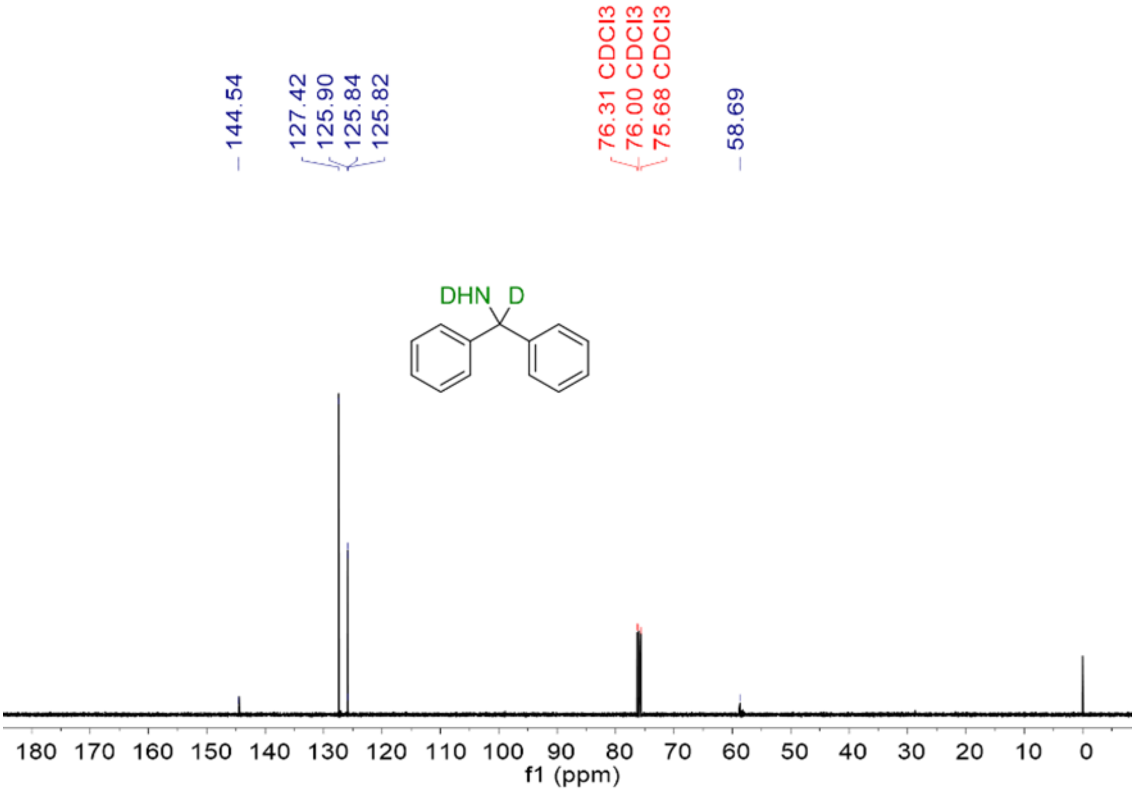

^{13}C NMR spectrum of **15** (100 MHz, $CDCl_3$).

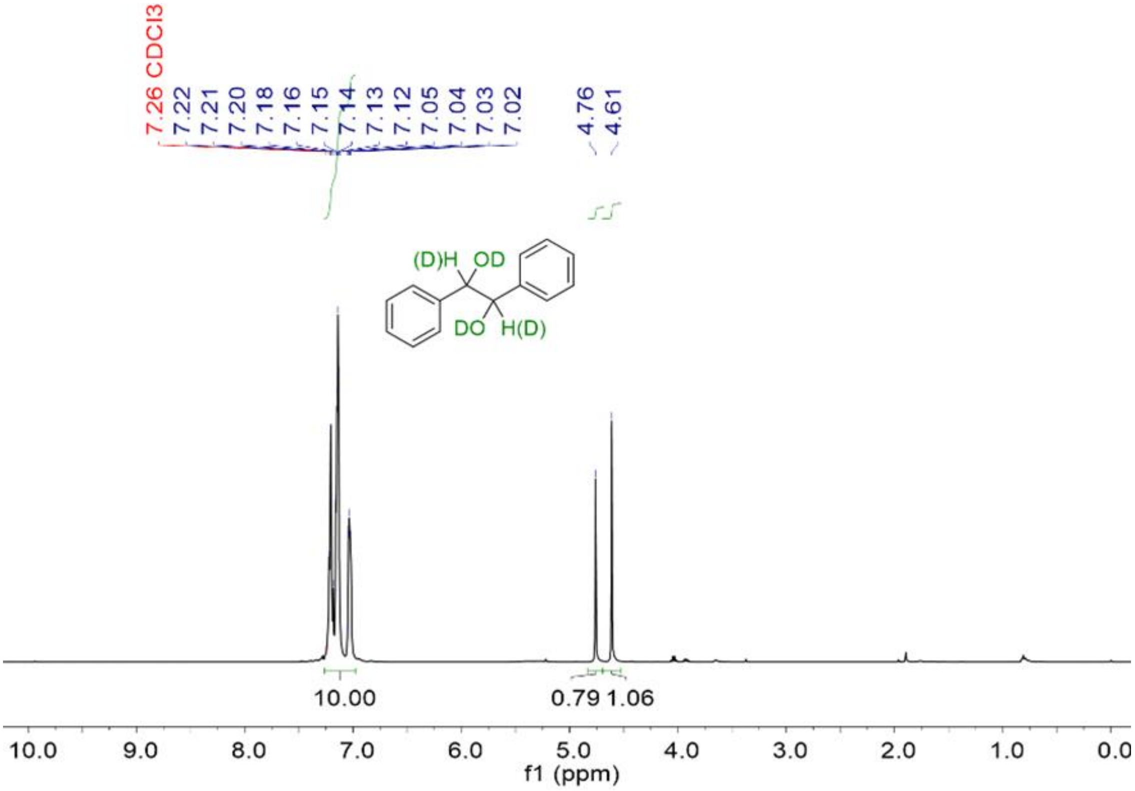

^{1}H NMR spectrum of **16** (400 MHz, $CDCl_3$).

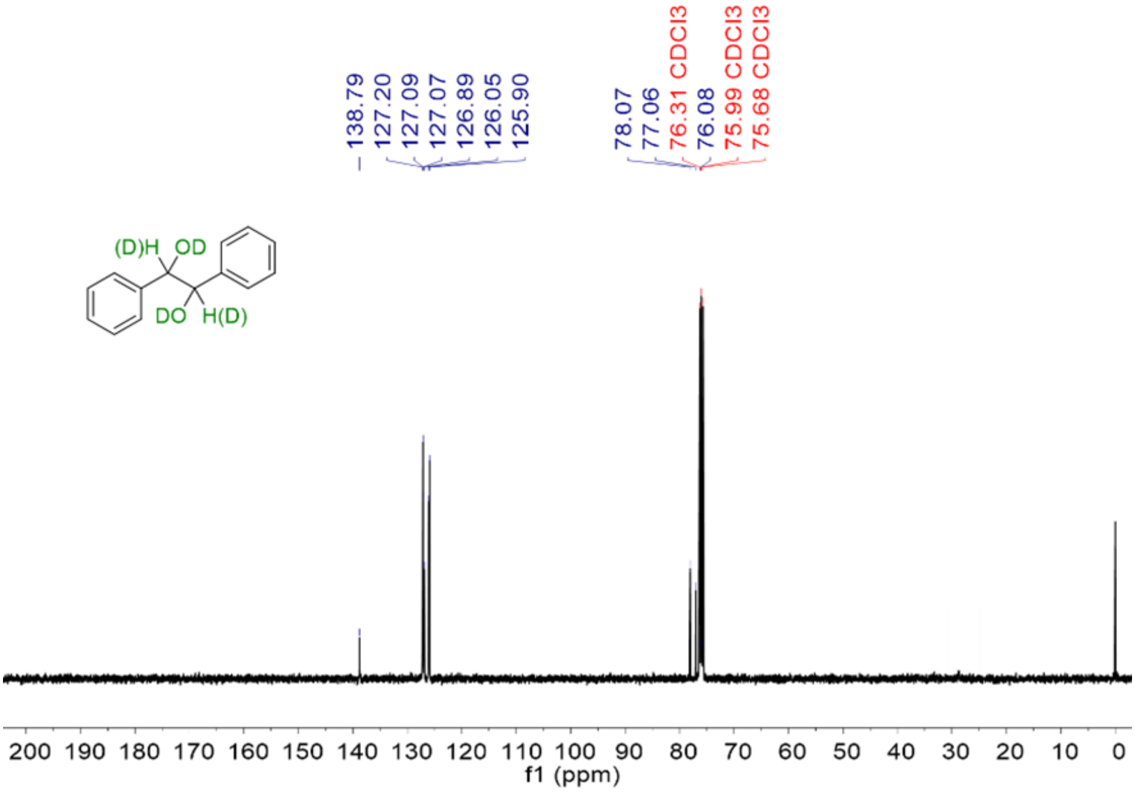

^{13}C NMR spectrum of **16** (100 MHz, $CDCl_3$).

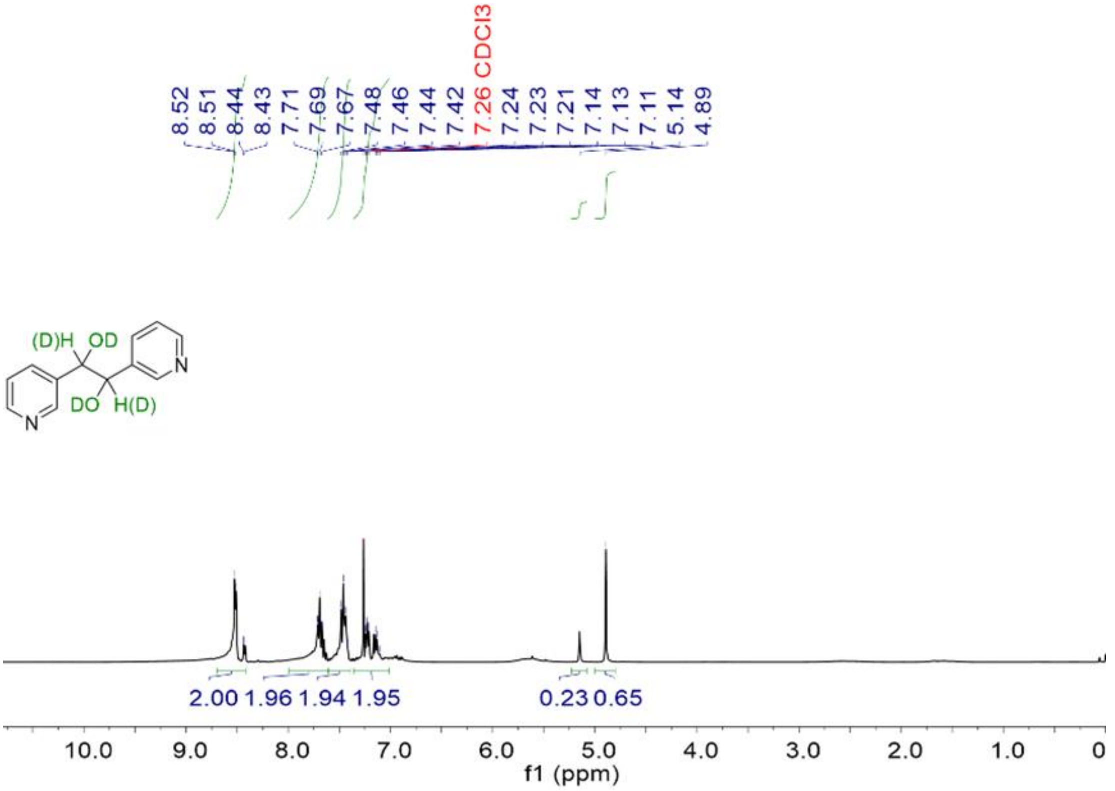

^{1}H NMR spectrum of **17** (400 MHz, $CDCl_3$).

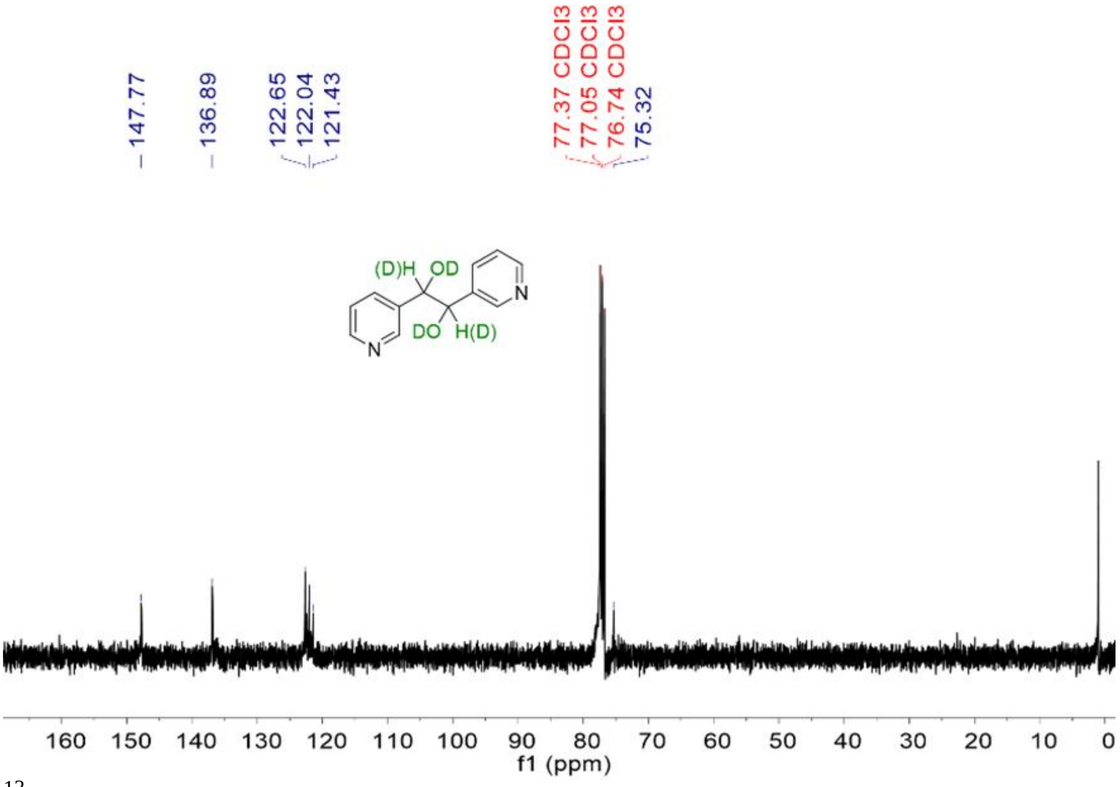

^{13}C NMR spectrum of **17** (100 MHz, $CDCl_3$).

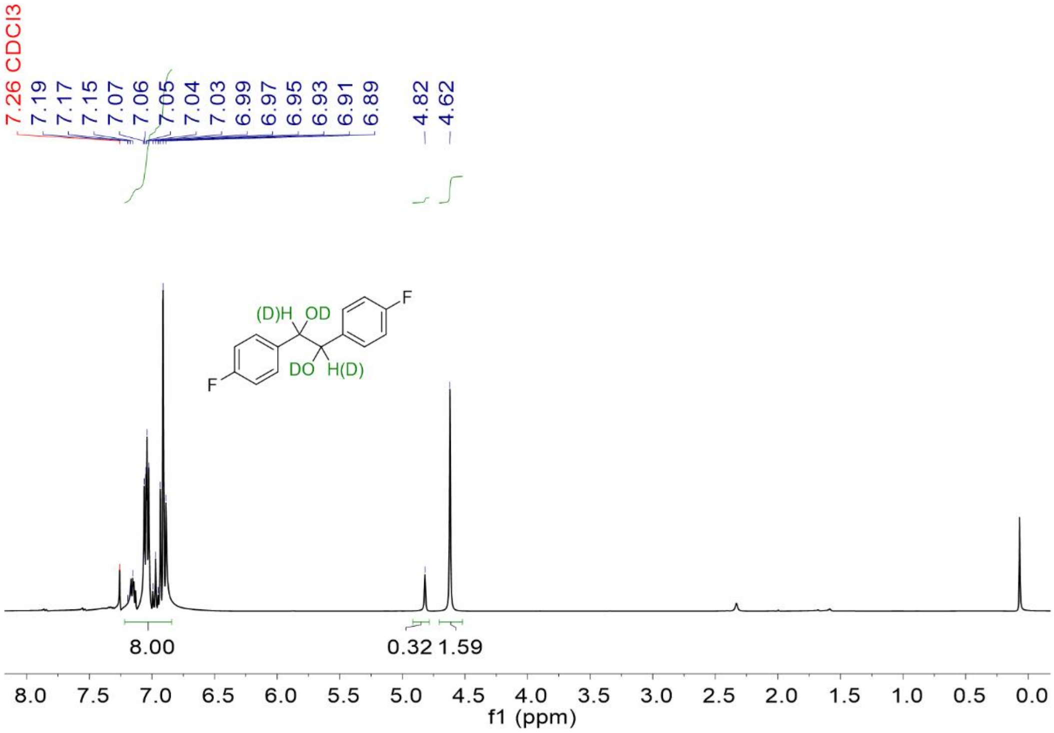

^{1}H NMR spectrum of **18** (400 MHz, $CDCl_3$).

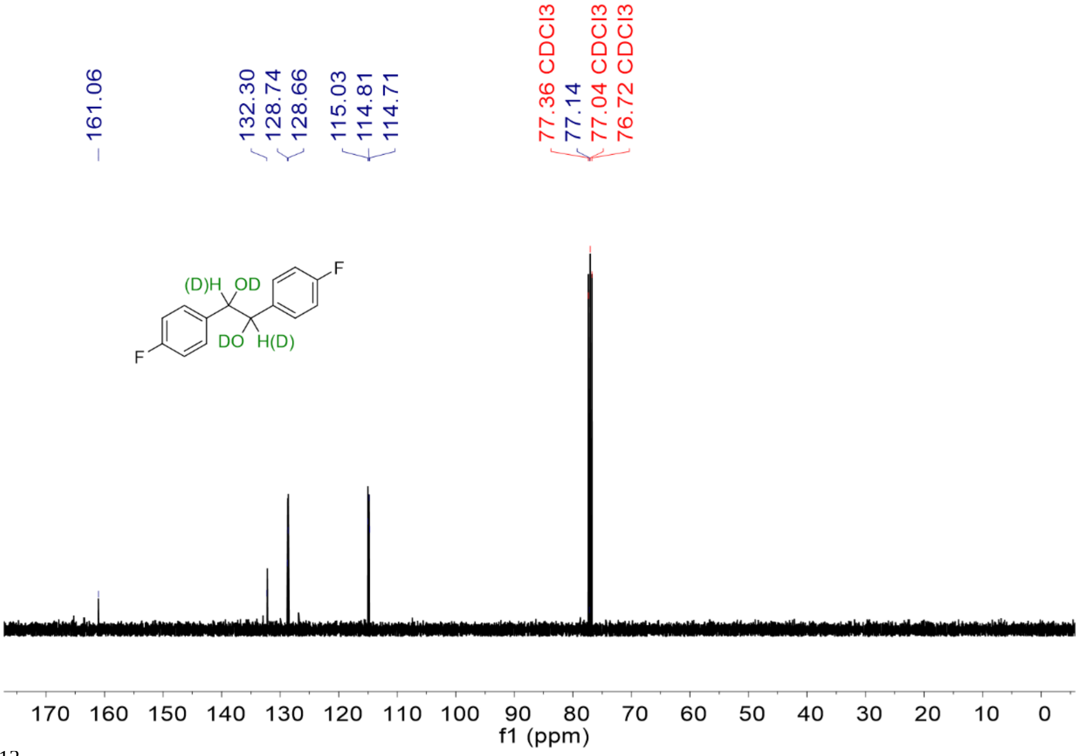

^{13}C NMR spectrum of **18** (100 MHz, $CDCl_3$).

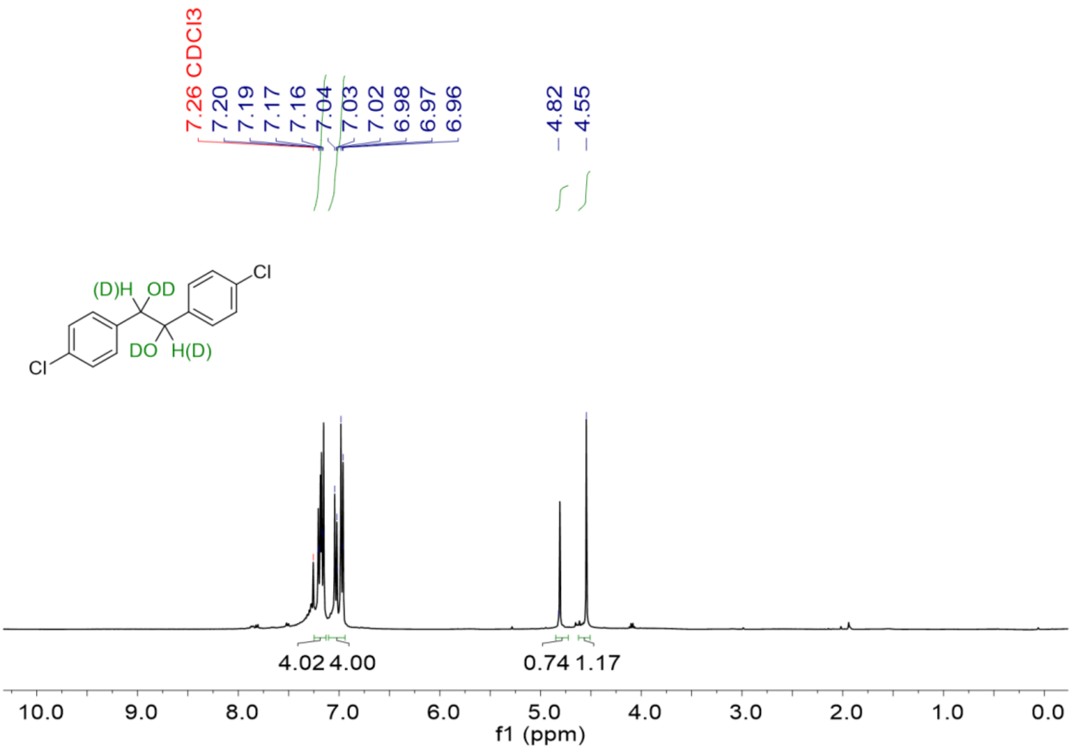

^{1}H NMR spectrum of **19** (400 MHz, $CDCl_3$).

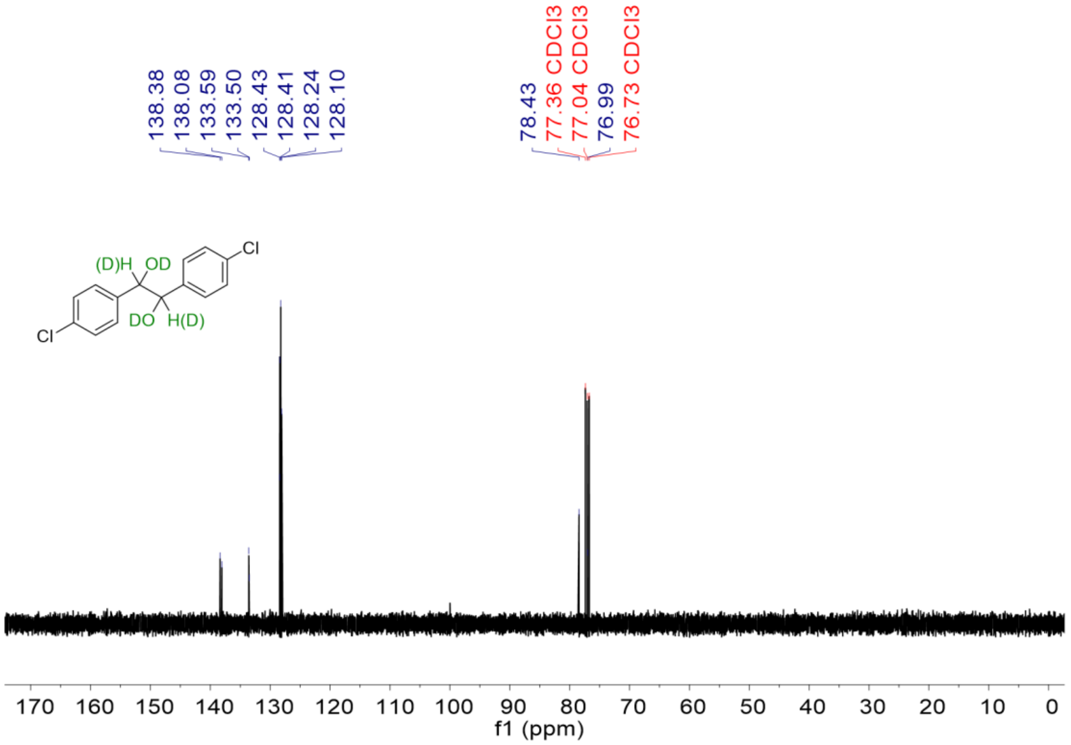

^{13}C NMR spectrum of **19** (100 MHz, $CDCl_3$).

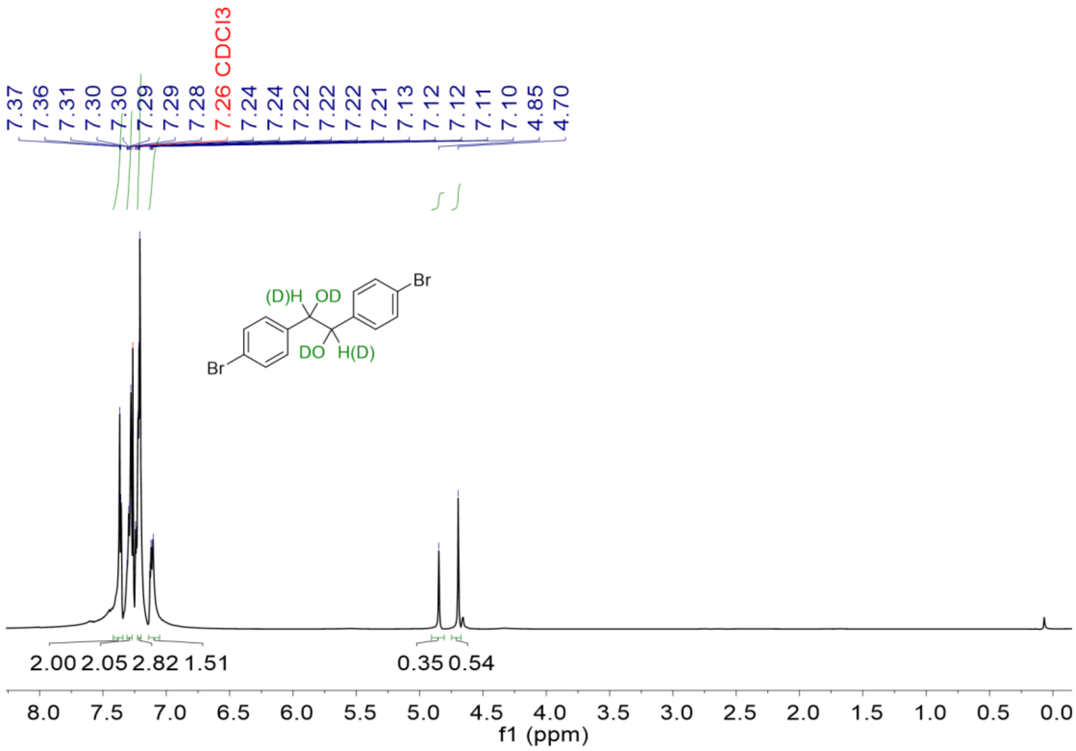

^{1}H NMR spectrum of **20** (400 MHz, $CDCl_3$).

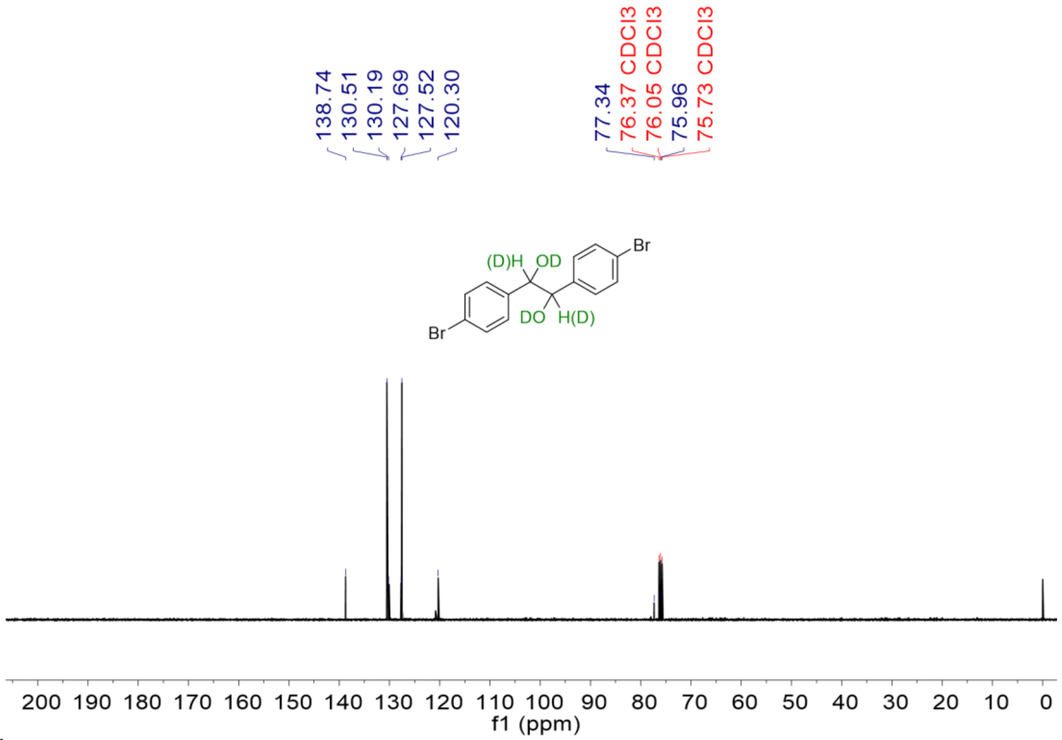

^{13}C NMR spectrum of **20** (100 MHz, $CDCl_3$).

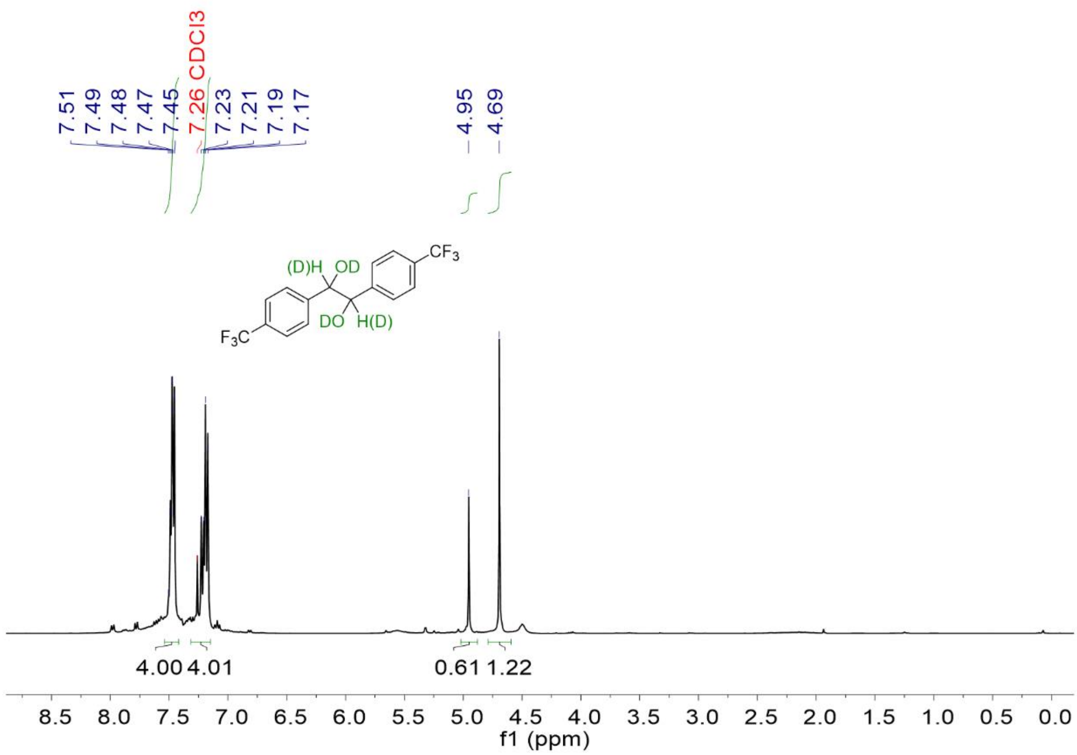

^{1}H NMR spectrum of **21** (400 MHz, $CDCl_3$).

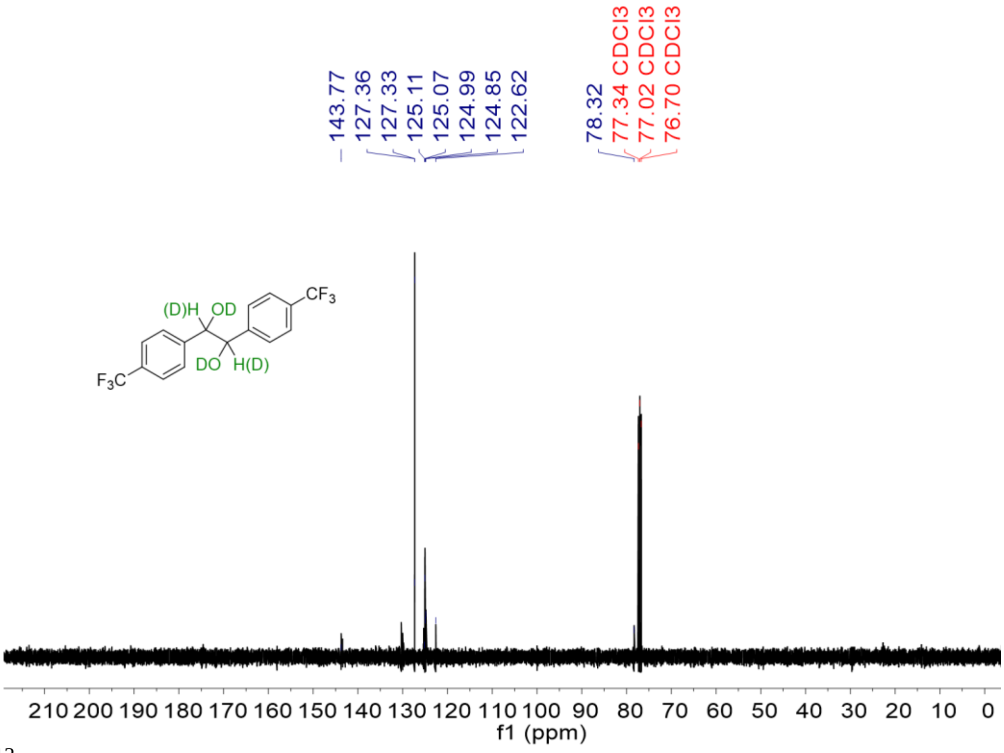

^{13}C NMR spectrum of **21** (100 MHz, $CDCl_3$).

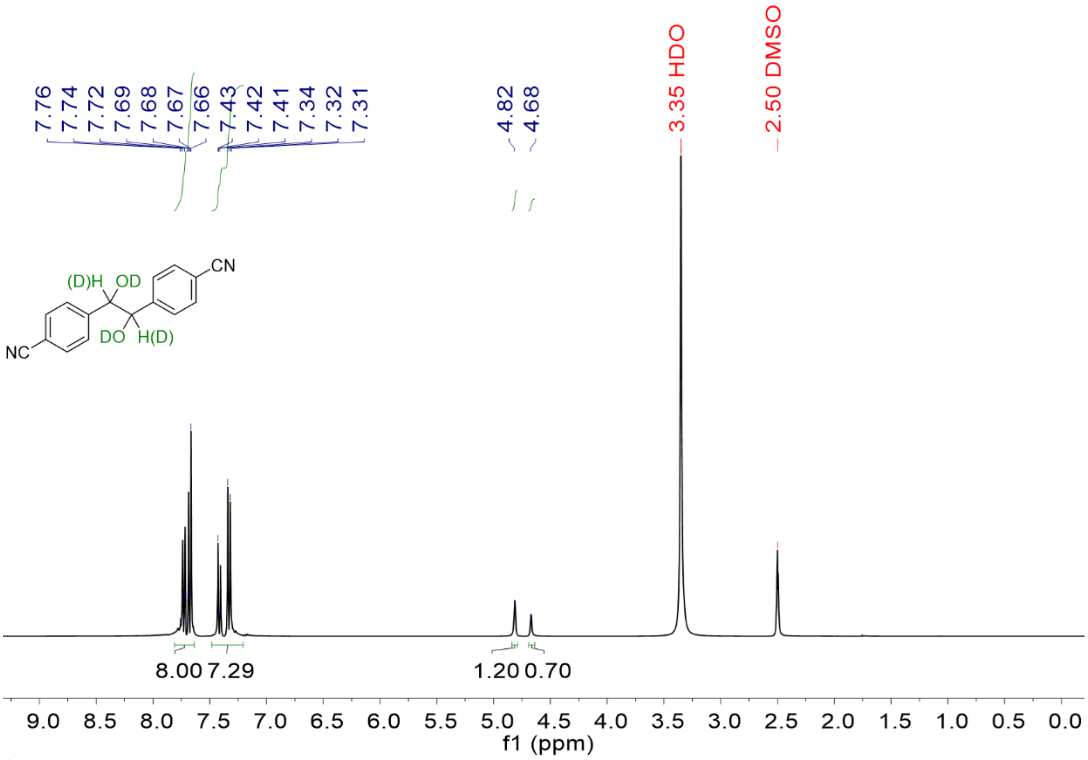

^{1}H NMR spectrum of **22** (400 MHz, DMSO-d_6).

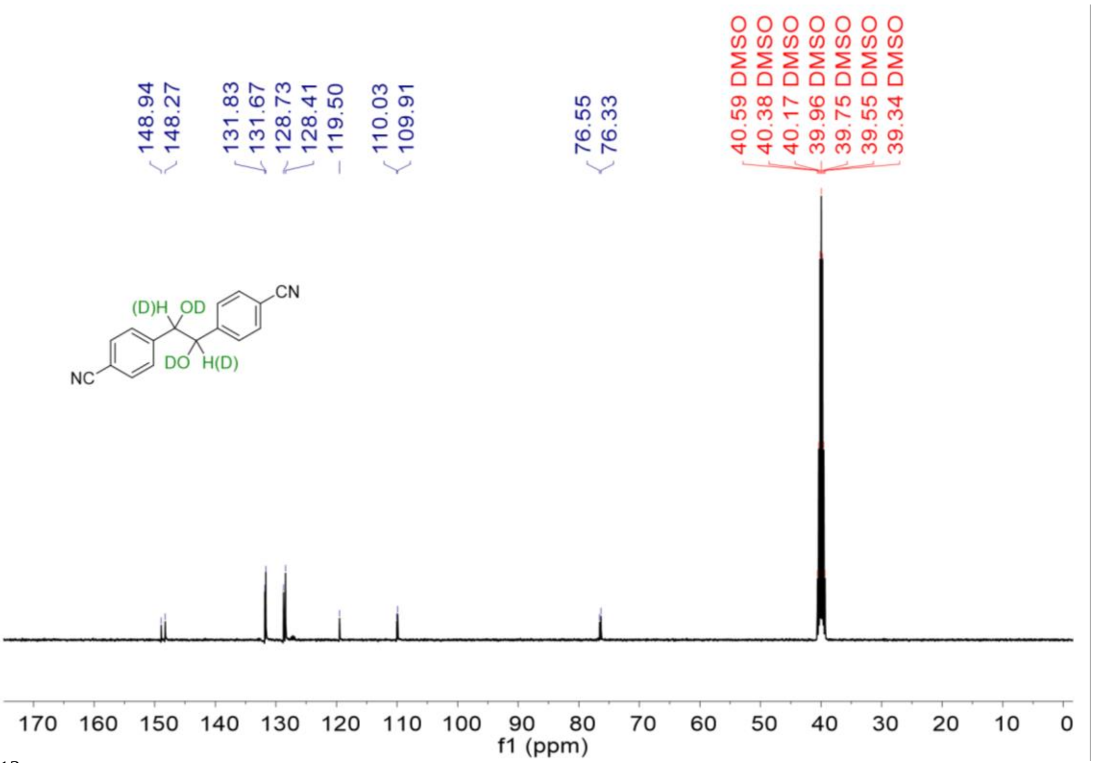

^{13}C NMR spectrum of **22** (100 MHz, DMSO-d_6).

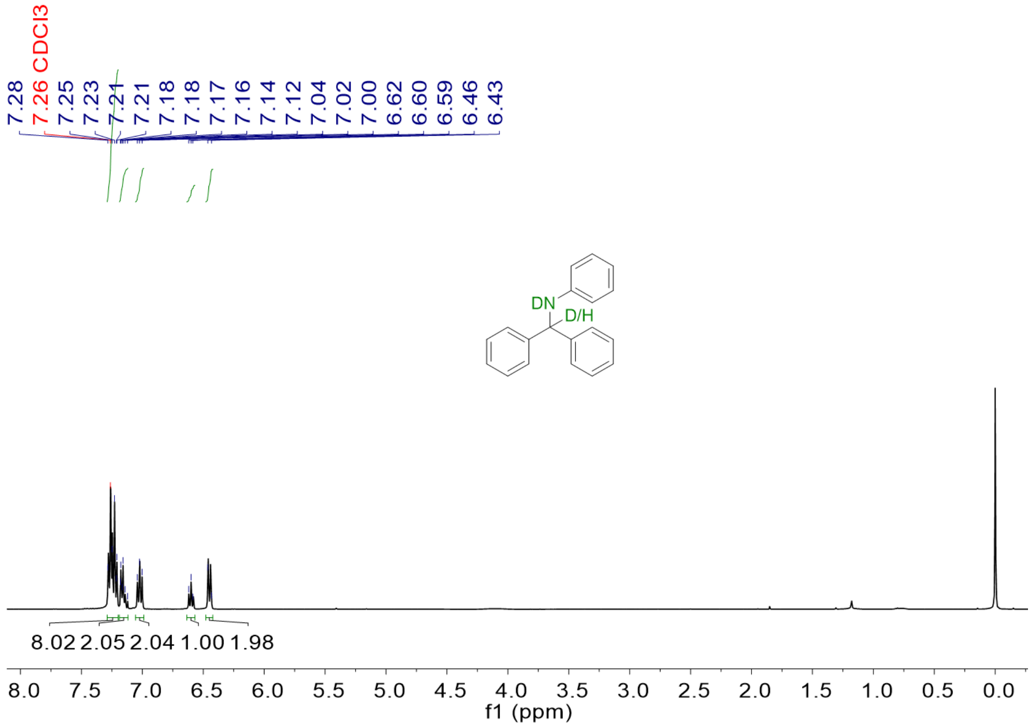

^{1}H NMR spectrum of deuterated N-benzhydrylaniline (400 MHz, $CDCl_3$).

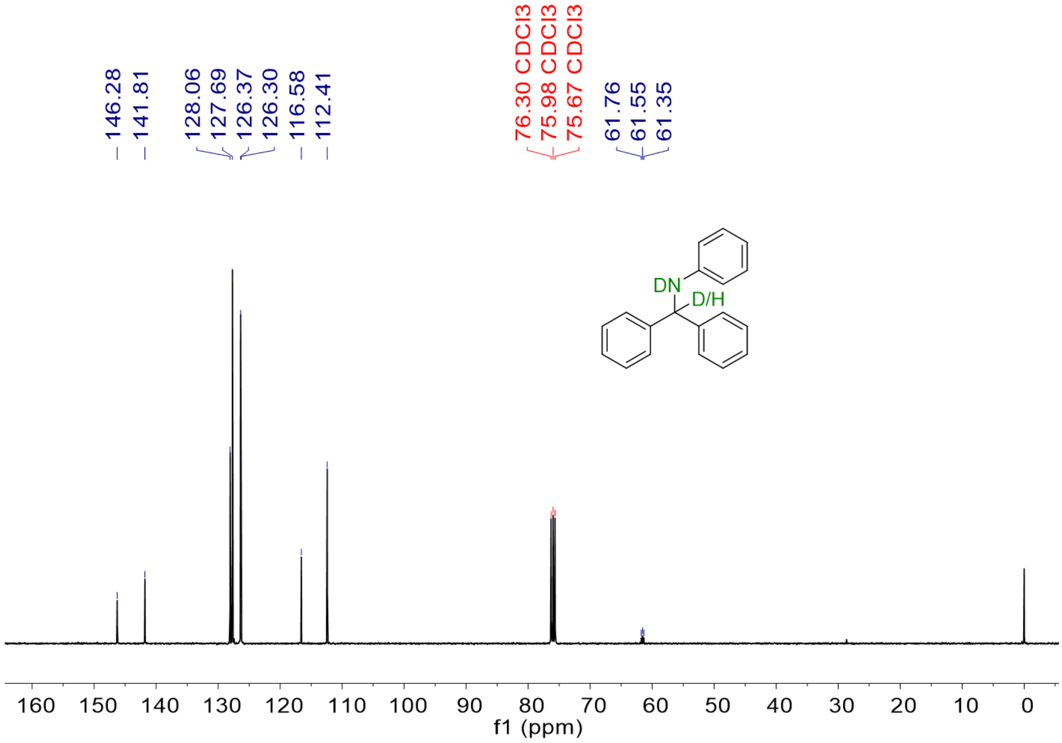

^{13}C NMR spectrum of deuterated N-benzhydrylaniline (400 MHz, $CDCl_3$).

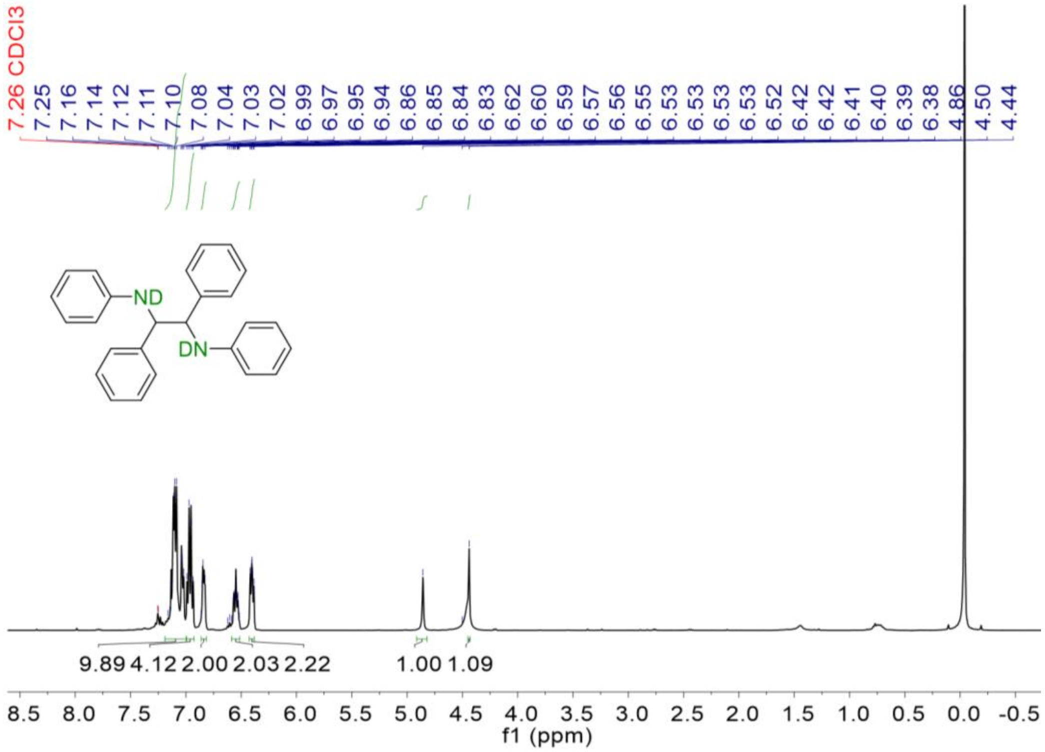

^{1}H NMR spectrum of deuterated 1,2-dianilinobibenzyl (400 MHz, $CDCl_3$).

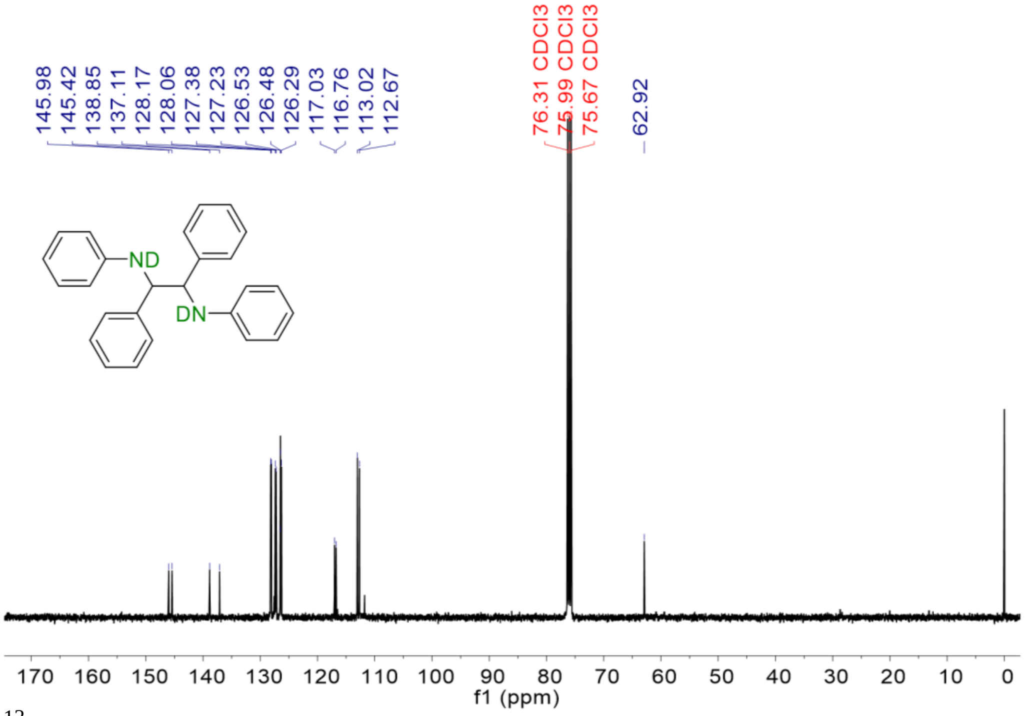

^{13}C NMR spectrum of deuterated 1,2-dianilinobibenzyl (400 MHz, $CDCl_3$).

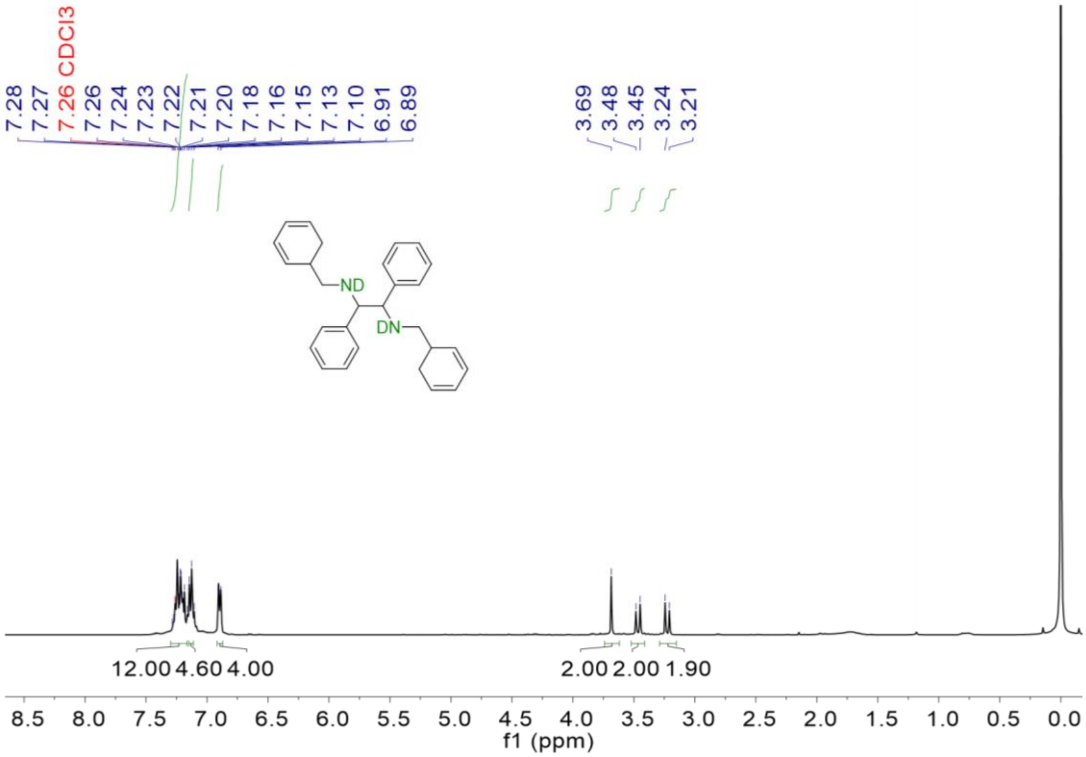

^{1}H NMR spectrum of deuterated N,N'-dibenzyl-1,2-diphenylethane-1,2-diamine (400 MHz, $CDCl_3$).

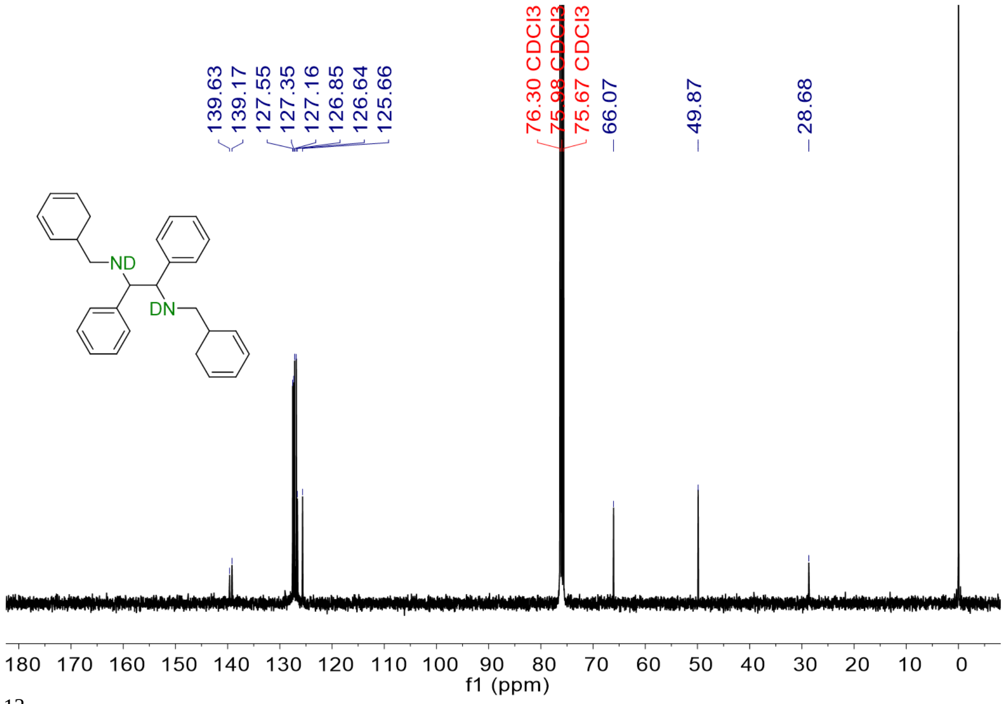

^{13}C NMR spectrum of deuterated N,N'-dibenzyl-1,2-diphenylethane-1,2-diamine (400 MHz, $CDCl_3$).

References

[1] C. Han, X. Zhu, J. S. Martin, Y. Lin, S. Spears, Y. Yan, *ChemSusChem* **2020**, *13*, 4005.

[2] a) D. Zeng, T. Zhou, W.-J. Ong, M. Wu, X. Duan, W. Xu, Y. Chen, Y.-A. Zhu, D.-L. Peng, *ACS Appl. Mater. Interfaces* **2019**, *11*, 5651; b) L. Xu, Q. Li, X.-F. Li, M.-Q. Long, T. Chen, B. Peng, L.-L. Wang, Y. Yang, C. Shuai, *Catal. Sci. Technol.* **2019**, *9*, 3896; c) H. Zhang, Y. Dong, S. Zhao, G. Wang, P. Jiang, J. Zhong, Y. Zhu, *Appl. Catal. B* **2020**, *261*, 118233; d) Z. Zhou, F. Han, L. Guo, O. V. Prezhdo, *Phys. Chem. Chem. Phys.* **2016**, *18*, 16862; e) D. Luo, L. Peng, Y. Wang, X. Lu, C. Yang, X. Xu, Y. Huang, Y. Ni, *J. Mater. Chem. A* **2021**, *9*, 908; f) C. Han, Y.-H. Li, J.-Y. Li, M.-Y. Qi, Z.-R. Tang, Y.-J. Xu, *Angew. Chem. Int. Ed.* **2021**, *60*, 7962.

[3] a) A. Kurimoto, R. S. Sherbo, Y. Cao, N. W. X. Loo, C. P. Berlinguette, *Nature Catal.* **2020**, *3*, 719; b) C. Liu, S. Han, M. Li, X. Chong, B. Zhang, *Angew. Chem. Int. Ed.* **2020**, *59*, 18527.

[4] G. Kresse, J. Hafner, *Phys. Rev. B* **1994**, *49*, 14251.

[5] J. P. Perdew, K. Burke, M. Ernzerhof, *Phys. Rev. Lett.* **1996**, *77*, 3865.

[6] P. E. Blöchl, *Phys. Rev. B* **1994**, *50*, 17953.

[7] A. Mannodi-Kanakkithodi, J.-S. Park, N. Jeon, D. H. Cao, D. J. Gosztola, A. B. Martinson, M. K. Chan, *Chem. Mater.* **2019**, *31*, 3599.

[8] a) G. Sai Gautam, T. P. Senftle, E. A. Carter, *Chem. Mater.* **2018**, *30*, 4543; b) F. Fuchs, F. Bechstedt, *Phys. Rev. B* **2008**, *77*, 155107.
